# Unraveling the Mechanism of the Ir^III^‐Catalyzed Regiospecific Synthesis of α‐Chlorocarbonyl Compounds from Allylic Alcohols

**DOI:** 10.1002/chem.202002845

**Published:** 2020-10-14

**Authors:** Man Li, Amparo Sanz‐Marco, Samuel Martinez‐Erro, Víctor García‐Vázquez, Binh Khanh Mai, Jacob Fernández‐Gallardo, Fahmi Himo, Belén Martín‐Matute

**Affiliations:** ^1^ Department of Organic Chemistry Stockholm University 10691 Stockholm Sweden

**Keywords:** allylic alcohols, catalytic cycle, density functional calculations, functionalizations, iridium catalysis, isomerization

## Abstract

We have used experimental studies and DFT calculations to investigate the Ir^III^‐catalyzed isomerization of allylic alcohols into carbonyl compounds, and the regiospecific isomerization–chlorination of allylic alcohols into α‐chlorinated carbonyl compounds. The mechanism involves a hydride elimination followed by a migratory insertion step that may take place at Cβ but also at Cα with a small energy‐barrier difference of 1.8 kcal mol^−1^. After a protonation step, calculations show that the final tautomerization can take place both at the Ir center and outside the catalytic cycle. For the isomerization–chlorination reaction, calculations show that the chlorination step takes place outside the cycle with an energy barrier much lower than that for the tautomerization to yield the saturated ketone. All the energies in the proposed mechanism are plausible, and the cycle accounts for the experimental observations.

## Graphical abstract

**Experimental and DFT calculations** have been used to understand the mechanism of the Ir^III^‐catalyzed isomerization of allylic alcohols into carbonyl compounds, and the regiospecific isomerization–chlorination of allylic alcohols into α‐chlorinated carbonyl compounds. The results presented, point to the importance of using an experimental–computational approach to comprehend the mechanisms of complex reactions (see scheme).

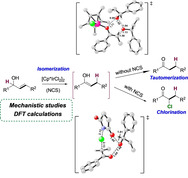

## Introduction

The isomerization of allylic alcohols to obtain carbonyl compounds is an atom‐economical strategy that has been extensively used in organic synthesis.^[1]^

**Scheme 1:**
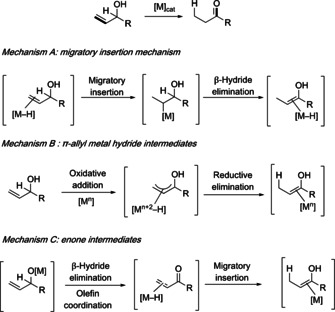
General mechanisms for the isomerization of allylic alcohols.

Compared with the classical two‐step approach involving oxidation–reduction, or vice versa, this efficient method gives access to aldehydes or ketones in a single step from readily available allylic alcohols. Metal complexes of rhodium,^[2]^ iridium,^[3]^ ruthenium,^[4]^ palladium,^[5]^ iron,^[6]^ or cobalt^[7]^ can catalyze this reaction. Recently, organocatalysts have also been used in this transformation.^[8]^ In general terms, the mechanism of the isomerization involves migration of the carbon–carbon double bond with a concomitant [1,3]‐hydrogen shift to form an enol(ate). Three general mechanisms for transition‐metal‐mediated isomerizations are considered in the literature (Scheme 1); a) a metal‐hydride addition‐elimination pathway, b) a pathway via π‐allyl metal‐hydride intermediates; c) and a pathway via metal‐alkoxy catalytic species, leading to enone intermediates. The actual reaction mechanism may depend on the reaction conditions, as well as on the nature of the metal catalysts and substrates.[^2^, ^3^, ^4^, ^5^, ^6^, ^7^]

Density‐functional theory (DFT) calculations have been used to elucidate the mechanisms of transition‐metal catalyzed isomerization reactions of allylic alcohols. In 2003, Branchadell, Grée, and co‐workers proposed a mechanism involving π‐allyl hydride intermediates for the reaction catalyzed by Fe(CO)_3_.^[6c]^ Cadierno, Gimeno, Sordo, and co‐workers reported a theoretical study of the isomerization catalyzed by Ru^IV^ complexes;^[4b]^ they concluded that in this case the catalytic activity involved the chelated coordination of the allylic alcohol to the metal through the oxygen and the double bond. Mazet and co‐workers investigated the reaction using Ir^I^ and Pd^II^ precatalysts, which are converted into metal hydrides before the start of the reaction, and they proposed a metal‐hydride addition‐elimination mechanism.[^3b^, ^6b^]

**Scheme 2:**
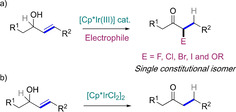
Iridium‐catalyzed isomerization and tandem isomerization–functionalization of allylic alcohols.

Since the isomerization mechanism proceeds via a series of intermediates, it may be possible to harness these species and use them in further transformations. In the last few years, we have reported the conversion of allylic alcohols into a series of α‐functionalized carbonyl compounds by using simple metal complexes based on the [Cp*Ir^III^] structure, and carrying out the reaction in the presence of different electrophilic halogenating and oxygenating agents (Scheme 2 a).^[9]^ Importantly, the products are obtained as single constitutional isomers, which rules out a pathway involving simple isomerization (as in Scheme 1) followed by in situ functionalization of the resulting ketone.

Interestingly, when the reaction is carried out under the same conditions in the absence of an electrophile, the same catalyst, i.e., [Cp*IrCl_2_]_2_, does also catalyze the isomerization of allylic alcohols into carbonyl compounds with outstanding levels of efficiency; the reaction can take place at room temperature under base‐free conditions, and its scope is very large (Scheme 2 b).^[10]^ It is therefore rather remarkable that the reactions leading to α‐functionalized carbonyl compounds take place with such high levels of selectivity, as unfunctionalized carbonyl compounds are, in the worst cases, formed in only trace amounts. For example, when *N*‐chlorosuccinimide (NCS) is used as the electrophile, the α‐chlorinated carbonyl compounds are formed exclusively in excellent yields.^[9b]^

In this paper, we describe our work towards understanding the mechanism of the Ir^III^‐catalyzed regiospecific conversion of allylic alcohols into α‐chlorocarbonyl compounds catalyzed by [Cp*IrCl_2_]_2_ through a combined experimental and computational approach.

## Results and Discussion

### Experimental mechanistic investigations of the Ir^III^‐catalyzed isomerization of allylic alcohols

**Scheme 3:**
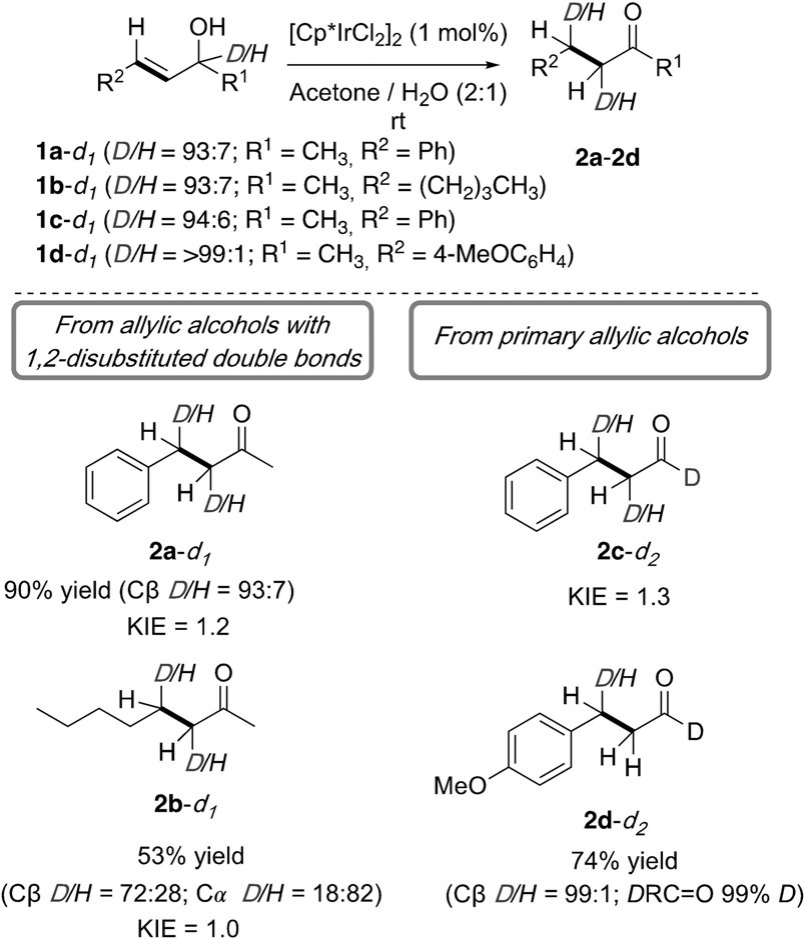
Previous mechanistic studies for the isomerization of allylic alcohols. Reactions were carried out on a 0.1 mmol scale at 0.1 m at r.t. for 2.5 h. Yields of isolated products are shown.

We have previously carried out a number of experimental studies to understand the mechanism of the simple isomerization reaction (Scheme 2 b, and Scheme 3).^[10]^ We concluded that the active catalyst must have at least a halide ligand. This fact was supported by X‐ray absorption spectroscopy (XAS) and mass spectrometry (MS) studies. Our experimental investigations led us to propose a mechanism for the simple isomerization involving enone intermediates (Scheme 1 c). A summary of the experiments carried out is given in Scheme 3. Isotopic labeling investigations (alcohols **1 a**–**1 d**) and noncompetitive KIEs (alcohols **1 a**–**1 c**) were carried out. We observed no significant KIE for all the substrates tested. The amount of deuterium found in the products corresponded well with the deuterium content in the starting materials. Deuterium was found exclusively at Cβ for *sec*‐allylic alcohols **1 a**–***d***_***1***_ and primary allylic alcohol **1 d**–***d***_***2***_. However, for *sec*‐allylic alcohols **1 b**–***d***_***1***_, 18 % of deuterium was also transferred to Cα. Furthermore, several crossover experiments were carried out, and no deuterium scrambling was observed between the substrates (not shown).^[10]^

**Scheme 4:**
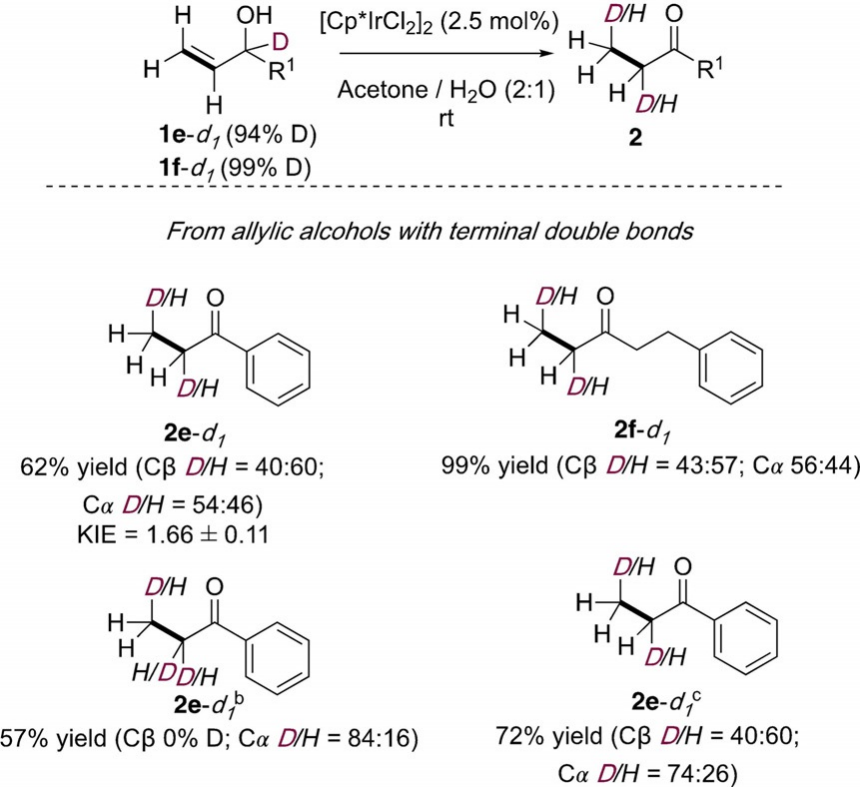
Mechanistic studies for the isomerization of allylic alcohols bearing terminal double bonds. [a] Reactions were carried out on a 0.1 mmol scale, in acetone/H_2_O (2:1, v/v; 0.1 m), at r.t. for 3 h. Yields of isolated products are shown. [b] From non‐deuterated **1 e** in acetone/D_2_O (2:1, v/v) for 3 h. [c] From deuterated **1 e**–***d***_***1***_ in acetone/D_2_O (2:1, v/v) for 3 h.

To gain further insight into the mechanism, the deuterium‐labeling experiments have now been expanded to allylic alcohols bearing terminal double bonds (Scheme 4).

In contrast to the results shown in Scheme 3, a noncompetitive KIE of 1.66 was observed for **1 e**. Furthermore, analysis of the deuterium content of **2 e**–***d***_***1***_ and **2 f**–***d***_***1***_ showed that only 40 and 43 %, respectively, of the deuterium had been transferred to Cβ, with the remainder found at Cα. We also carried out crossover experiments with these allylic alcohols with terminal double bonds, and we found that deuterium scrambling did not take place with these substrates either (see the Supporting Information, Scheme S3). When the reaction was carried out with a non‐deuterated substrate (**1 e**) in D_2_O instead of H_2_O, the product was obtained with 84 % deuterium content at Cα, and no D was found at Cβ.

Moreover, when the isomerization of **1 e**–***d***_***1***_ was carried out in D_2_O, **2 e‐**‐***d***_***2***_ was formed with 40 % deuterium content at Cβ (coming from **1 e**–***d***_***1***_), and 74 % deuterium content at Cα. This means that 20 % of the deuterium at this carbon comes from D_2_O.

### Experimental mechanistic investigation of the Ir^III^‐catalyzed isomerization–chlorination of allylic alcohols

**Scheme 5:**
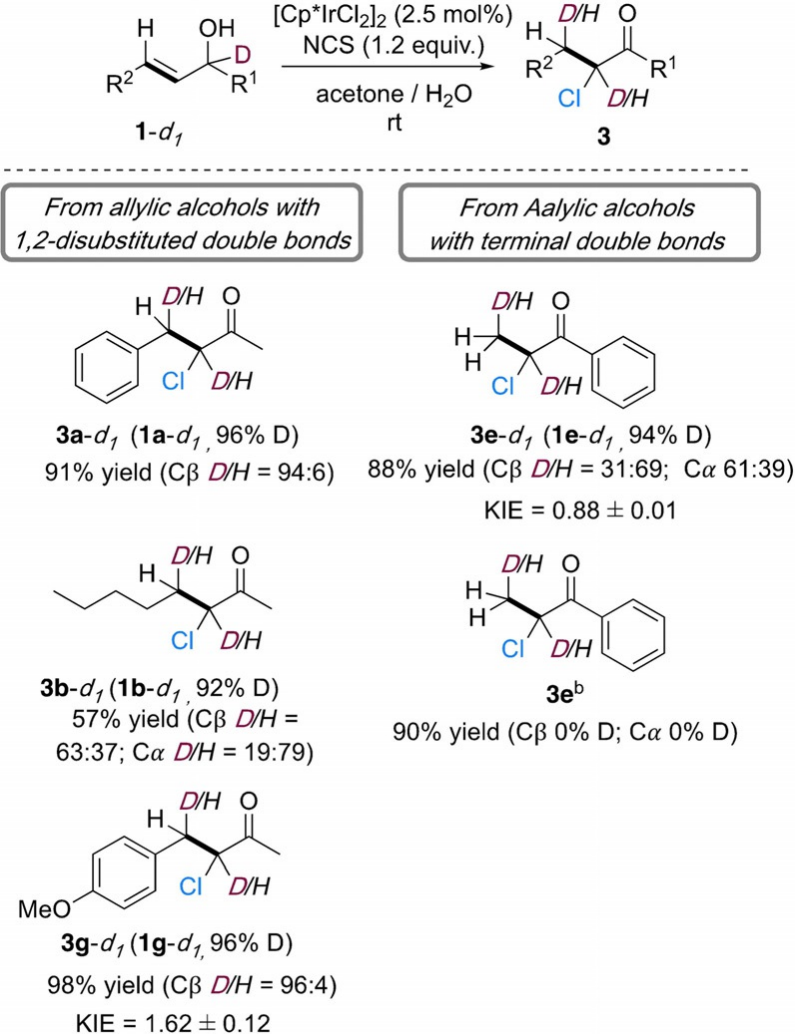
Mechanistic studies for the isomerization–chlorination of allylic alcohols. [a] Reactions were carried out on a 0.1 mmol scale, in acetone/H_2_O (2:1, v/v; 0.1 m), at r.t. for 16 h. Isolated yields. [b] In acetone/D_2_O (2:1, v/v) from **1 e**.

The mechanistic studies reported previously in the group for the isomerization–chlorination of allylic alcohols were carried out on 1,2‐disubstituted alkenes.^[9b]^ Thus, the isomerization of **1 a**–***d***_***1***_ resulted in 96 % of D being transferred exclusively to Cβ. No deuterium scrambling was observed when a crossover experiment was carried out (see Supporting Information, Scheme S3). These studies have now been expanded by running noncompetitive KIEs for the isomerization–chlorination of **1 g** and **1 e**. KIE values of 1.62 and 0.88, respectively, were obtained for these substrates (Scheme 5). When the THF/H_2_O (1:2, v/v) solvent mixture was replaced by acetone/H_2_O (2:1, v/v), allylic alcohols with 1,2‐disubstituted double bonds such as **1 a**–***d***_***1***_, **1 b**–***d***_***1***_, and **1 g**–***d***_***1***_ gave the products also with deuterium exclusively or mainly at Cβ (see Supporting Information). In contrast, when **1 e**–***d***_***1***_ was used in acetone/H_2_O (2:1, v/v), 61 % of the deuterium was transferred to Cα and 31 % to Cβ. Furthermore, when the reaction was carried out using non‐deuterated **1 e** in acetone/D_2_O instead of acetone/H_2_O, no deuterium was detected in the product **3 e**.

### Phosphoric acid as additive in the isomerization and isomerization–chlorination of allylic alcohols

**Scheme 6:**
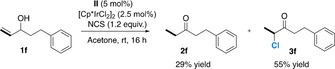
Isomerization–chlorination of **1 f**. [a] Reactions were carried out on a 0.1 mmol scale in 0.1 m. [b] Determined by ^1^H NMR spectroscopy using an internal standard (1,2,4,5‐tetrachloro‐3‐nitrobenzene).

In our previous paper,^[10]^ we reported that a higher temperature (60 °C) was needed to isomerize allylic alcohols when acetone was used as the solvent in the absence of significant amounts of water. We have now carried out the reactions in acetone as the solvent in the presence of 5 mol % of different phosphoric acids. Under these conditions, the isomerization of **1 e** took place at ambient temperature, giving **3 e** in good yields (Table 1, entries 1–4). The isomerization–chlorination of **1 f** in the absence of H_2_O also proceeded in high yield when an acid was added to the reaction mixture. However, and in contrast to the reaction in H_2_O, a mixture of α‐chlorinated ketone **3 f** and saturated ketone **2 f** was obtained (Scheme 6).

**Table 1:** Isomerization of allylic alcohols.^[a]^

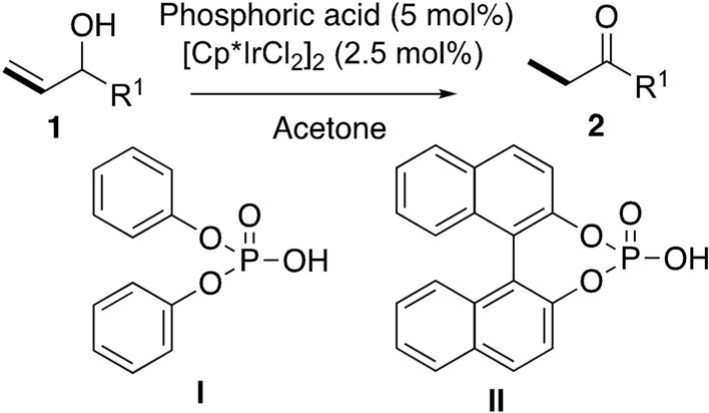				
Entry	**1/**R^1^	*T* [°C]	Acid	**2** [%]^[b]^
1	**1 e/**C_6_H_5_	rt	–	0
2	**1 e/**C_6_H_5_	60	–	67
3	**1 e/**C_6_H_5_	rt	**I**	60
4	**1 e/**C_6_H_5_	rt	**II**	65
5	**1 f/**CH_2_CH_2_C_6_H_5_	rt	**II**	99

[a] All experiments were carried out under an atmosphere of air on a scale of 0.1 mmol of **1** (0.1 m) for 16 h at the temperature indicated. [b] Determined by ^1^H NMR spectroscopy using an internal standard (1,2,4,5‐tetrachloro‐3‐nitrobenzene).

### Computational mechanistic investigations

**Scheme 7:**
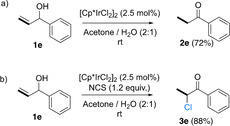
Experimental isomerization and isomerization–chlorination of **1 e**, as studied computationally in this work. [a] Reactions were carried out on a 0.1 mmol scale in 0.1 M. [b] Determined by ^1^H NMR spectroscopy using an internal standard (1,2,4,5‐tetrachloro‐3‐nitrobenzene).

We carried out DFT calculations to study the Ir‐catalyzed isomerization and isomerization–chlorination reactions of allylic alcohols. We will discuss here the results concerning allylic alcohol **1 e** (Scheme 7 a). We also studied the reaction of an allylic alcohol bearing a 1,2‐disubstituted double bond (**1 b**). These results will be briefly mentioned and compared, and the details are given in the Supporting Information. The reaction was modeled in acetone/H_2_O (2:1, v/v) as the solvent. For substrate **1 e**, we also considered the reaction in pure acetone. The results in acetone will be only briefly mentioned here, and the details can be found in the Supporting Information.

We started by calculating the dissociation energy of the initial [Cp^*^IrCl_2_]_2_ complex. The calculations show that the monomer **Int0**, with a Y‐shape structure and a Cl‐Ir‐Cl bond angle of 91.7°, is 3.2 kcal mol^−1^ lower in energy than the dimer. We considered the binding of a water or acetone solvent molecule to **Int0**, but the complexes were found to be considerably higher in energy, by 7.2 and 9.0 kcal mol^−1^, respectively (see the Supporting Information).

**Scheme 8:**
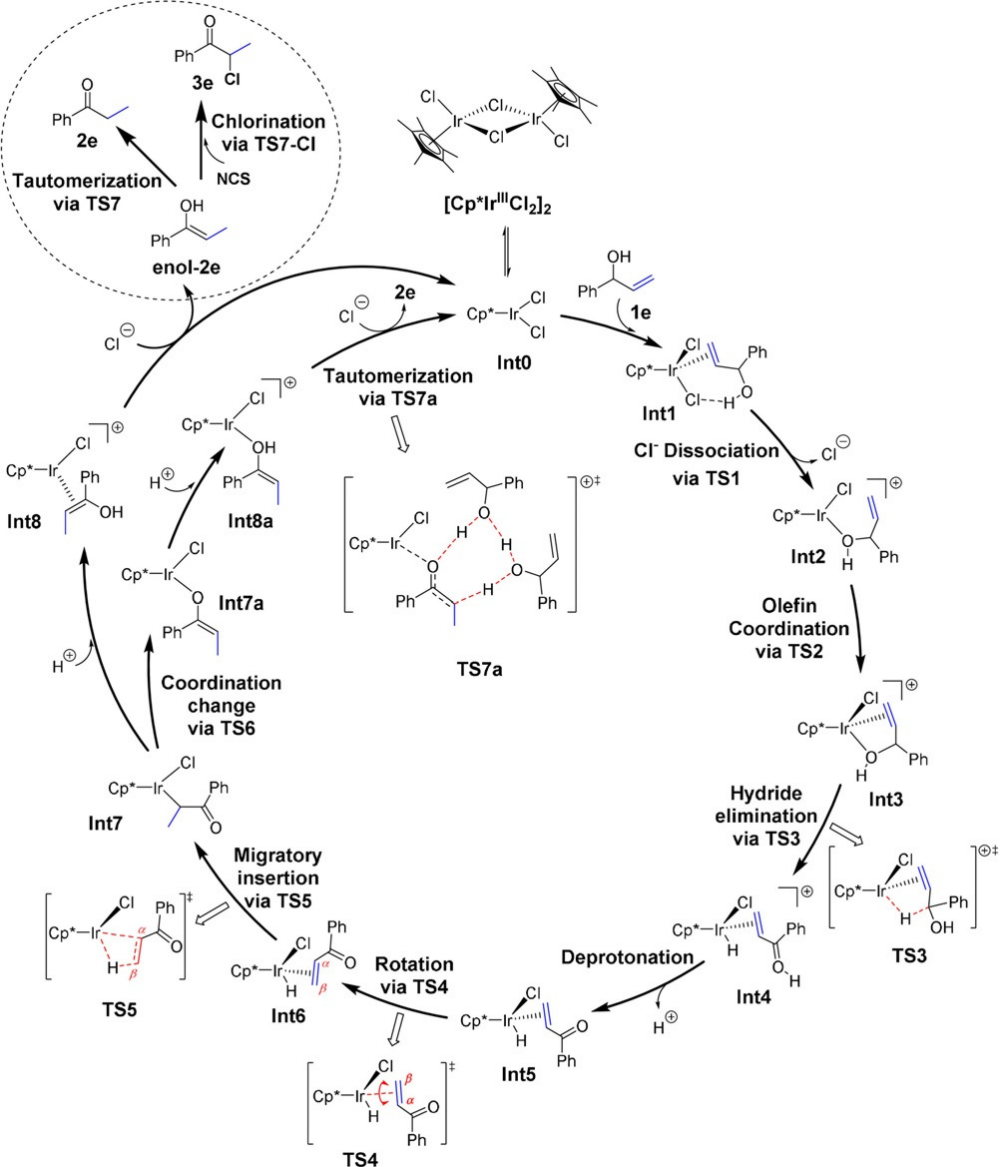
Catalytic cycle for the Ir‐catalyzed isomerization and isomerization–chlorination of allylic alcohol **1 e**.

**Figure 1:**
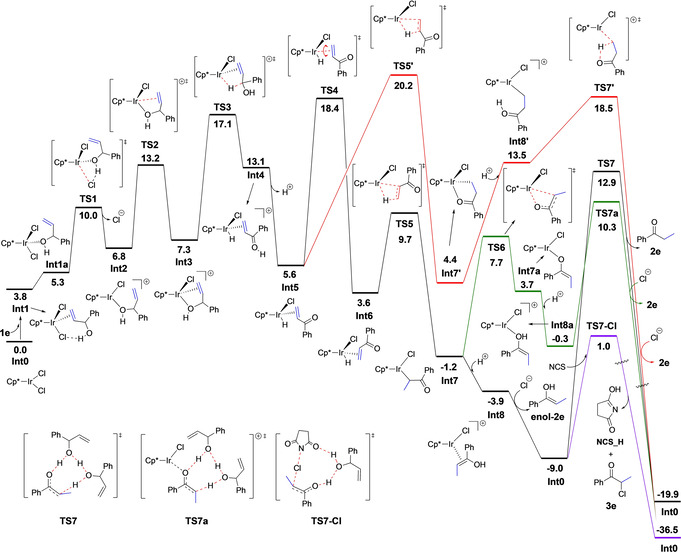
Calculated Gibbs energy profile (kcal mol^−1^) for the Ir‐catalyzed isomerization and isomerization–chlorination reactions of allylic alcohol **1 e** in mixed solvent.

**Figure 2:**
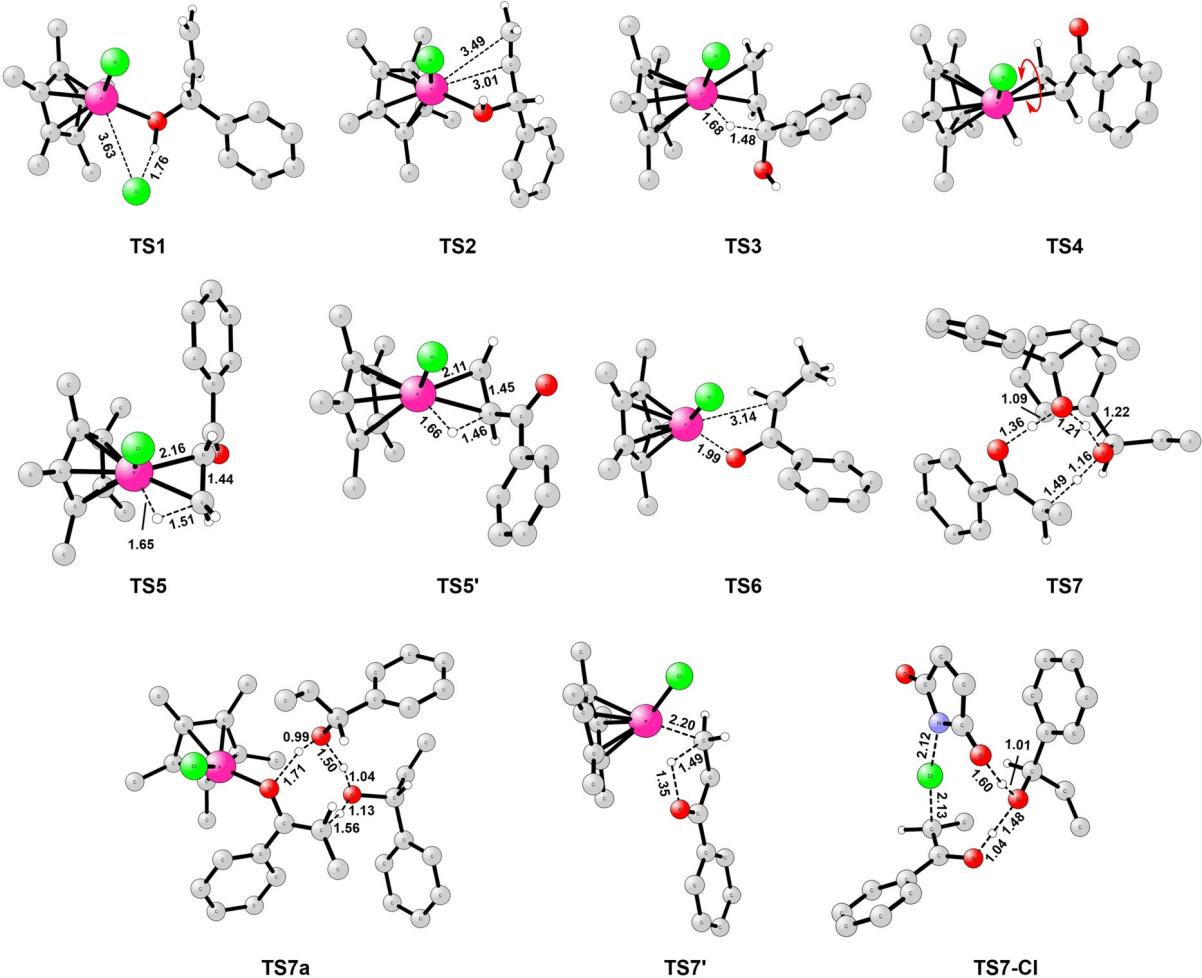
Optimized transition‐state structures for the Ir‐catalyzed isomerization and isomerization–chlorination of allylic alcohol **1 e** in mixed solvent. Note that most hydrogen atoms are omitted for clarity. Pink, green, red, gray, and white colors represent Ir, Cl, O, C, and H, respectively.

Starting from **Int0**, the reaction mechanism that emerges from the calculations is summarized in Scheme 8. The corresponding calculated energy profile is shown in Figure 1, and the optimized structures of the transition states (TSs) involved are shown in Figure 2.

The first step is the coordination of substrate **1 e** to the Ir^III^ complex **Int0**. This can take place either through the C=C double bond to give η^2^‐olefin complex **Int1**, or through the hydroxy group to give **Int1 a**.^[11]^ The two complexes are calculated to be higher in energy than **Int0** by 3.8 and 5.3 kcal mol^−1^, respectively. Although **Int1** is the lower in energy of these two, the next step, the dissociation of chloride via **TS1**, takes place preferentially from **Int1 a**. The calculated barrier is 10.0 kcal mol^−1^ relative to **Int0**, compared to a barrier of 15.5 kcal mol^−1^ for the pathway via **Int1**.

From the resulting cationic intermediate **Int2**, which is +6.8 kcal mol^−1^ relative to **Int0**, the olefin group then coordinates to the iridium via **TS2** (barrier: 13.2 kcal mol^−1^) to give **Int3**, in which the allylic alcohol is coordinated in an η^2^‐fashion through the oxygen and the double bond. This complex then undergoes a hydride elimination through **TS3** to form hydride complex **Int4**. The barrier is calculated to be 17.1 kcal mol^−1^ relative to **Int0**. We considered the possibility of a β‐hydride elimination directly from **Int2**, as well as from the Ir alkoxide after deprotonation of **Int2**, but the barrier was found to be more than 13 kcal mol^−1^ higher than **TS3**, and this scenario can thus be ruled out (see Supporting Information for details). Next, **Int4** liberates a proton to the solvent to generate a more stable neutral intermediate **Int5**, which is 7.5 kcal mol^−1^ lower in energy than **Int4**.

From **Int5**, migratory insertion (MI) can take place, placing the hydrogen atom at either Cα or at Cβ. The reaction placing the H at Cα proceeds directly through **TS5’** with a barrier of 20.2 kcal mol^−1^ relative to **Int0**. However, for the olefin to undergo MI with the H being placed at Cβ, a rotation of the η^2^‐bound C=C double bond via **TS4** must take place, with a barrier of 18.4 kcal mol^−1^. MI from the resulting **Int6** is then very easy, with a barrier of only 6.1 kcal mol^−1^. The energy difference between the two pathways (**TS4–TS5’**) is thus rather small, 1.8 kcal mol^−1^, so both pathways may be operating. This is consistent with the fact that deuterium is found at both Cα and Cβ in the ketones formed from deuterated allylic alcohols bearing terminal double bonds (vide supra, Scheme 4).

The relatively high barrier for the rotation (**Int5**→**TS4**) indicates a strong coordination of the C=C double bond to the iridium. A similar rotation step was observed for the Ru‐catalyzed isomerization of allylic alcohols.^[12]^ The TS for the complete decoordination of the double bond from iridium was calculated to be rather high, 3.1 kcal mol^−1^ more than **TS4**. The strong coordination between the Ir center and the C=C double is consistent with the experimental result that no scrambling of deuterium between similar substrates was observed in the crossover experiments (see above, and Scheme S3).

Following the migratory insertion, the resulting intermediates **Int7** or **Int7’** can accept a proton from solution, for an overall protodemetalation. The reaction can then follow different pathways depending on the intermediate, as shown in Figure 1. In the case of **Int7**, protonation of the oxygen results in **Int8**, which is 2.7 kcal mol^−1^ more stable than **Int7**. From there, a chloride ion can coordinate to the iridium, releasing the enol form of the product (**enol**‐**2 e**) and regenerating the catalyst (**Int0**). This step is calculated to be exergonic by 5.1 kcal mol^−1^, and the energy of **enol‐2 e** is 9.0 kcal mol^−1^ lower than that of the substrate **1 e**. Tautomerization of the enol into ketone **2 e** can then take place outside the catalytic cycle, with a barrier of 21.9 kcal mol^−1^. Ketone **2 e** is 10.9 kcal mol^−1^ more stable than **enol**‐**2 e**. The lowest energy TS that could lead to that tautomerization, **TS7**, was found to involve two molecules of allylic alcohol **1 e** to shuttle the proton from the oxygen to the carbon. We also optimized the same TS with two water molecules instead, but in this case the barrier was found to be 6.3 kcal mol^−1^ higher than **TS7**. We also considered whether the tautomerization could take place at the Ir center before the release of the enol, i.e., at **Int8**, but the barrier for this process was calculated to be more than 20 kcal mol^−1^ higher than **TS7**.

An alternative pathway to form the final product from **Int7** proceeds via **TS6**, which involves a change of coordination mode, namely from η^1^‐*C*‐bound (**Int7**) to η^1^‐*O*‐bound (**Int7 a**) iridium enolate. The barrier for this transformation is calculated to be 8.9 kcal mol^−1^, and the resulting **Int7 a** is 4.9 kcal mol^−1^ higher in energy than **Int7**. From **Int7 a**, protonation at the oxygen yields **Int8 a**, which is found to be 4.0 kcal mol^−1^ lower in energy than **Int7 a**. Interestingly, although the energies of **Int7 a** and **Int8 a** are higher than the corresponding intermediates before the coordination change (**Int7** and **Int8**), the barrier for the subsequent Ir‐catalyzed tautomerization step via **TS7 a** is found to be lower than for **TS7** by 2.6 kcal mol^−1^ (see Figure 1). These results show that this possibility is indeed a viable option, i.e., the tautomerization can take place either using the Ir center or outside the catalytic cycle.

Returning to intermediate **Int7’**, calculations show that the protonation of this species is associated with higher energies, as **Int8’** is found to be 9.1 kcal mol^−1^ higher than **Int7’**. From **Int8’**, an intramolecular proton transfer (**TS7’**) can take place to give the final ketone product. The barrier relative to **Int7’** is calculated to be 14.1 kcal mol^−1^ (18.5 kcal mol^−1^ relative to **Int0**), which is quite feasible. However, there are some experimental results that indicate this mechanism is not operating. When the reaction was run in D_2_O as a cosolvent, no deuterium was found at the Cβ position of the final product (Scheme 4), which shows that this pathway cannot be productive. Another important experimental fact stems from the chlorination reaction. Namely, if the pathway from **Int7’** via **TS7’** was productive, we would expect to observe ketone **2 e** in the product mixture. However, this is not the case, and only the chlorinated product was observed, as discussed above. These two experimental facts show that the barrier for the process from **Int7’** via **TS7’** is probably underestimated in the calculations. This could be a consequence of the way the protonation step is modeled in the calculations, which is associated with considerable errors. In fact, for the other substrate considered in this work, the allylic alcohol with 1,2‐disubstituted double bond **1 b**, the energy barrier for the corresponding step was calculated to be significantly higher (see Supporting Information).

Next we considered the chlorination part (Scheme 7 b). The experimental results with deuterated allylic alcohols (Scheme 5) indicate that the distribution of deuterium between Cα and Cβ is essentially the same for a given substrate in the simple isomerization (Scheme 7 a) as in the isomerization–chlorination (Scheme 7 b). Thus, the chlorination step should happen after the migratory insertion. The calculations show that this is indeed the case, and that the reaction takes place outside the catalytic cycle. This explains why the α‐chlorinated ketone is obtained with complete selectivity and the β‐chlorination has not been observed experimentally. It also explains the regioselectivity of the reaction from aliphatic allylic alcohols, as the non‐chlorinated ketone is not an intermediate on the way to the α‐chlorocarbonyl product. This sets which of the two Cα in the final α‐chlorocarbonyl holds the Cl substituent. As shown in Figure 1, the barrier for chlorination via **TS7**‐**Cl**, involving the assistance of one substrate molecule, is only 10.0 kcal mol^−1^ relative to the enol (**enol**‐**2 e**). The same TS was also calculated with one water molecule instead of the substrate, and the barrier was found to be 0.4 kcal mol^−1^ higher than **TS7**‐**Cl**. Thus, from **enol**‐**2 e**, the barrier for the chlorination **TS7**‐**Cl** is much lower than the barrier for the tautomerization **TS7**, by ca. 12 kcal mol^−1^. This explains why no ketone product was observed in the presence of the chlorinating agent.

To summarize the results of the calculations on substrate **1 e**, the proposed mechanism shown in Scheme 8 has a reasonable overall energy barrier that is consistent with the overall experimental reaction time and temperature. The calculations are not conclusive about the nature of the rate‐determining step (RDS) in the case of the isomerization reaction. The barriers for the hydride elimination (**TS3**), rotation (**TS4**), and tautomerization steps (**TS7** or **TS7 a**) are all quite close in energy, certainly within the error of the methods used. However, from the experiments, we know that the KIE values measured for this reaction are quite small, 1.0–1.7 (see Schemes 3 and 4), which means that the hydride elimination (i.e., oxidation of the allylic alcohol) is not likely to be the RDS. The small KIE values are more consistent with either the rotation or the tautomerization steps being the RDS, since they do not involve the C−H bond directly.

In the case of the isomerization–chlorination reaction, on the other hand, there is no tautomerization, and the barrier for the chlorination step is rather low. This indicates that the rotation step **TS4** is the RDS.

Above (Scheme 4) show an almost equal distribution of deuterium between the Cα and Cβ positions. However, as seen from the energy profiles in Figure 1, the barrier for the direct migratory insertion via **TS5’** is 1.8 kcal mol^−1^ higher than the rotation barrier, **TS4**, which means that much more deuterium should be observed on Cβ than on Cα. Another inconsistency with the experiments is the fact that **TS5’** is slightly higher in energy than **TS7’**, which would mean that the product with deuterium on the Cβ position should be observed when the reaction is run in deuterated water. However, as discussed above, this is not the case experimentally.

These two results show that the energy of the direct migratory insertion **TS5’** is likely to be somewhat overestimated in the calculations, while the energy of **TS7’** is likely to be somewhat underestimated. Namely, a slightly lower energy of **TS5’** and a slightly higher energy of **TS7’** would explain both the deuterium distribution in the products from deuterated allylic alcohols and the reaction outcome in deuterated water. From deuterated allylic alcohols, a slightly higher ratio of deuterium was observed on Cα than on Cβ (see above, Scheme 5), thus the direct MI via **TS5’** to generate the Cα deuterated product should have a slightly lower barrier than the rotation (**TS4**) to generate the Cβ deuterated product.

For the reaction in acetone/D_2_O (2:1), no deuterium was found at the Cβ position of the final product (see above, Scheme 5), and thus the energy of **TS7’** should be higher than **TS5’** to block the pathway **Int7’**→**Int8’**→**TS7’**→**2 e**. In this scenario, the MI step via **TS5’** would become reversible, which would be consistent with the experimental deuterium distribution results, as deuterium was observed on both the Cα and the Cβ.

Very interestingly, this is exactly what was found for the other substrate considered computationally in this work, the 1,2‐disubstituted allylic alcohol **1 b** (see Supporting Information for detailed results). For this compound, the calculations show that the barrier for the intramolecular proton‐transfer step via **1 b**‐**TS6’** is 2.4 kcal mol^−1^ higher in energy than the migratory insertion via **1 b**‐**TS4’** (25.0 vs. 22.6 kcal mol^−1^), which means that the MI step is reversible. Moreover, the energy of the rotation transition state **1 b**‐**TS3** is only 0.9 kcal mol^−1^ lower than that of the MI, compared to 1.8 kcal mol^−1^ in the case of substrate **1 e**. These results show thus that the small inconsistencies in the energies of the reaction of **1 e** compared to the experiments are indeed within the expected accuracy of the computational methods.

Finally, it is important to mention that in this work we have also investigated the reaction of substrate **1 e** in pure acetone solvent. Details are given in the Supporting Information. The calculations show that the reaction mechanism in acetone is quite similar to that in the mixed solvent. The main difference is that in the absence of water, the chloride ion acts as the base, accepting the proton to generate HCl, which later acts as a proton source in the following step. This, together with the lower polarizability of acetone compared to the mixed solvent, results in a slightly different energy profile. The overall barriers in acetone are somewhat higher than in the mixed solvent (ca. 3 kcal mol^−1^), which is consistent with the fact that the reaction in acetone required higher temperatures. Furthermore, the calculated energies obtained are quite consistent with the observed deuterium distribution (see Supporting Information for discussion).

The addition of phosphoric acids as additives in the isomerization of allylic alcohol **1 e** in pure acetone proved to be beneficial as the reaction could be run at r.t. (vide supra, Table 1). This fact suggests that the phosphoric acid can promote the tautomerization reaction by opening up a lower‐energy transition state. By running the isomerization–chlorination reaction in the absence of water but in presence of a phosphoric acid, a mixture of the saturated ketone and the α‐chlorinated ketone was obtained (Scheme 6). This is consistent with the energy of the transition state for the chlorination (**TS7**‐**Cl**) being similar to that of the tautomerization step mediated by the phosphoric acid (see Supporting Information).

## Conclusions

In conclusion, we have carried out an in‐depth study of the mechanism of the Ir^III^‐catalyzed isomerization and isomerization–chlorination reactions of allylic alcohols based on experimental and computational investigations. The proposed mechanism involves a hydride elimination from a cationic intermediate (**Int3**) via **TS3**. Then, after a deprotonation, a migratory insertion takes place preferentially at Cβ, which requires a rotation via **TS4**. However, migratory insertion at Cα is also plausible, with an energy difference of 1.8 kcal mol^−1^. This is consistent with the experimental deuterium distribution in the reactions of deuterated allylic alcohols (Scheme 4). After the migratory insertion, protonation can take place, with or without a coordination change, via **TS6** to generate **Int8 a** or **Int8**, respectively. From **Int8**, coordination of a chloride ion to the iridium regenerates the catalyst and releases the enol derivative of the product (**enol**‐**2 e**), which is converted to the final ketone **2 e** via **TS7**. In parallel, from **Int8 a**, a tautomerization step using the Ir via **TS7 a** generates the final product **2 e** directly. For the isomerization–chlorination of allylic alcohols, our investigations suggest that the chlorination step takes place outside the cycle via **TS7**‐**Cl**, which has a much lower energy than that of the tautomerization (**TS7**). This explains the complete selectivity observed for this transformation. Finally, the presented results highlight the importance of adopting an interactive experimental–computational approach to reach deep insights into the mechanisms of complex reactions.

## Experimental Section

### Isomerization of allylic alcohols 1

To a solution of the allylic alcohol **1** (0.2 mmol, 1 equiv) in a mixture of acetone and H_2_O (2:1, 0.1 m), [Cp*IrCl_2_]_2_ (4 mg, 2.5 mol %) was added. The resulting mixture was stirred at room temperature and monitored by TLC. When the reaction was completed, EtOAc (10 mL) and H_2_O (10 mL) were added to the mixture and the aqueous layer was extracted with EtOAc (3×10 mL). The combined organic layers were dried over MgSO_4_, filtered and the solvent was removed under reduce pressure. The crude was purified by flash chromatography affording the corresponding carbonyl compound **2**.

### Isomerization/ chlorination of allylic alcohols 1

To a solution of the allylic alcohol **1** (0.2 mmol, 1 equiv) and *N*‐chlorosuccinimide (32 mg, 0.24 mmol, 1.2 equiv) in a mixture of acetone and H_2_O (2:1, 0.1 m), [Cp*IrCl_2_]_2_ (4 mg, 2.5 mol %) was added. The resulting mixture was stirred at room temperature and monitored by TLC. When the reaction was completed, EtOAc (10 mL) and H_2_O (10 mL) were added to the mixture and the aqueous layer was extracted with EtOAc (3×5 mL). The combined organic layers were dried over MgSO_4_, filtered and the solvent was removed under reduce pressure. The crude was purified by flash chromatography affording the corresponding α‐chlorocarbonyl compound **3**.

### Computational details

All DFT calculations were carried out using the Gaussian 09 program^[13]^ with the B3LYP functional,^[14]^ and dispersion effects described by the D3‐BJ method^[15]^ were included in all calculations. Geometry optimizations were carried out in the gas phase, using a mixture of basis sets: LanL2DZ^[16]^ for Ir and 6‐31G(d,p) for the other atoms. Frequency calculations of all the optimized geometries were carried out at the same level of theory to verify their nature as minima (no imaginary frequency) or transition‐state (TS) structures (one imaginary frequency), and to obtain the Gibbs energy corrections at room temperature. Intrinsic reaction coordinate (IRC) calculations were performed for selected structures to confirm the connectivity between TSs and minima.^[17]^ Single‐point energy calculations were carried out with the LanL2TZ basis set for Ir and 6–311+G(2d,2p) for the other atoms. The solvation effects were included using the SMD model.^[18]^ In the case of the mixed solvent acetone/H_2_O (2:1), the dielectric constant of 1,2‐ethanediol (*ϵ*=40.2) was used because it is close to the estimated average of the two solvents. A correction term of {*RT* ln (24.46)=1.9 kcal mol^−1^} was added to the Gibbs energy for each species, except H_2_O, to account for the 1 atm to 1 m standard state change. For H_2_O as a cosolvent, which has a concentration of 18.5 m in the 2:1 acetone/water mixed solvent used in the experiment, a term of {*RT* ln (18.5×24.46)=3.6 kcal mol^−1^} was added to the Gibbs energy. Some of the steps in the mechanism involve the release or uptake of a proton. To estimate the Gibbs energy of H^+^_solv_ in the acetone/water (2:1), we used the following procedure. The values used for the gas‐phase Gibbs energy of a proton and its solvation free energy in water are −265.9 and −6.3 kcal mol^−1^, respectively.^[19]^ The Gibbs energy required to transfer a proton from water to an acetone/water (2:1) mixture is estimated to be −2.7 kcal mol^−1^.^[20]^ Therefore, the value used for the Gibbs energy of H^+^_solv_ in the acetone/water (2:1) mixed solvent was set to {(‐265.9–6.3‐2.7)=−274.9 kcal mol^−1^} in the calculations.

## Conflict of interest

The authors declare no conflict of interest.

## Supporting information

As a service to our authors and readers, this journal provides supporting information supplied by the authors. Such materials are peer reviewed and may be re‐organized for online delivery, but are not copy‐edited or typeset. Technical support issues arising from supporting information (other than missing files) should be addressed to the authors.

Supplementary
