# Supplementary material for: Unraveling the Mechanism of the IrIII‐Catalyzed Regiospecific Synthesis of α‐Chlorocarbonyl Compounds from Allylic Alcohols
Source: Chemistry. 2020 Oct 14;26(65):14978–86. doi: 10.1002/chem.202002845 (PMC7756427; doi:10.1002/chem.202002845)
Supplement: Supplementary file 1 — Supplementary [file CHEM-26-14978-s001.pdf]

Chemistry–A European Journal

Supporting Information

Unraveling the Mechanism of the Ir^{III}-Catalyzed Regiospecific Synthesis of α-Chlorocarbonyl Compounds from Allylic Alcohols

Man Li, Amparo Sanz-Marco, Samuel Martinez-Erro, Víctor García-Vázquez, Binh Khanh Mai, Jacob Fernández-Gallardo, Fahmi Himo,* and Belén Martín-Matute*[a]

Table of contents

**S2**	General Information
**S2**	Synthesis and characterization of allylic alcohols
**S2**	General procedure for the isomerization of allylic alcohols
**S3**	Characterization data for ketones **2e-2f**
**S4**	General procedure for the isomerization / chlorination of allylic alcohols
**S4**	Characterization data for α-chloroketones **3a-3e**
**S5**	Kinetic isotope effect studies
**S8**	Deuterium labeling cross-over studies for the isomerization of allylic alcohols
**S11**	^{1}H and ^{13}C NMR spectra of allylic alcohol **1g**-d_1
**S12**	^{1}H and ^{13}C NMR spectra of ketones **2e-2f**
**S16**	^{1}H and ^{13}C NMR spectra of α-chloroketones **3a-3e**
**S20**	^{1}H and ^{13}C NMR spectra of deuterium labeling cross-over studies
**S24**	DFT studies
**S24**	Reaction profile of the Ir(III) catalyzed isomerization and isomerization-chlorination of allylic alcohol **1b**
**S26**	Reaction profile of the Ir(III) catalyzed isomerization and isomerization-chlorination of allylic alcohol **1e** in acetone
**S28**	Additional results for the Ir(III) catalyzed isomerization and isomerization-chlorination of allylic alcohol **1e** in mixture solvent
**S30**	Calculated absolute energies and energy corrections
**S32**	Cartesian coordinates
**S76**	Reference

General information

All reagents were used as obtained from commercial sources without further purification. Flash chromatography was performed with 60 Å (35-70 μm) silica gel (GC 60A 35-70 Micron, DAVISIL) using mixtures pentane / EtOAc as eluent. Analytical TLC was performed on aluminum plates pre-coated with silica gel (Merck, Silica Gel 60 F254). Compounds were detected by exposure to UV light or by revealing the plates in a solution of 5% $KMnO_4$ in water. ^{1}H and ^{13}C NMR spectra were recorded at 400 or 500 MHz and 100 or 125 MHz respectively on Bruker Advance spectrometers. Chemical shifts (δ) are shown in ppm, using the residual peaks of $CH(D)Cl_3$ (δ_H 7.26 and δ_C 77.00) as reference. Coupling constants (J) are given in Hz. High-resolution mass spectra (HRMS) were recorded on Bruker *micro*TOF ESI-TOF mass spectrometer.

Synthesis and characterization of allylic alcohols

Allylic alcohols **1a**,[1] **1a**-d_1,[1] **1b**,[1] **1b**-d_1,[1] **1e**,[1] **1e**-d_1,[2] **1f**,[1] **1f**-d_1,[3] **1g**[4] were synthesized according to literature procedures.

(*E*)-4-(4-Methoxyphenyl)but-3-en-2-*d*-2-ol (**1g**-d_1)

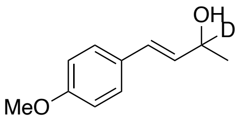

To a stirred solution of (*E*)-4-(4-methoxyphenyl)but-3-en-2-one (10 mmol, 1.76 g) and $CeCl_3$ x $7H_2O$ (10 mmol, 3.65 g) in MeOH (50 mL), $NaBD_4$ (10 mmol, 0.42g) was added at 0 °C. The reaction was stirred until TLC analysis showed no starting material left. After addition of an aqueous solution of NH_4Cl (sat.,10 mL), MeOH was evaporated and the allylic alcohol was extracted with EtOAc (3 x 15 mL), washed with brine, dried over $MgSO_4$, filtered and evaporated. Purification by column chromatography (SiO_2; petroleum ether / EtOAc, 9:1) afforded **1g**-d_1 as a white solid (1.27 g, 71 %, 96% D).

^{1}H NMR (400 MHz, $CDCl_3$) δ 7.31–7.28 (m, 2H), 6.86–6.83 (m, 2H), 6.49 (d, J = 15.9 Hz, 1H), 6.11 (d, J = 15.9 Hz, 1H), 3.70 (s, 3H), 1.35 (s, 3H) ppm.
^{13}C NMR (100 MHz, $CDCl_3$) δ 159.3, 131.5, 129.5, 129.0, 127.7, 114.1, 68,6 (t, $^1J_{C\text{-}D}$ = 21.9 Hz), 55.3, 23.4 ppm.
HRMS (ESI): m/z calcd for $C_{11}H_{13}O_2D+Na^+$: 202.0949 $[M+Na]^+$; found: 202.0952.

General procedure for the isomerization of allylic alcohols

To a solution of the allylic alcohol **1** (0.2 mmol, 1 equiv.) in a mixture of acetone and H_2O (2:1, 0.1 M), $[Cp^*IrCl_2]_2$ (4 mg, 2.5 mol%) was added. The resulting mixture was stirred at room temperature and monitored by TLC. When the reaction was completed, EtOAc (10 mL) and H_2O (10 mL) were added to the mixture and the aqueous layer was extracted with EtOAc (3 x 10 mL). The combined organic layers were dried over $MgSO_4$, filtered and the solvent was removed under reduce pressure. The crude was purified by flash chromatography affording the corresponding carbonyl compound **2**.

Characterization data for ketones 2e-2f

Deuterium-propiophenone (**2e**-d_1)

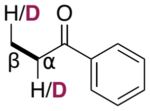

The title compound was prepared according to general procedure from allylic alcohol **1e**-d_1 (94% D) (27 mg). The reaction was stirred 3 h at room temperature. Purification by column chromatography (SiO_2; Petroleum ether / EtOAc = 10:1) afforded **2e**-d_1 (94% D) as a colorless oil (16 mg, 62%). Deuterium content was determined to 40% in Cβ and 54% in Cα.

^{1}H NMR (400 MHz, $CDCl_3$) δ 7.99–7.94 (m, 2H), 7.58–7.53 (m, 1H), 7.49–7.44 (m, 2H), 3.03–2.95 (m, 1.46H), 1.24–1.19 (m, 2.59H) ppm.

^{13}C NMR (100 MHz, $CDCl_3$) δ 201.0, 137.1, 133.0, 128.7, 128.1, 31.9, 31.8 (t, $^1J_{C\text{-}D}$ = 19.2 Hz, product with deuterium in Cα), 8.3, 8.14 (t, $^1J_{C\text{-}D}$ = 19.9 Hz, product with deuterium in Cβ) ppm.

HRMS (ESI): m/z calcd for $C_9H_9OD+Na^+$: 158.0687 $[M+Na]^+$; found: 158.0687.

The title compound was also prepared according to general procedure using acetone and D_2O (2:1, 0.1 M) instead of acetone and H_2O (2:1, 0.1 M) from allylic alcohol **1e** (27 mg). The reaction was stirred 3 h at room temperature. Purification by column chromatography (SiO_2; Petroleum ether / EtOAc = 10:1) afforded **2e**-d_1 (84% D) as a colorless oil (15 mg, 57%). Deuterium content was determined to 84% in Cα.

^{1}H NMR (400 MHz, $CDCl_3$) δ 7.98–7.94 (m, 2H), 7.57–7.54 (m, 1H), 7.48–7.44 (m, 2H), 3.04–2.95 (m, 1.16H), 1.25–1.21 (m, 3H) ppm.

^{13}C NMR (100 MHz, $CDCl_3$) δ 201.1, 137.1, 133.0, 128.7, 128.1, 31.9, 31.6 (t, $^1J_{C\text{-}D}$ = 19.3 Hz, product with deuterium in Cα), 8.3 ppm.

Deuterium-1-phenylpentan-3-one (**2f**-d_1)

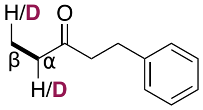

The title compound was prepared according to general procedure from allylic alcohol **1f**-d_1 (99% D) (33 mg). The reaction was stirred 3 h at room temperature. Purification by column chromatography (SiO_2; Petroleum ether / EtOAc = 10:1) afforded **2f**-d_1 (99% D) as a colorless oil (31 mg, 99%). Deuterium content was determined to 43% in Cβ and 56% in Cα.

^{1}H NMR (400 MHz, $CDCl_3$) δ 7.30–7.26 (m, 2H), 7.21–7.17 (m, 3H), 2.92–2.89 (m, 2H), 2.75–2.17 (m, 2H), 2.42–2.37 (m, 1.44H), 1.05–1.01 (m, 2.57H) ppm.

^{13}C NMR (100 MHz, $CDCl_3$, mixture of products with deuterium in Cα or Cβ) δ 210.9, 210.8, 141.3, 128.6, 128.4, 126.2, 44.02, 44.01, 36.2, 35.9 (t, $^1J_{C\text{-}D}$ = 19.2 Hz, product with deuterium in Cα), 29.99, 29.97, 7.83, 7.73 (t, $^1J_{C\text{-}D}$ = 19.9 Hz, product with deuterium in Cβ) ppm.

HRMS (ESI): m/z calcd for $C_{11}H_{13}OD+Na^+$: 186.1000 $[M+Na]^+$; found: 186.0992.

General procedure for the isomerization / chlorination of allylic alcohols

To a solution of the allylic alcohol **1** (0.2 mmol, 1 equiv.) and N-chlorosuccinimide (32 mg, 0.24 mmol, 1.2 equiv.) in a mixture of acetone and H_2O (2:1, 0.1 M), $[Cp^*IrCl_2]_2$ (4 mg, 2.5 mol%) was added. The resulting mixture was stirred at room temperature and monitored by TLC. When the reaction was completed, EtOAc (10 mL) and H_2O (10 mL) were added to the mixture and the aqueous layer was extracted with EtOAc (3 x 5 mL). The combined organic layers were dried over $MgSO_4$, filtered and the solvent was removed under reduce pressure. The crude was purified by flash chromatography affording the corresponding α-chlorocarbonyl compound.

Characterization data for α-chloroketones 3a-3e

4-Deuterium-3-chloro-4-phenylbutan-2-one (3a-d_1)

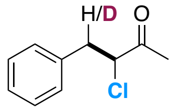

The title compound was prepared according to general procedure from allylic alcohol **1a**-d_1 (96% D) (36 mg). The reaction was stirred 18 h at room temperature. Purification by column chromatography (SiO_2; Petroleum ether / EtOAc = 10:1) afforded **3a**-d_1 (94% D) as a colorless oil (33 mg, 91%). Deuterium content was determined to 94% in Cβ.

^{1}H NMR (400 MHz, $CDCl_3$, mixture of 2 diastereomers (1:1)) δ 7.34–7.21 (m, 5H), 4.40 (d, J = 7.9 Hz, 1H), 3.32 (dt, J = 6.2, 2.1 Hz, 0.53H), 3.07 (dt, J = 8.0, 2.1 Hz, 0.53H), 2.29 (s, 3H), 2.28 (s, 3H) ppm. ^{13}C NMR (100 MHz, $CDCl_3$, mixture of 2 diastereomers (1:1)) δ 202.8, 136.3, 129.5, 128.8, 127.4, 63.88, 63.86, 39.5 (t, $^1J_{C\text{-}D}$ = 19.9 Hz), 39.4 (t, $^1J_{C\text{-}D}$ = 20.4 Hz), 27.0 ppm. ^{1}H and ^{13}C NMR spectras were in agreement with those reported in the literature.[5]

Deuterium-3-chlorooctan-2-one (3b-d_1)

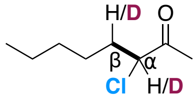

The title compound was prepared according to general procedure from allylic alcohol **1b**-d_1 (92% D) (33 mg). The reaction was stirred 18 h at room temperature. Purification by column chromatography (SiO_2; Petroleum ether / EtOAc = 10:1) afforded **3b**-d_1 (82% D) as a colorless oil (18 mg, 57%). Deuterium content was determined to 63% in Cβ and 19% in Cα.

^{1}H NMR (400 MHz, $CDCl_3$) δ 4.17 (d, J = 8.2 Hz, 0.81H), 2.31 (s, 3H), 1.94–1.78 (m, 1.37H), 1.34–1.30 (m, 6H), 0.91–0.88 (m, 3H) ppm.

^{13}C NMR (100 MHz, $CDCl_3$, mixture of 2 diastereomers (1:1)) δ 204.4, 203.8, 64.4, 64.3 (t, $^1J_{C\text{-}D}$ = 17.2 Hz, product with deuterium in Cα), 33.8, 33.6 (t, $^1J_{C\text{-}D}$ = 20.0 Hz, product with deuterium in Cβ), 33.6 (t, $^1J_{C\text{-}D}$ = 19.4 Hz, product with deuterium in Cβ), 31.24, 31.22, 29.9, 29.8, 26.1, 26.0, 25.8, 25.7, 22.5 ppm.

HRMS (ESI): m/z calcd for $C_8H_{14}OD^{35}Cl+Na^+$: 186.0766 $[M+Na]^+$; found: 186.0762.

4-Deuterium-3-chloro-4-(4-methoxyphenyl)butan-2-one (**3g**-d_1)

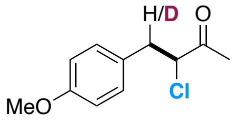

The title compound was prepared according to general procedure from allylic alcohol **1g**-d_1 (96% D) (36 mg). The reaction was stirred 18 h at room temperature. Purification by column chromatography (SiO_2; Petroleum ether / EtOAc = 9:1) afforded **3g**-d_1 (96% D) as a colorless oil (35 mg, 98%). Deuterium content was determined to 96% in Cβ.

^{1}H NMR (400 MHz, $CDCl_3$, mixture of 2 diastereomers (1:1)) δ 7.15–7.11 (m, 2H), 6.87–6.83 (m, 2H), 4.36–4.34 (m, 1H), 3.79 (s, 3H), 3.26–3.24 (m, 0.52H), 3.03–3.01 (m, 0.52H), 2.27 (s, 3H), 2.27 (s, 3H) ppm.

^{13}C NMR (100 MHz, $CDCl_3$, mixture of 2 diastereomers (1:1)) δ 203.0, 158.9, 130.5, 128.2, 114.2, 64.09, 64.07, 55.4, 39.2, 38.88 (t, $^1J_{C\text{-}D}$ = 20 Hz), 38.85 (t, $^1J_{C\text{-}D}$ = 20.0 Hz), 27.02, 27.01 ppm.

HRMS (ESI): m/z calcd for $C_{11}H_{12}O^{35}ClD+Na^+$: 236.0559 $[M+Na]^+$; found: 236.0554.

Deuterium-2-chloro-1-phenylpropan-1-one (**3e**-d_1)

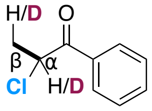

The title compound was prepared according to general procedure from allylic alcohol **1e**-d_1 (94% D) (34 mg). The reaction was stirred 18 h at room temperature. Purification by column chromatography (SiO_2; Petroleum ether / EtOAc = 10:1) afforded **3e**-d_1 (92% D) as a colorless oil (30 mg, 88%). Deuterium content was determined to 31% in Cβ and 61% in Cα.

^{1}H NMR (400 MHz, $CDCl_3$) δ 8.06–8.03 (m, 2H), 7.65–7.61 (m, 1H), 7.54–7.50 (m, 2H), 5.29–5.26 (m, 0.39H), 1.77–1.75 (m, 2.69H) ppm.

^{13}C NMR (100 MHz, $CDCl_3$) δ 193.8, 134.3, 133.9, 129.1, 128.9, 52.9, 52.6 (t, $^1J_{C\text{-}D}$ = 23 Hz, product with deuterium in Cα), 20.0, 19.9 (t, $^1J_{C\text{-}D}$ = 20.0 Hz, product with deuterium in Cβ) ppm.

Kinetic isotope effect studies

Isomerization

*[**1e**-d_1] and [**1e**]*

Two parallel reactions, one with 1-phenylprop-2-en-1-ol (**1e**) and another with 1-phenylprop-2-en-1-*d*-1-ol (94% D, **1e**-d_1), were carried out. Allylic alcohol **1e** or **1e**-d_1 (0.1 mmol) and $[Cp^*IrCl_2]_2$ (2.5 mol%, 2 mg) were dissolved in Acetone-d_6 / D_2O (2:1) (0.1 M, 1.0 mL) in a capped vial. The solution was transferred into an NMR tube and the tube was transferred to the NMR spectrometer. Signals from the aromatic protons of the product were used to monitor the formation of propiophenone (**2e**). ^{1}H NMR spectra were recorded every 3 min. Each experiment was performed by duplicate. The average of the initial rate plots for the experiments with each allylic alcohol (**1e**, **1e**-d_1) are given in Figure S1. A KIE of 1.66 ± 0.11 was obtained.

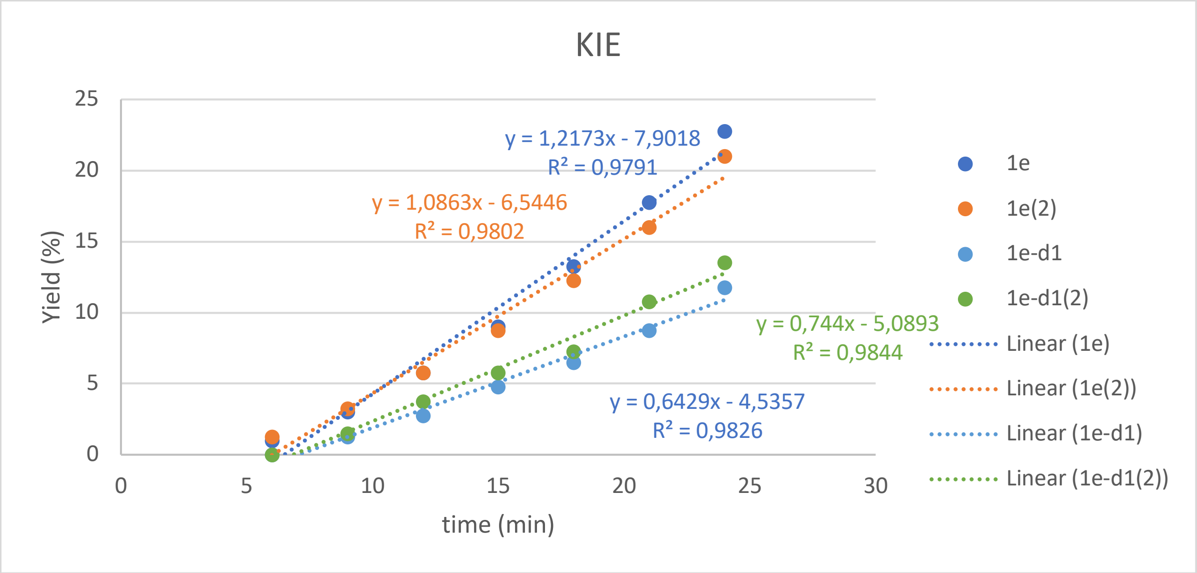

Figure S1. Kinetic isotope effect of the Ir(III) catalyzed isomerization of **1e**.

Isomerization / chlorination

*[**1e**-d_1] and [**1e**]*

Ten parallel reactions, five with 1-phenylprop-2-en-1-ol (**1e**) and other five with 1-phenylprop-2-en-1-*d*-1-ol (94% D, **1e**-d_1), were carried out. Allylic alcohol **1e** or **1e**-d_1 (0.1 mmol), NCS (16 mg, 0.12 mmol) and $[Cp^*IrCl_2]_2$ (2.5 mol%, 2 mg) were dissolved in Acetone / H_2O (2:1) (0.1 M, 1.0 mL) in a capped vial. The reactions were quenched at 30 seconds, 1 min, 1,30 min, 2 min and 2,30 min. Signals from the aromatic protons of the product were used to monitor the formation of 2-chloro-1-phenylpropan-1-one (**3e**). Each experiment was performed by duplicate. The average of the initial rate plots for the experiments with each allylic alcohol (**1e**, **1e-d_1**) are given in Figure S2. A KIE of 0.88 ± 0.01 was obtained.

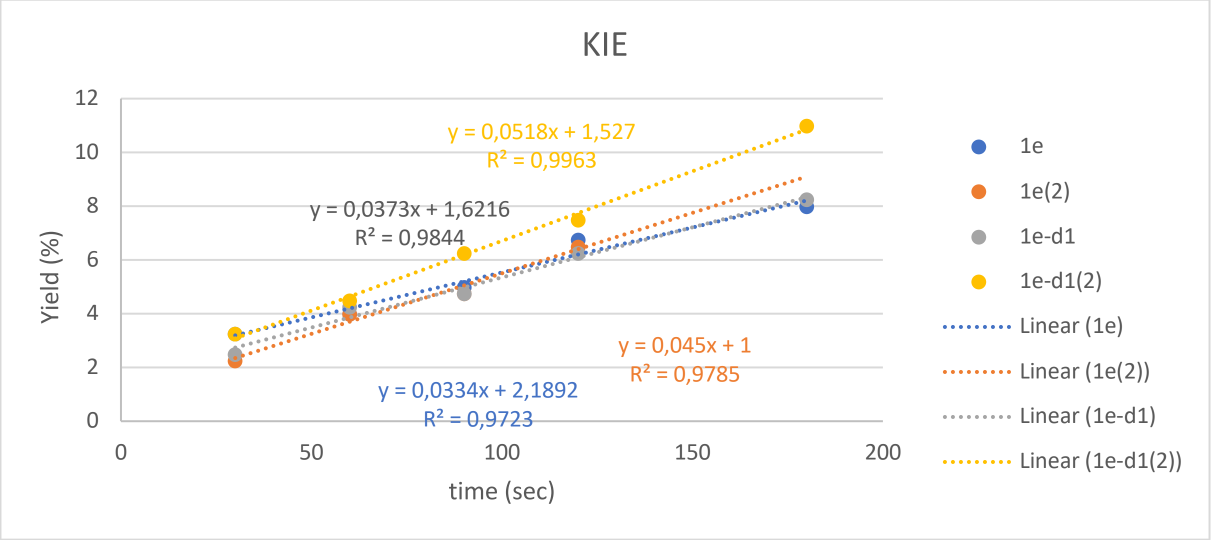

Figure S2. Kinetic isotope effect of the Ir(III) catalyzed isomerization / chlorination of **1e.**

Kinetic profile:

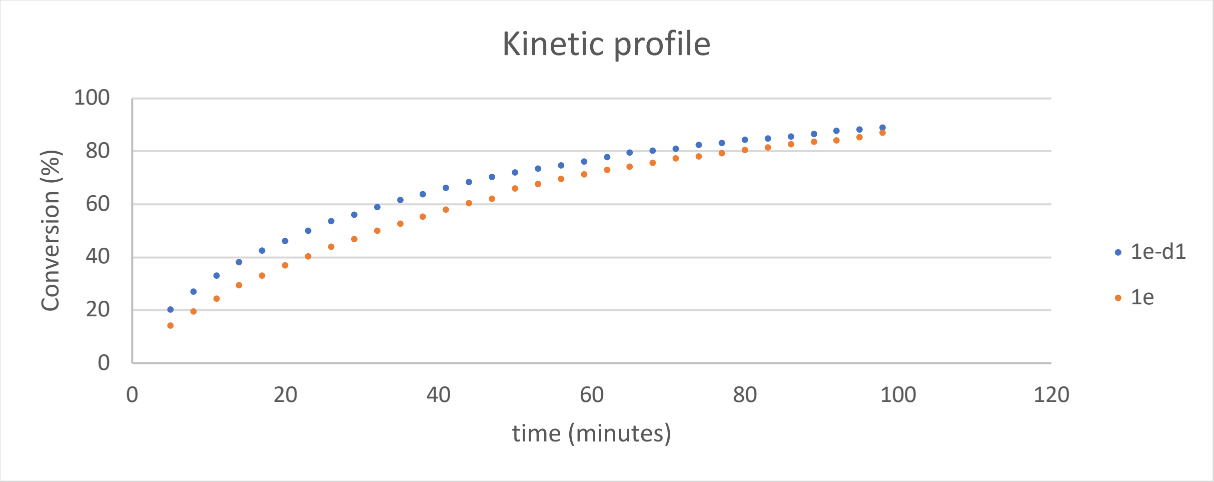

Figure S3. Kinetic profile of the Ir(III) catalyzed isomerization / chlorination of **1e.**

*[**1g**-d_1] and [**1g**]*

Two parallel reactions, one with (*E*)-4-(4-methoxyphenyl)but-3-en-2-ol (**1g**) and another with (*E*)-4-(4-methoxyphenyl)but-3-en-2-*d*-2-ol (96% D, **1g-*d*$_1$**), were carried out. Allylic alcohol **1g** or **1g-*d*$_1$** (0.1 mmol) and [Cp*IrCl$_2$]$_2$ (2.5 mol%, 2 mg) were dissolved in Acetone-*d*$_6$ / D_2O (2:1) (0.1 M, 1.0 mL) in a capped vial. The solution was transferred into an NMR tube and the tube was transferred to the NMR spectrometer. Signals from the aromatic protons of the product were used to monitor the formation of propiophenone (**2g**). ^{1}H NMR spectra were recorded every 3 min. Each experiment was performed by duplicate. The average of the initial rate plots for the experiments with each allylic alcohol (**1g**, **1g-*d*$_1$**) are given in Figure S4. A KIE of 1.62 ± 0.12 was obtained.

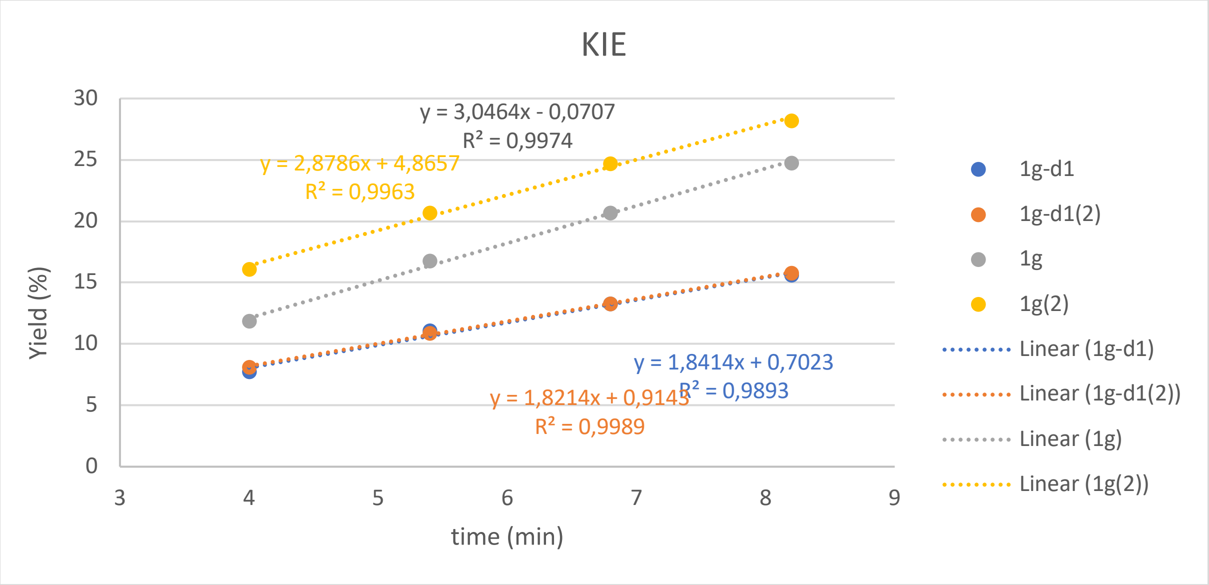

Figure S4. Kinetic isotope effect of the Ir(III) catalyzed isomerization / chlorination of **1g.**

Deuterium labeling cross-over studies for the isomerization of allylic alcohols

Deuterium labeling cross-over studies for the isomerization of allylic alcohols with 1,2-disubstituted double bonds (Scheme S1) were described in our previous work.[1]

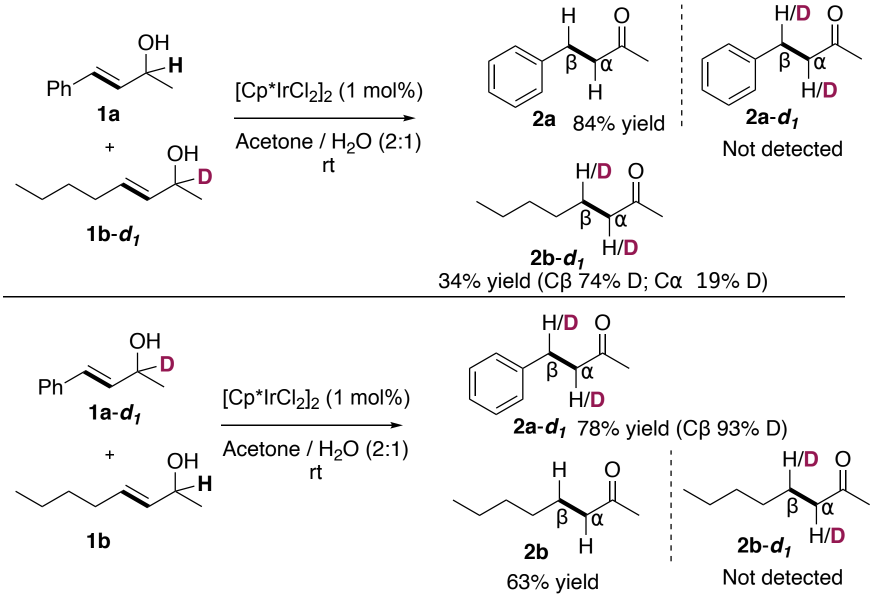

Scheme S1. Cross-over experiments previously reported

*[**1a**-d_1] and [**1g**]*

Allylic alcohols [**1a**-d_1] (0.1 mmol, 15 mg, 96%D) and [**1g**] (0.1 mmol, 18 mg) were dissolved in the same flask in a mixture of acetone and water (2:1, 0.1 M). $[Cp^*IrCl_2]_2$ (4.0 mg, 2.5 mol%) was added, the reaction was stirred for 3 h until TLC indicated no starting material was remaining. When the reaction was completed, EtOAc (10 mL) and H_2O (10 mL) were added to the mixture and the aqueous layer was extracted with EtOAc (3 x 10 mL). The combined organic layers were dried over $MgSO_4$, filtered and the solvent was removed under reduced pressure. Purification by column chromatography (SiO_2; Petroleum ether / EtOAc 10:1) afforded **2a**-*d1* as a colorless oil (13 mg, 84%) and **2g** as a white solid (15 mg, 86%). *No deuterium scrambling between the two substrates was observed.*

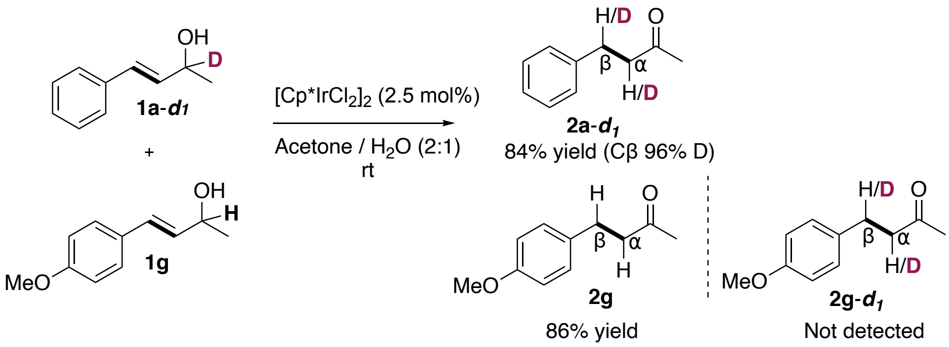

Scheme S2. Cross-over experiments of **1g** and **1a**-d_1

4-Deuterium-4-phenylbutan-2-one (**2a**-d_1)

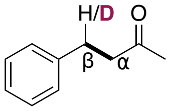

Deuterium content was determined to 96% in Cβ.
^{1}H NMR (400 MHz, $CDCl_3$) δ 7.30–7.26 (m, 2H), 7.21–7.17 (m, 3H), 2.91–2.86 (m, 1.04H), 2.78–2.75 (m, 2H), 2.14 (s, 3H) ppm.
^{13}C NMR (100 MHz, $CDCl_3$) δ 208.1, 141.1, 128.7, 128.4, 126.3, 45.3, 30.2, 29.6 (t, $^1J_{C-D}$ = 19.7 Hz,) ppm. ^{1}H and ^{13}C NMR spectras were in agreement with those reported in the literature.[1]

4-(4-Methoxyphenyl)butan-2-one (**2g**)

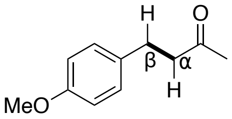

^{1}H NMR (400 MHz, $CDCl_3$) δ 7.11–7.08 (m, 2H), 6.84–6.81 (m, 2H), 3.78 (s, 3H), 2.86–2.82 (m, 2H), 2.74–2.70 (m, 2H), 2.13 (s, 3H) ppm.
^{13}C NMR (100 MHz, $CDCl_3$) δ 208.3, 158.1, 133.2, 129.4, 114.0, 55.4, 45.6, 30.2, 29.0 ppm.
HRMS (ESI): m/z calcd for $C_{11}H_{14}O_2+Na^+$: 201.0886 [M+Na]$^+$; found: 201.0881.

*[**1e**-d_1] and [**1h**]*

Allylic alcohols [**1e**-d_1] (0.1 mmol, 14 mg, 94%D) and [**1g**] (0.1 mmol, 17 mg) were dissolved in the same flask in a mixture of acetone and water (2:1, 0.1 M). $[Cp^*IrCl_2]_2$ (4.0 mg, 2.5 mol%) was added, the reaction was stirred for 3 h until TLC indicated no starting material was remaining. When the reaction was completed, EtOAc (10 mL) and H_2O (10 mL) were added to the mixture and the aqueous layer was extracted with EtOAc (3 x 5 mL). The combined organic layers were dried over $MgSO_4$, filtered and the solvent was removed under reduced pressure. Purification by column chromatography (SiO_2; Petroleum ether / EtOAc 10:1) afforded **2e**-d_1 as a colorless oil (7 mg, 50%) and **2h** as a white solid (12 mg, 73%). *No deuterium scrambling between the two substrates was observed.*

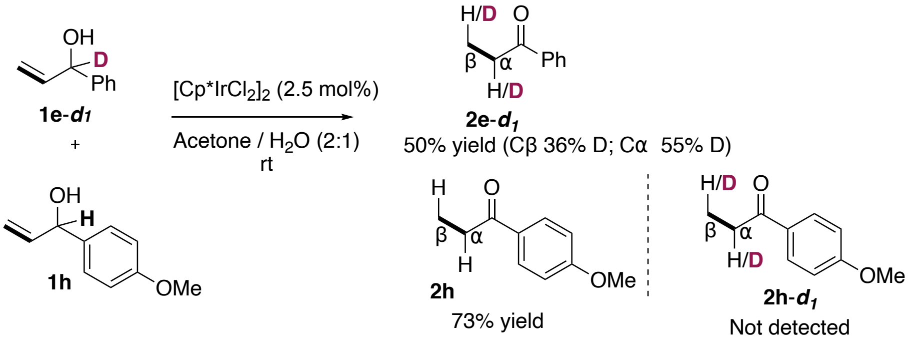

Scheme S3. Cross-over experiments of **1h** and **1e**-d_1

1-(4-Methoxyphenyl)propan-1-one (2h)

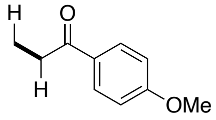

^{1}H NMR (400 MHz, $CDCl_3$) δ 7.97–7.93 (m, 2H), 6.95–6.91 (m, 2H), 3.86 (s, 3H), 2.95 (q, J = 7.3 Hz, 2H), 1.21 (t, J = 7.3 Hz, 3H) ppm.
^{13}C NMR (100 MHz, $CDCl_3$) δ 199.6, 163.4, 130.4, 130.2, 113.8, 65.6, 31.6, 8.6 ppm. ^{1}H and ^{13}C NMR spectras were in agreement with those reported in the literature.[1]

Deuterium-propiophenone (**2e**-d_1)

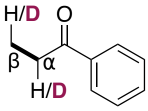

^{1}H NMR (400 MHz, $CDCl_3$) δ 7.99–7.96 (m, 2H), 7.58–7.53 (m, 1H), 7.48–7.44 (m, 2H), 3.04–2.95 (m, 1.45H), 1.24–1.19 (m, 2.64H) ppm.
^{13}C NMR (100 MHz, $CDCl_3$) δ 201.1, 137.1, 133.0, 128.7, 128.1, 31.9, 31.8 (t, $^{1}J_{C\text{-}D}$ = 19.2 Hz, product with deuterium in Cα), 8.3, 8.14 (t, $^{1}J_{C\text{-}D}$ = 19.9 Hz, product with deuterium in Cβ) ppm.

^{1}H and ^{13}C NMR spectra of allylic alcohol 1g-*d₁*

(*E*)-4-(4-Methoxyphenyl)but-3-en-2-*d*-2-ol (1g-*d*$_1$)

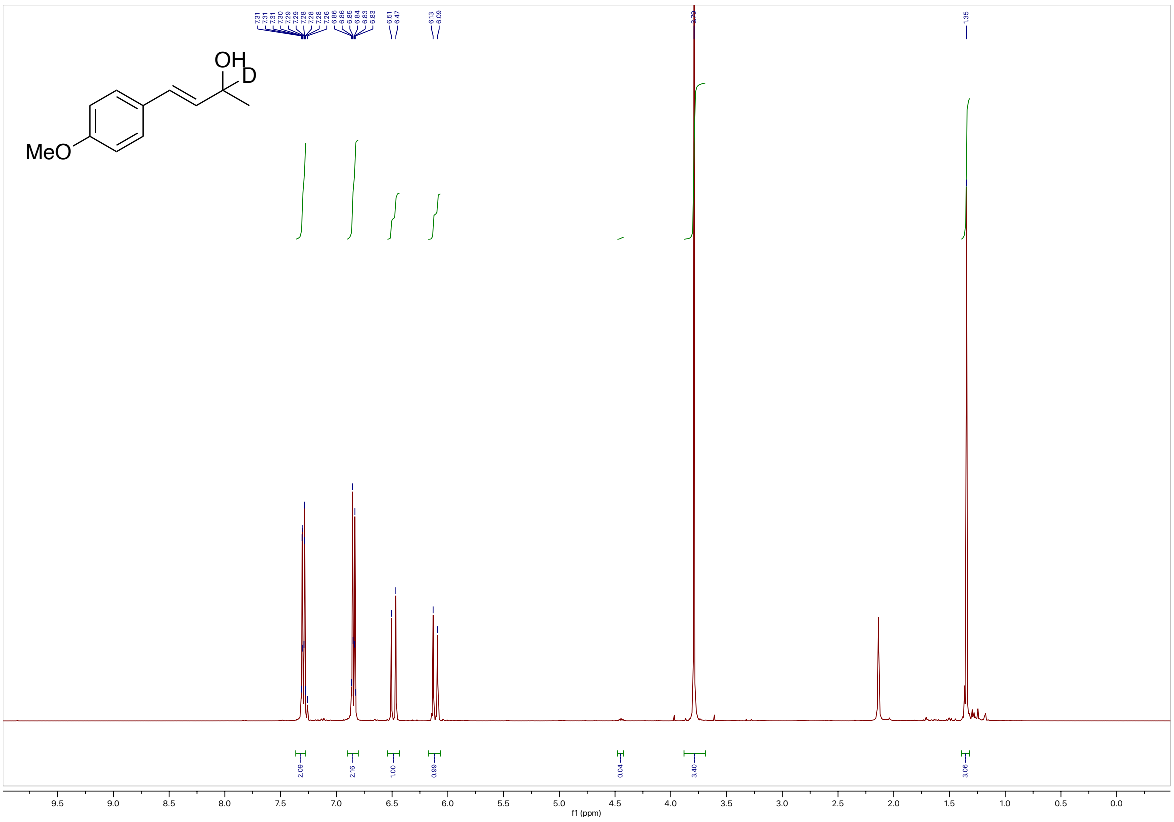

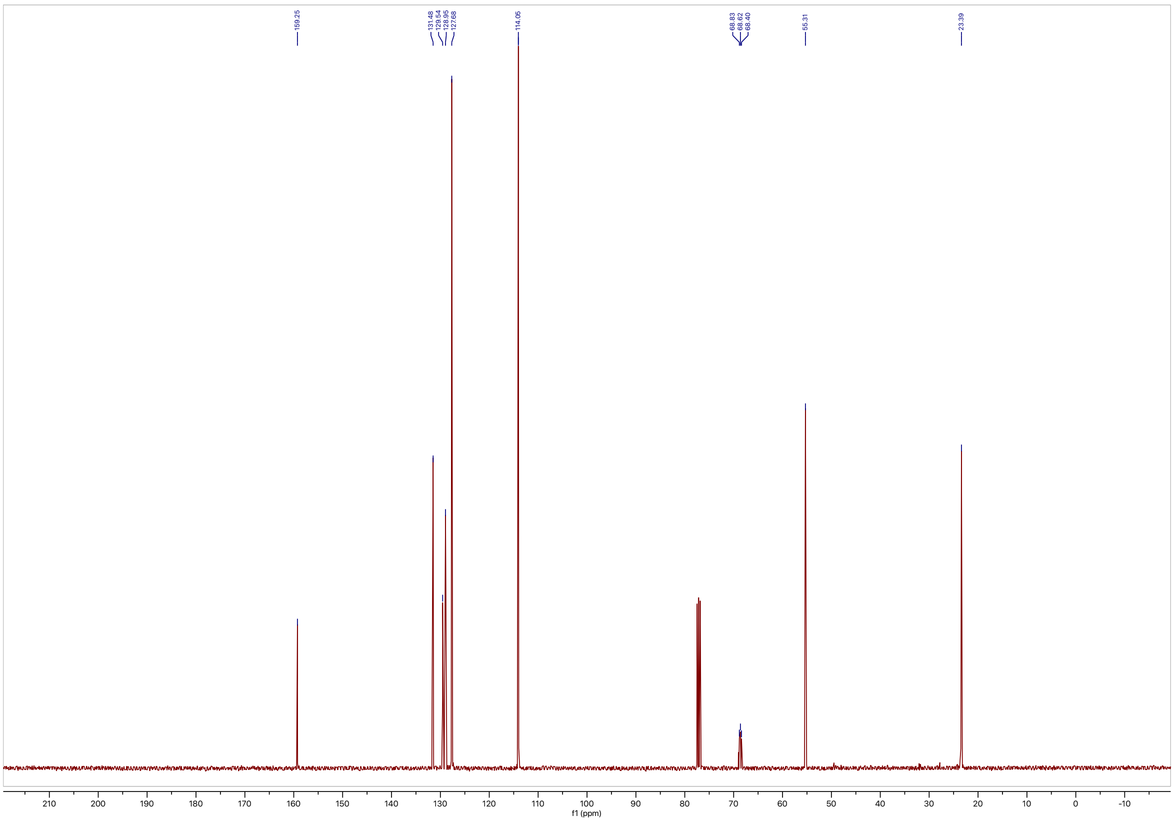

^{1}H and ^{13}C NMR spectra of ketones 2e-2f

Deuterium-propiophenone (2e-d_1)

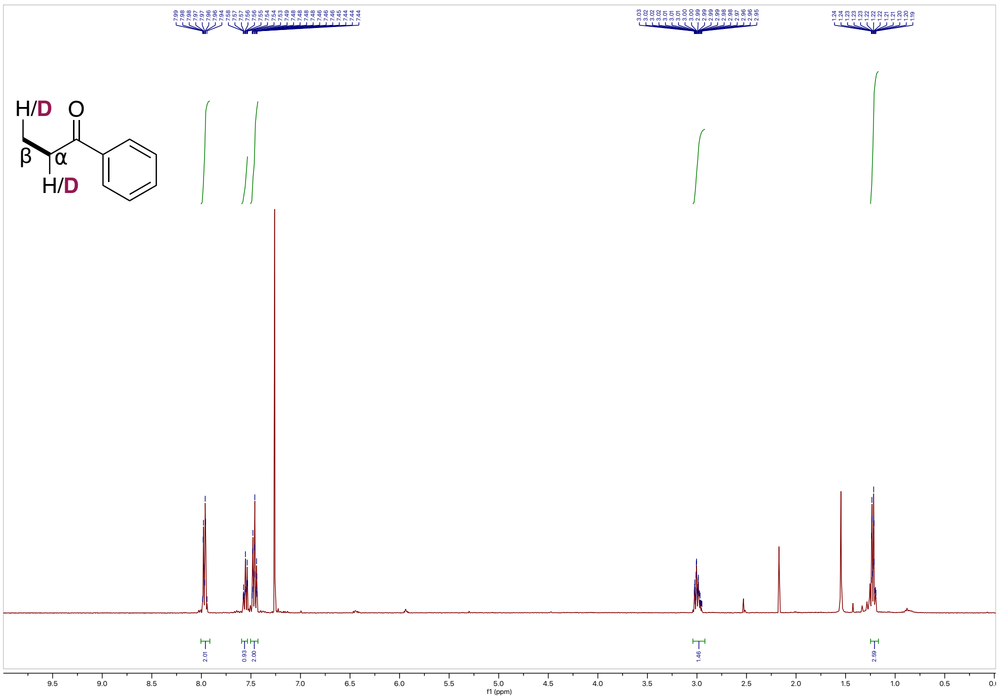

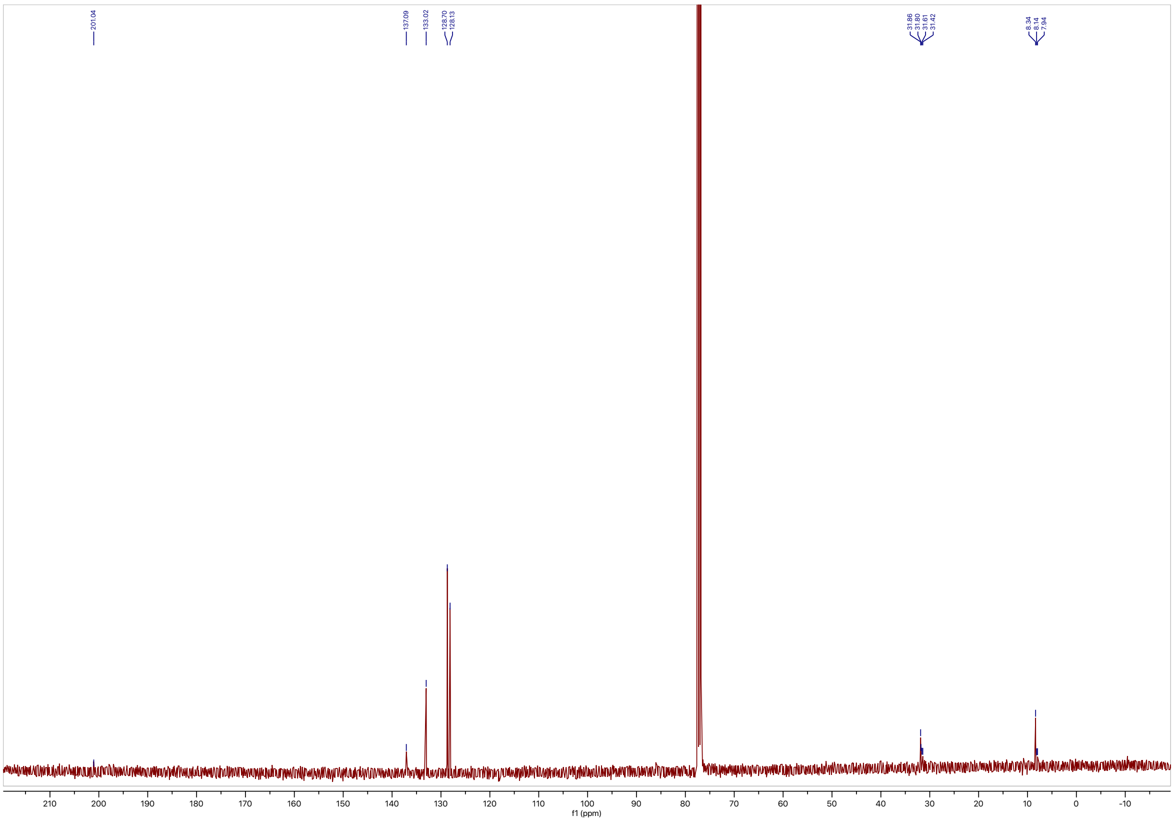

Deuterium-propiophenone (**2e-**d_1) when Acetone / D_2O (2:1) is used as solvent

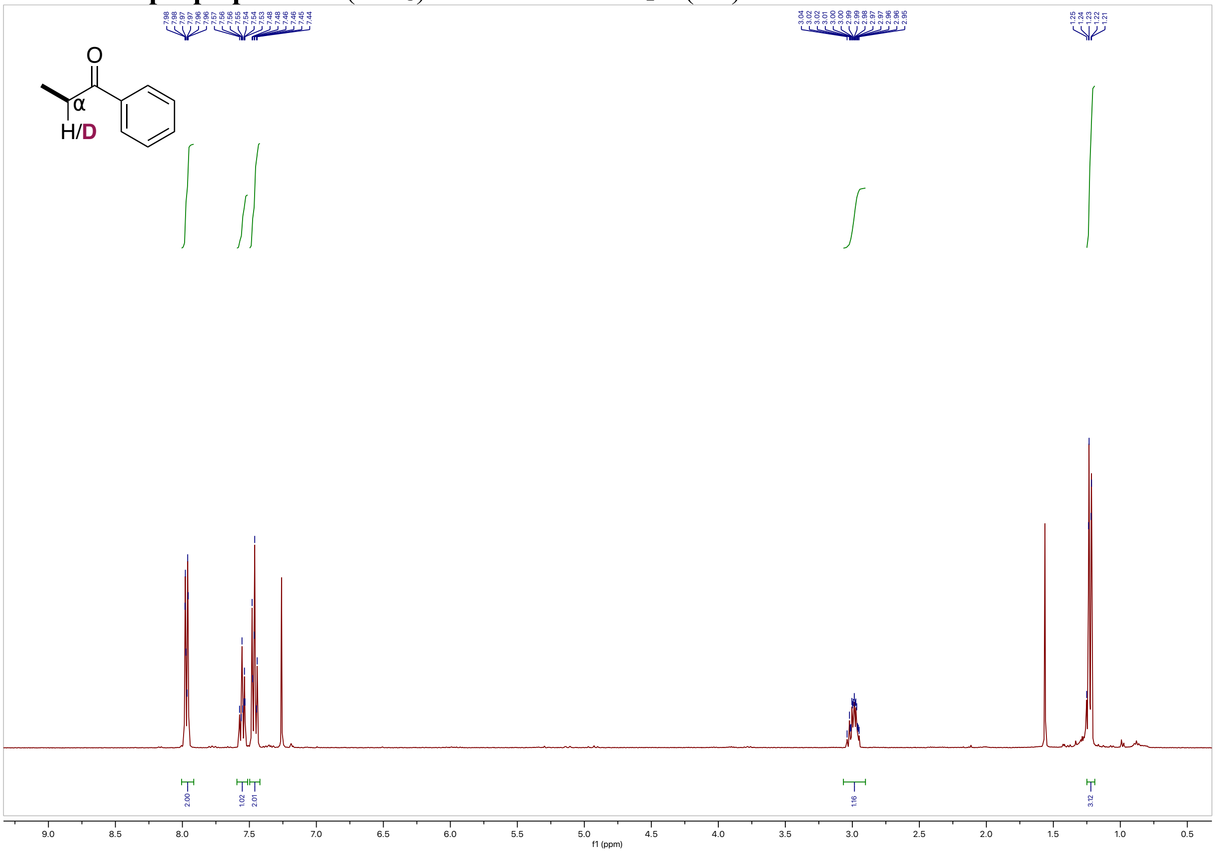

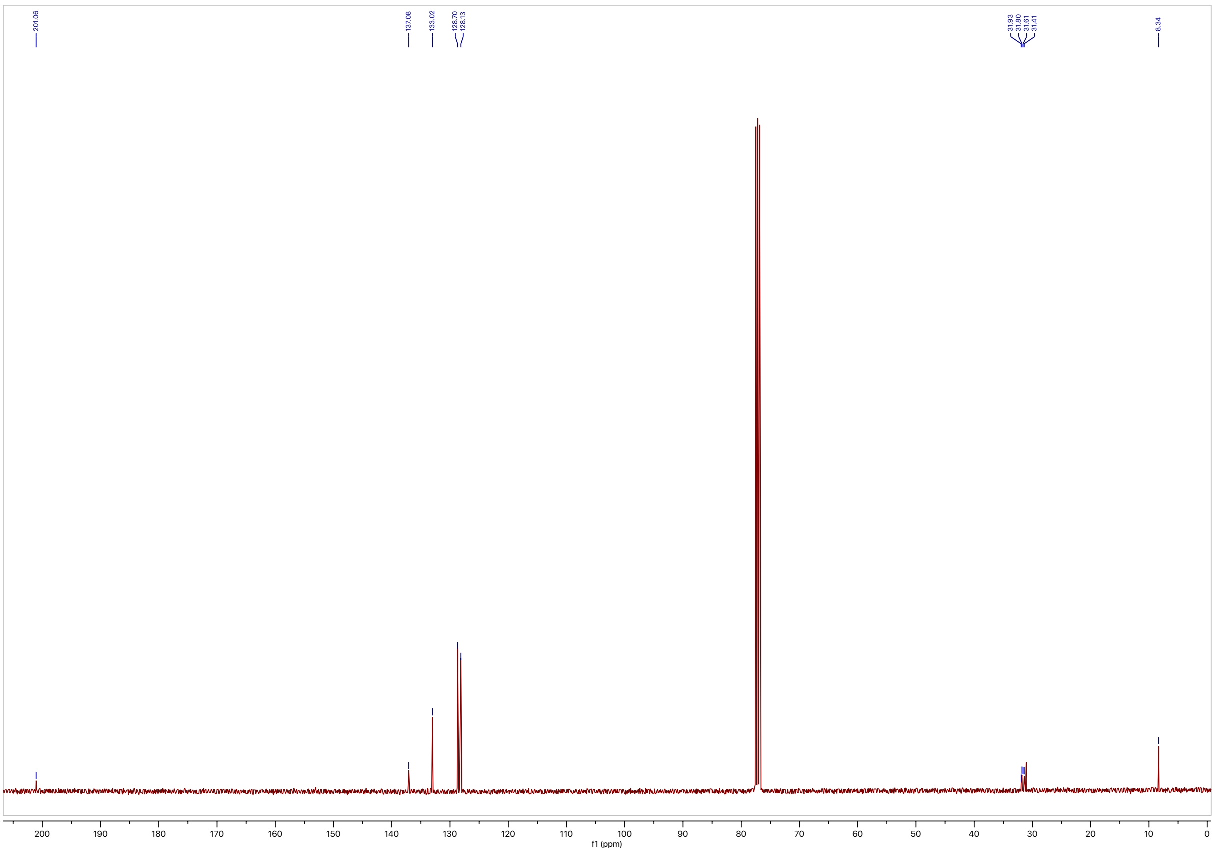
201.06
137.08
133.02
128.70
128.13
31.93
31.80
31.61
31.41
8.34
200
190
180
170
160
150
140
130
120
110
100
90
80
70
60
50
40
30
20
10
0
f1 (ppm)

Deuterium-1-phenylpentan-3-one (2f-*d*$_1$)

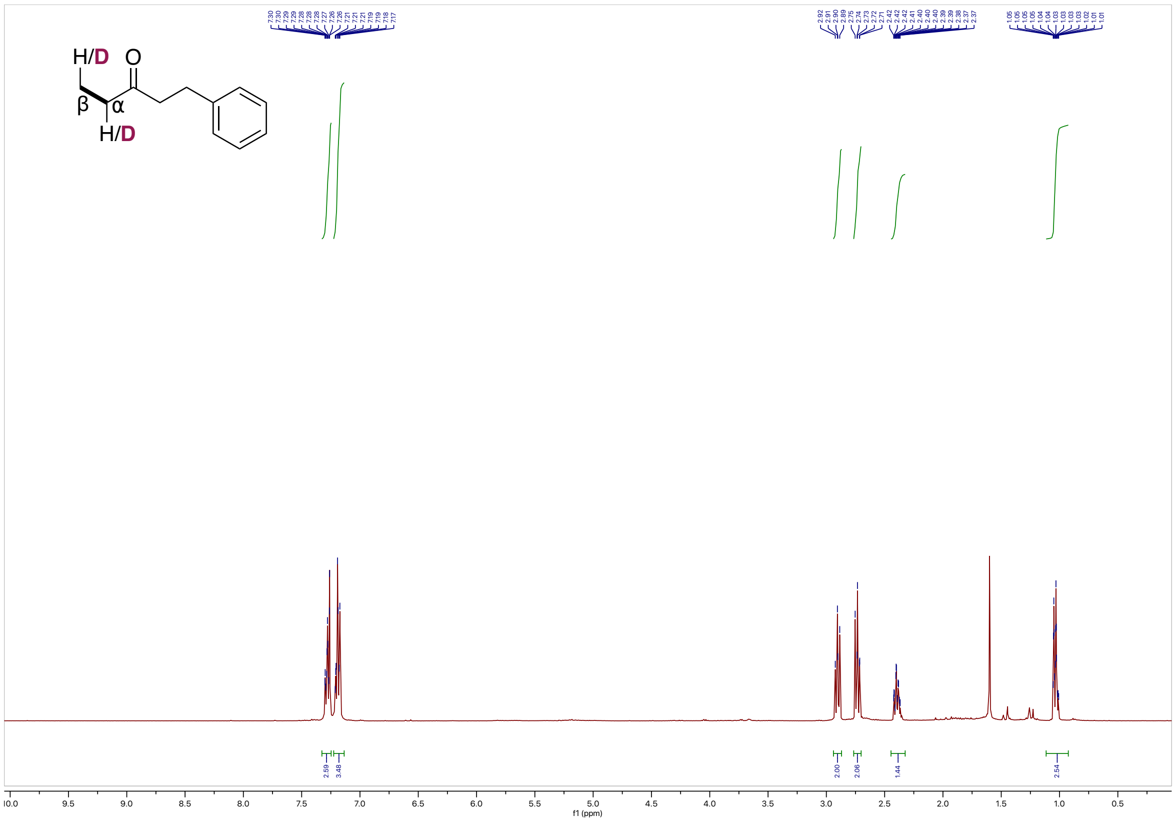

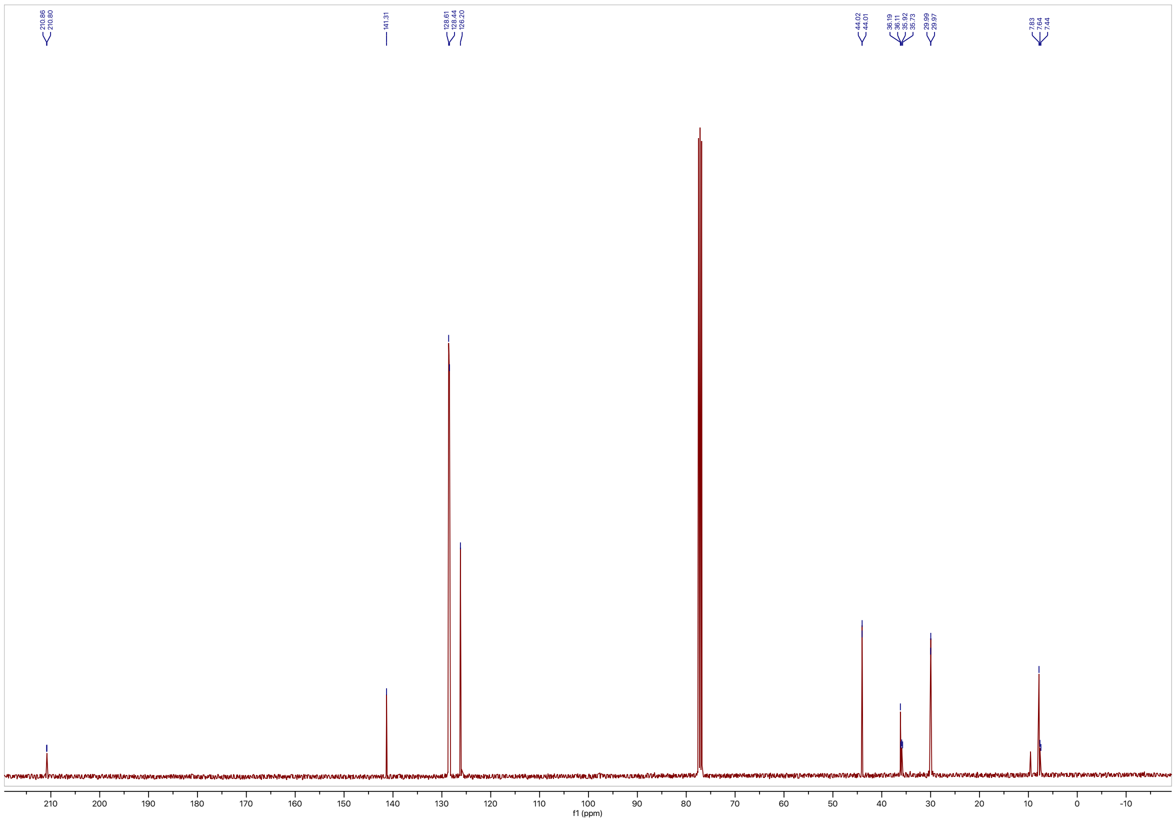

^{1}H and ^{13}C NMR spectra of α-chloroketones 3a-3e

4-Deuterium-3-chloro-4-phenylbutan-2-one (3a-d_1)

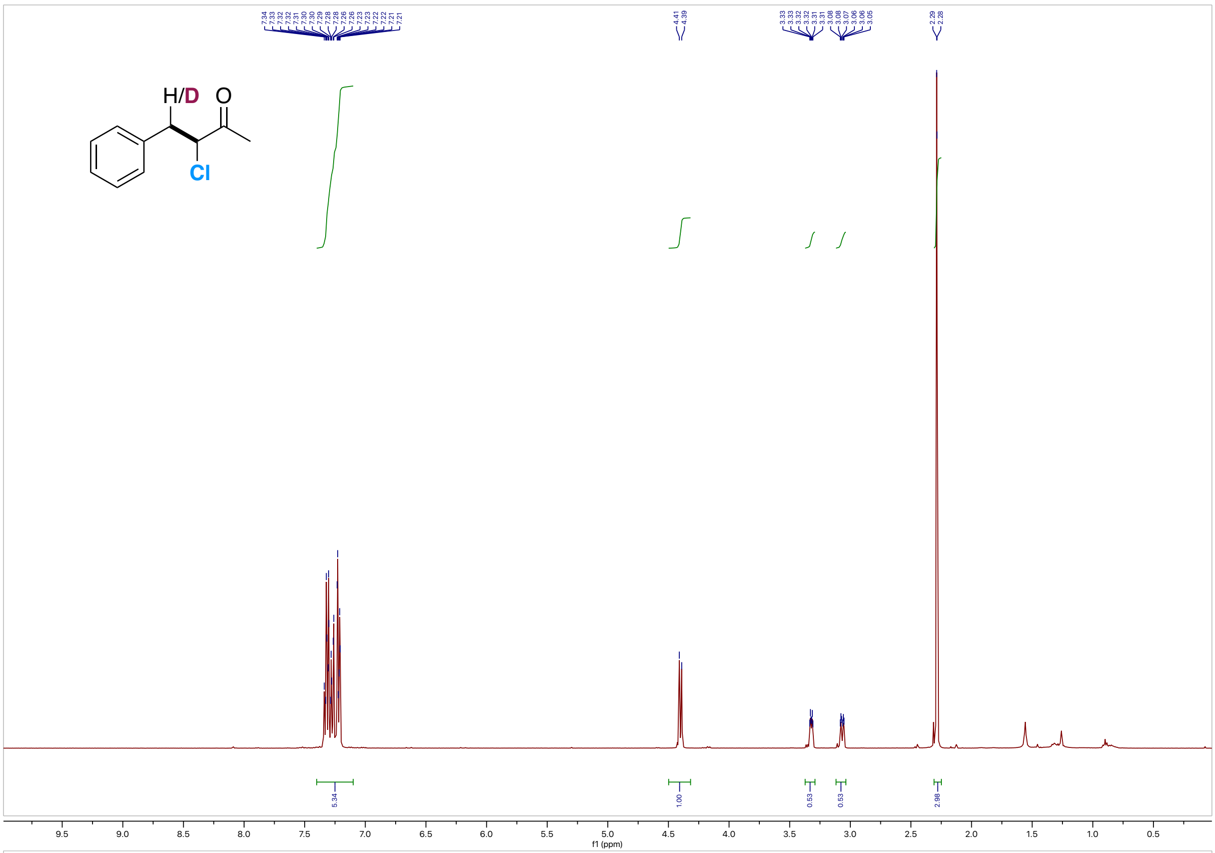

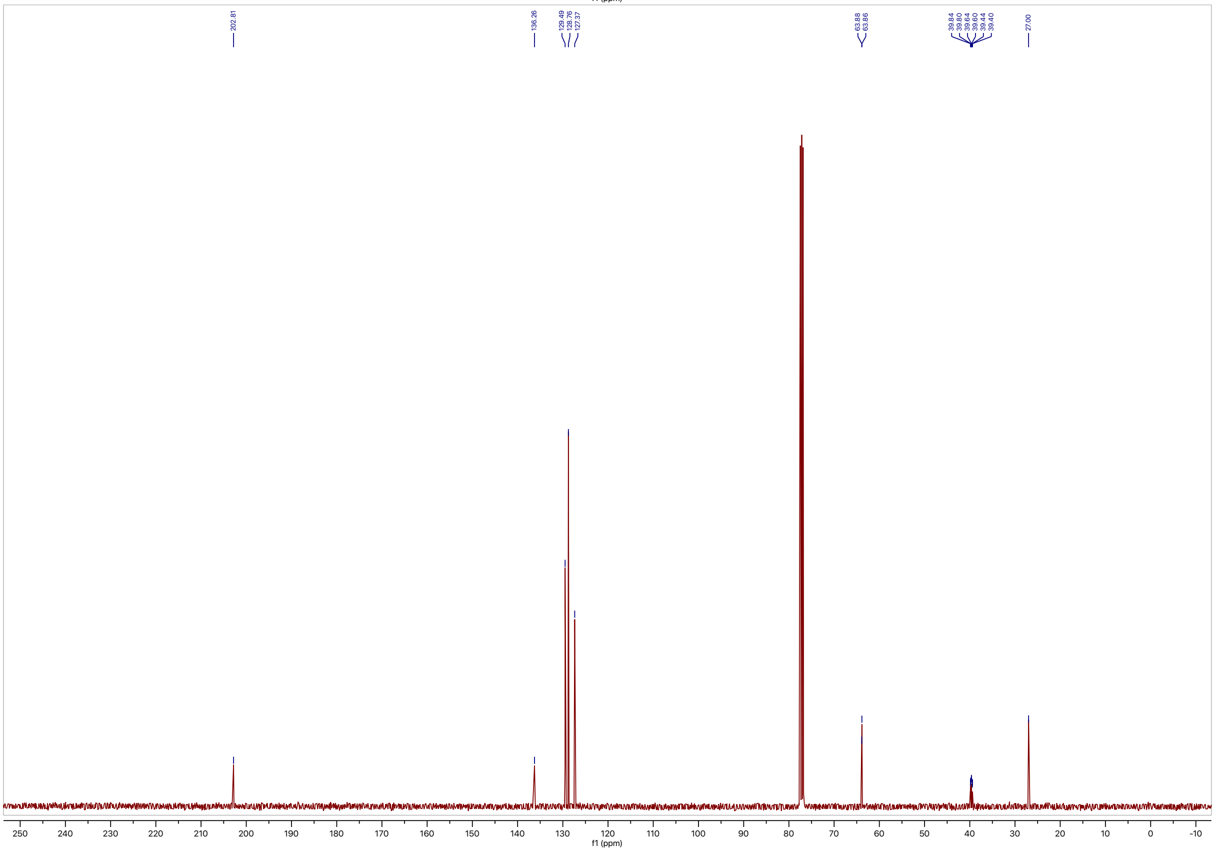

Deuterium-3-chlorooctan-2-one (**3b-*d*$_1$**)

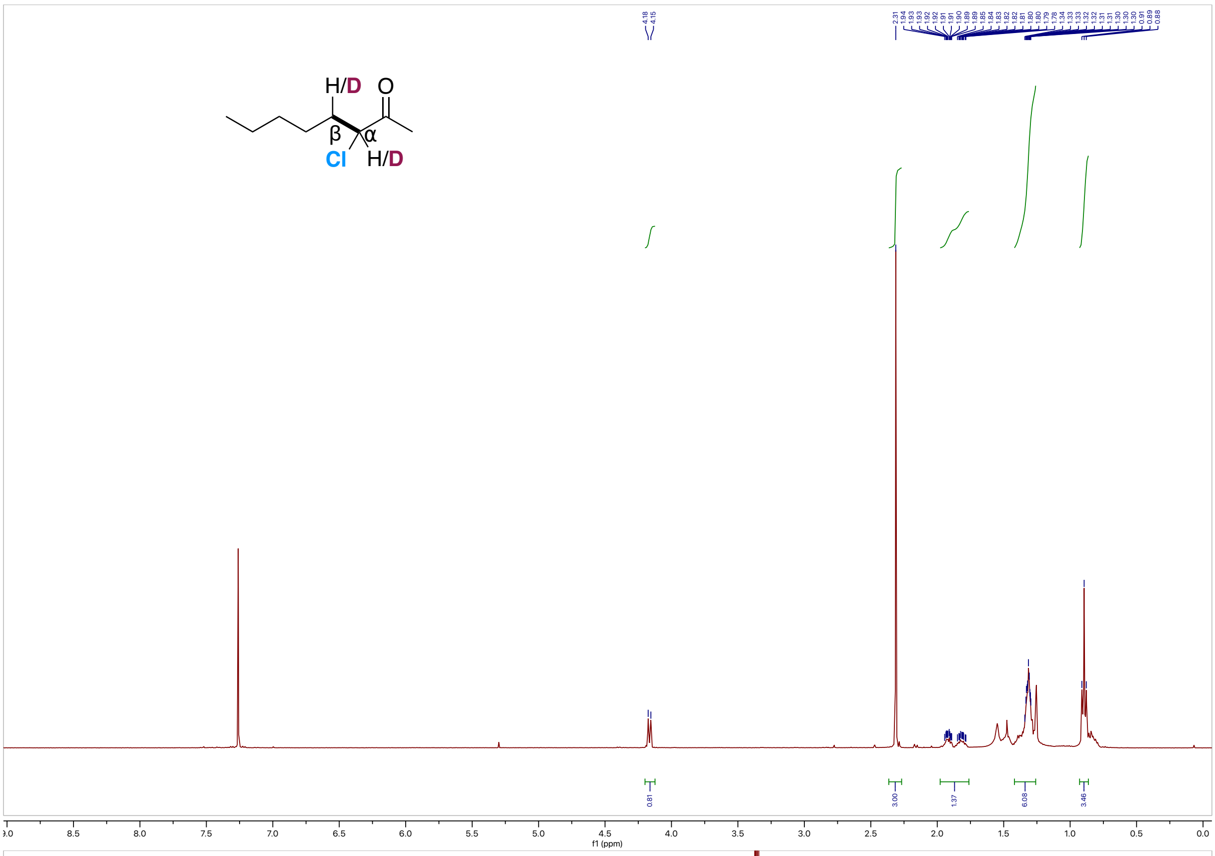

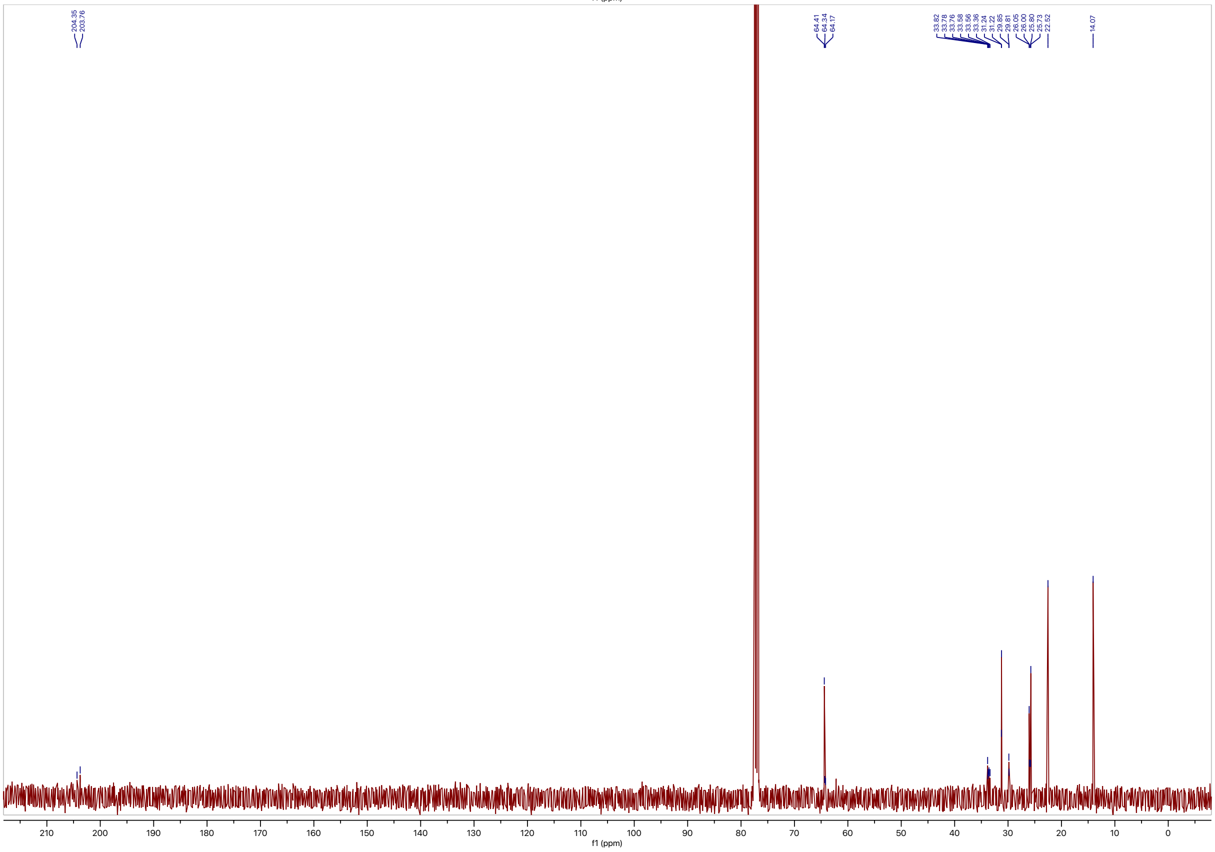

4-Deuterium-3-chloro-4-(4-methoxyphenyl)butan-2-one (3g-d_1)

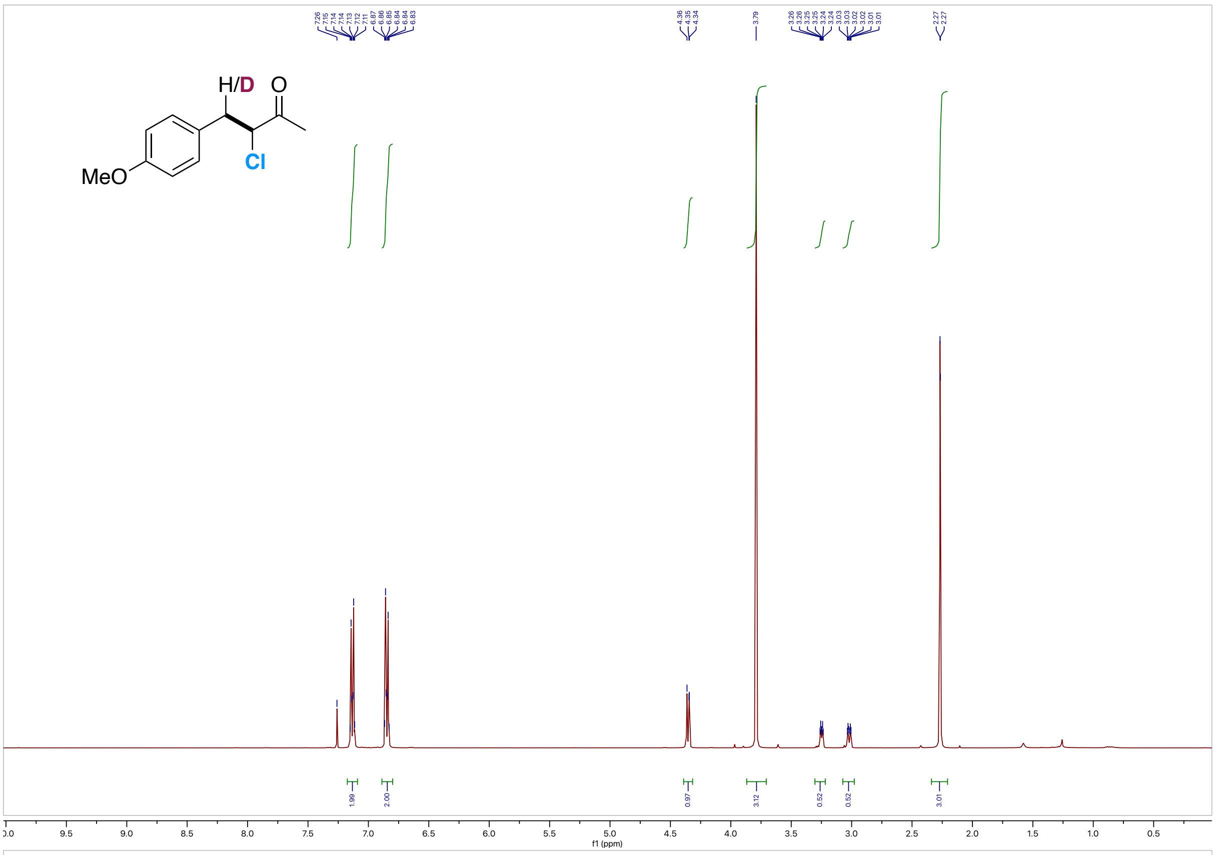

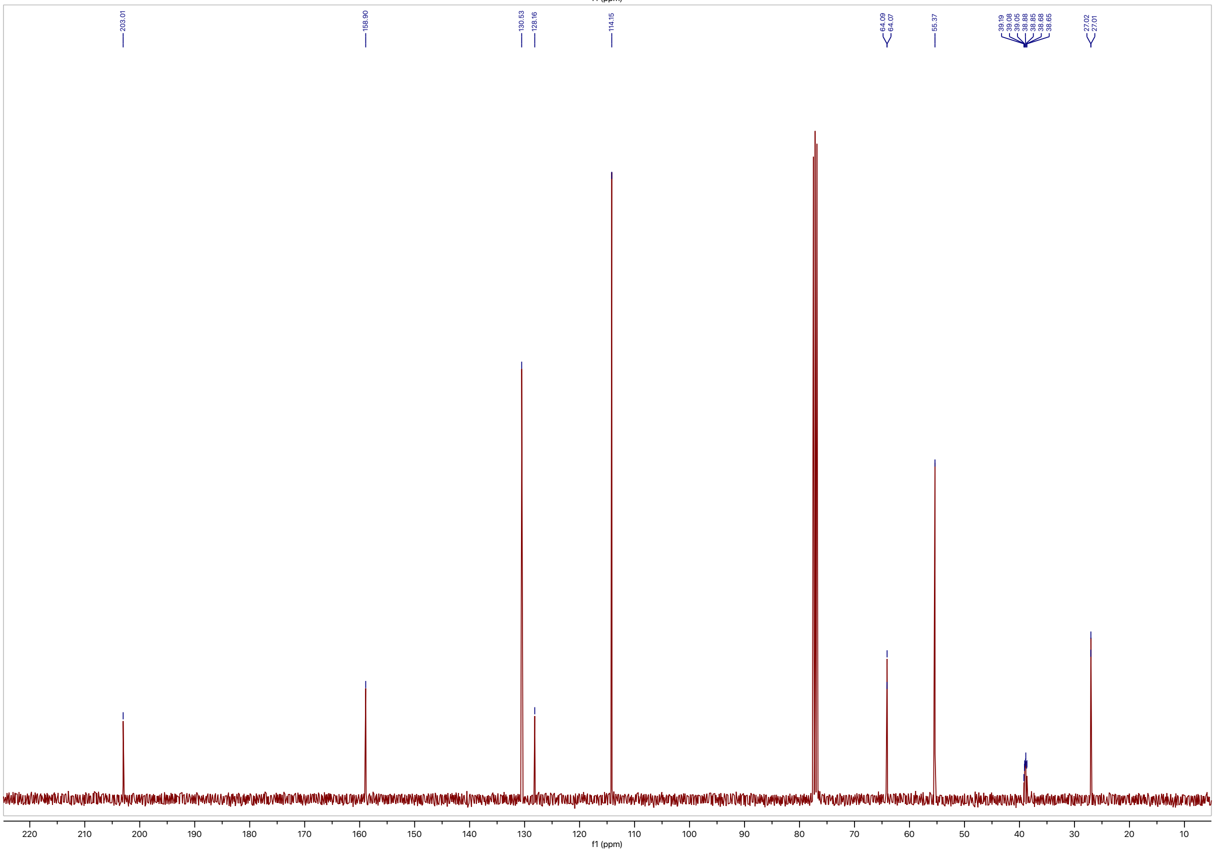

Deuterium-2-chloro-1-phenylpropan-1-one (**3e**-d_1)

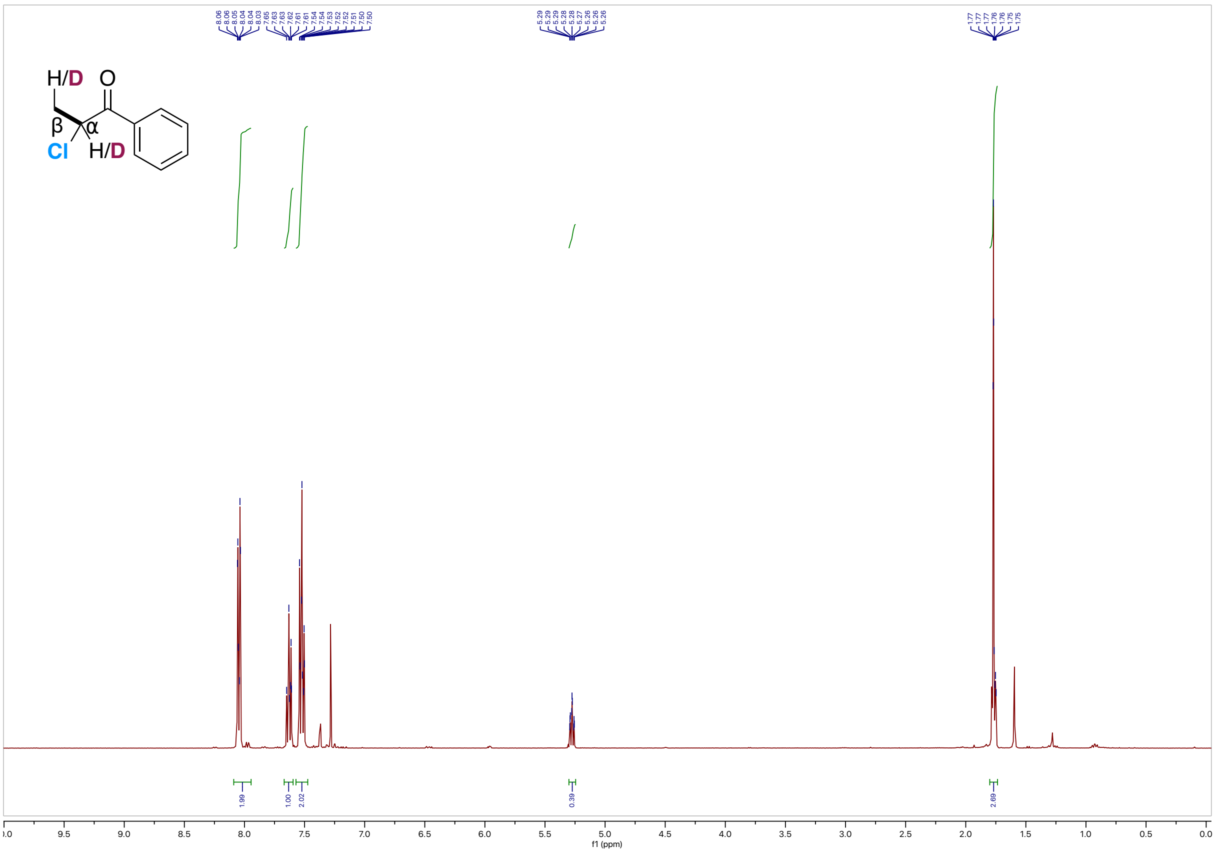

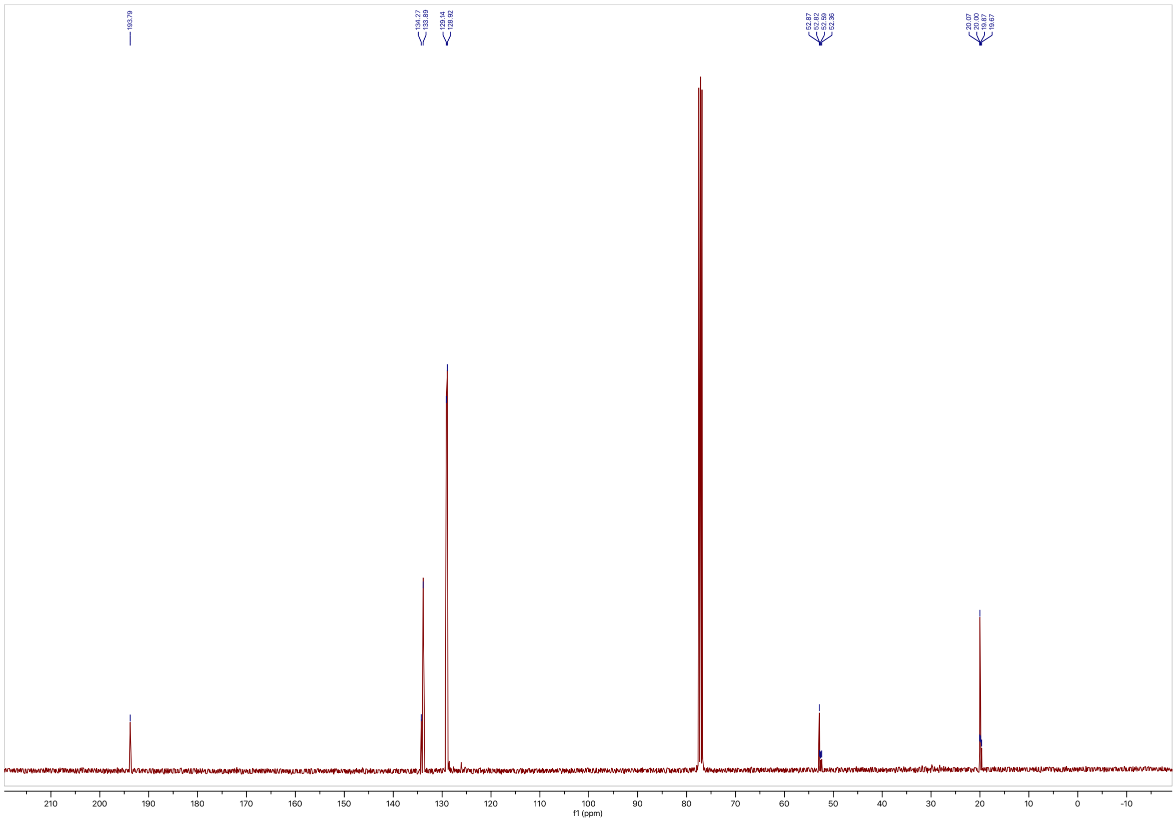

^{1}H and ^{13}C NMR spectra of deuterium labeling cross-over studies

4-Deuterium-4-phenylbutan-2-one (2a-d_1)

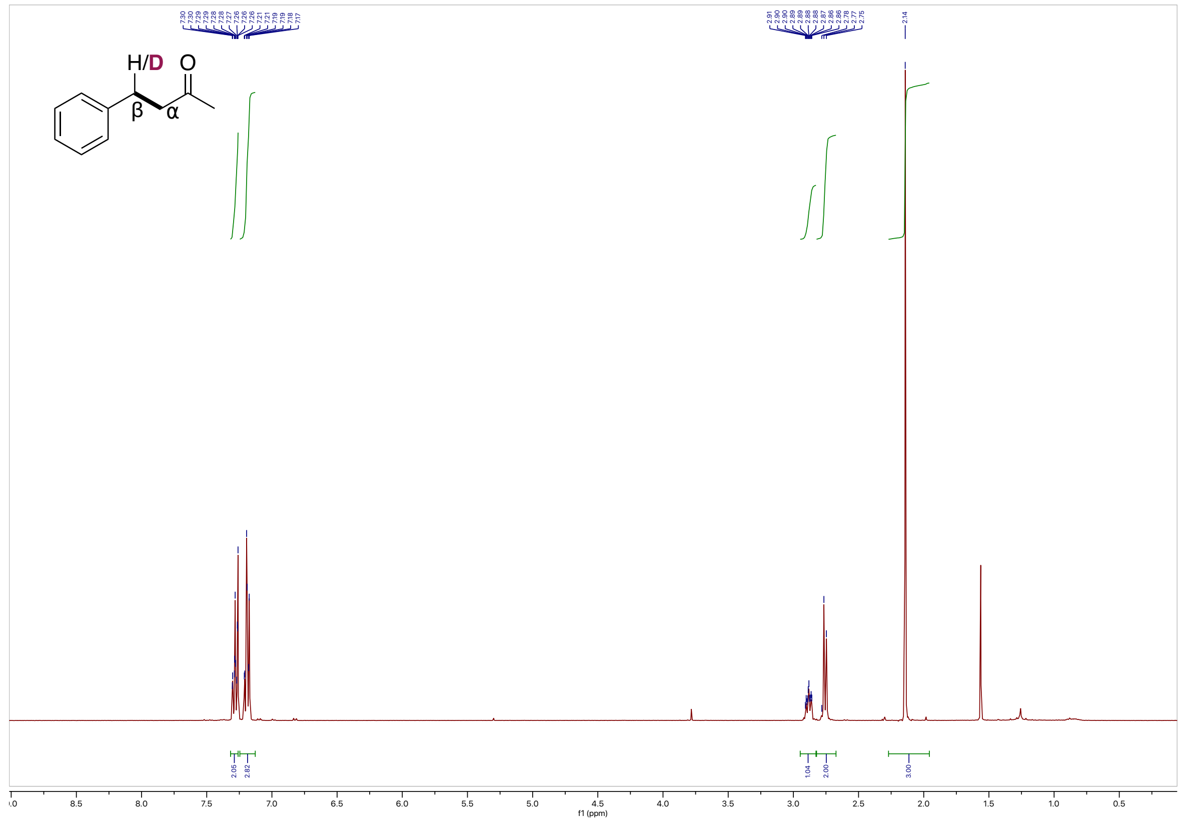

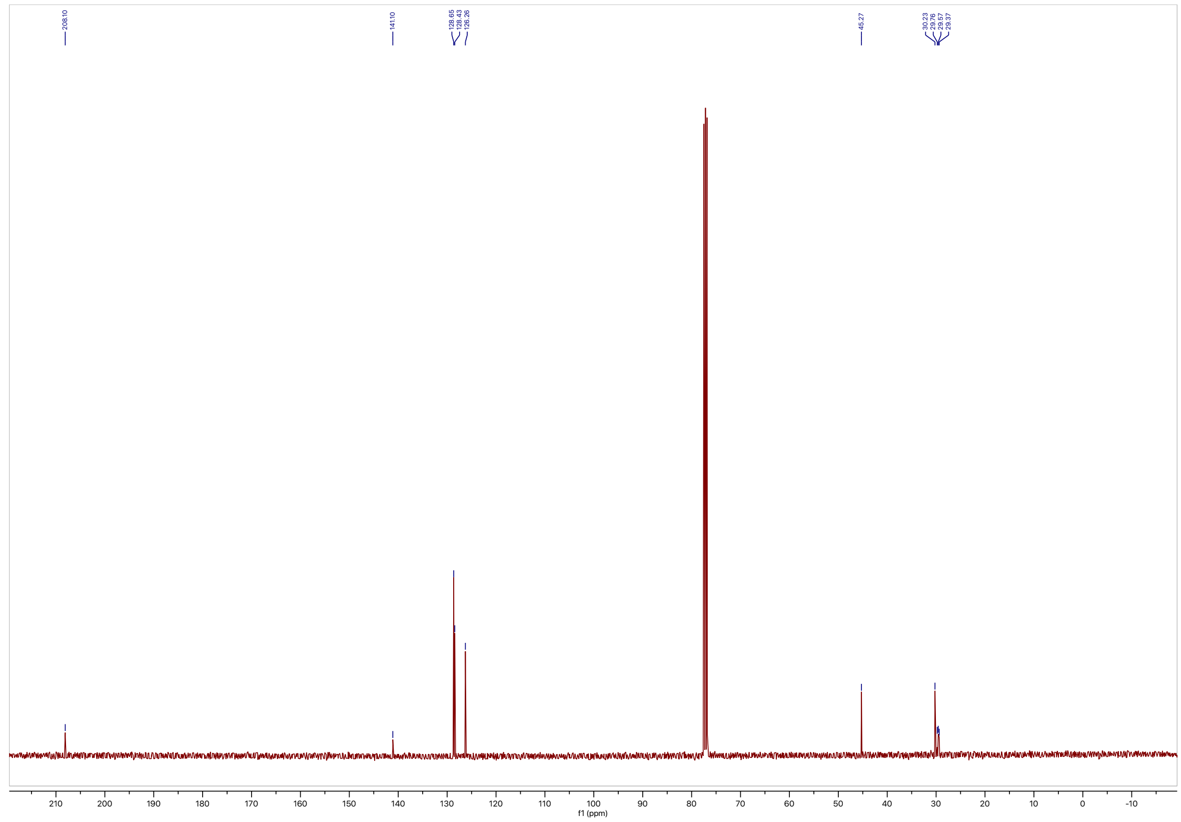

4-(4-Methoxyphenyl)butan-2-one (2g)

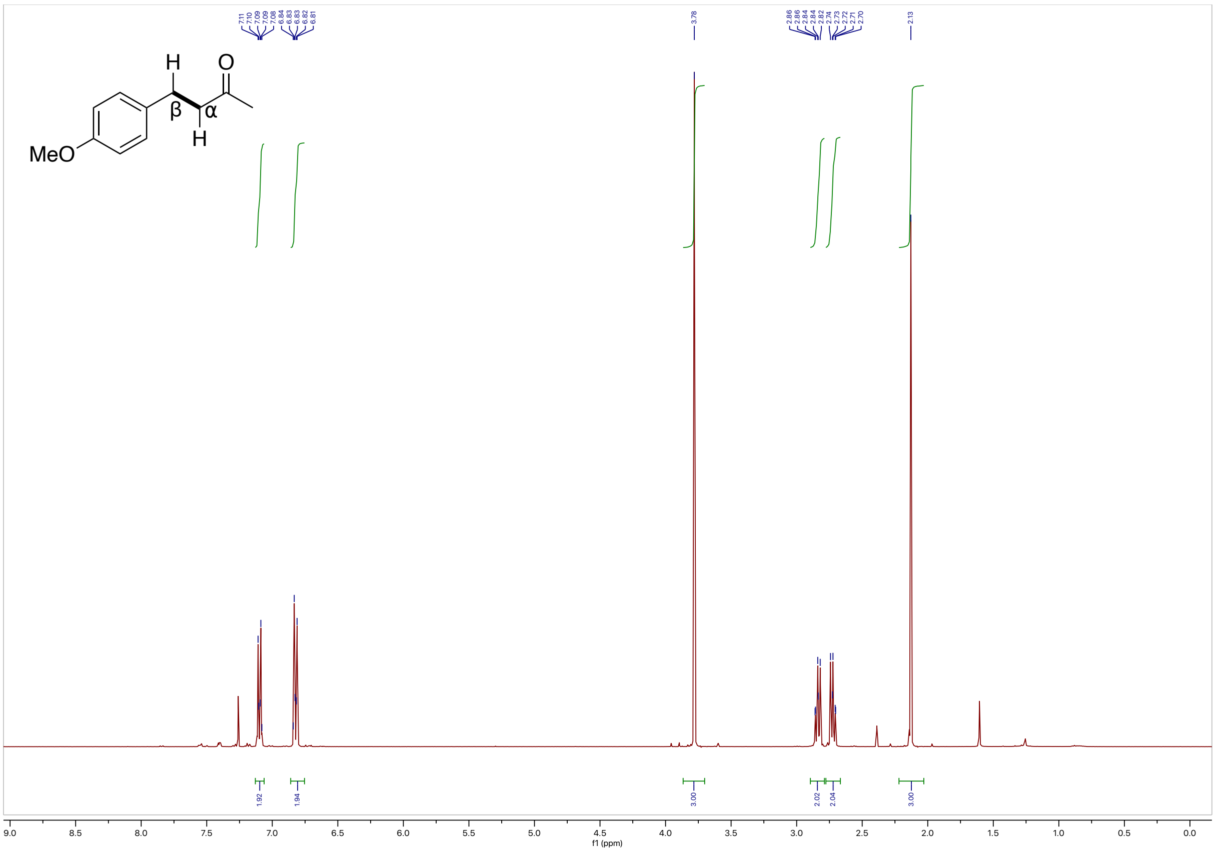

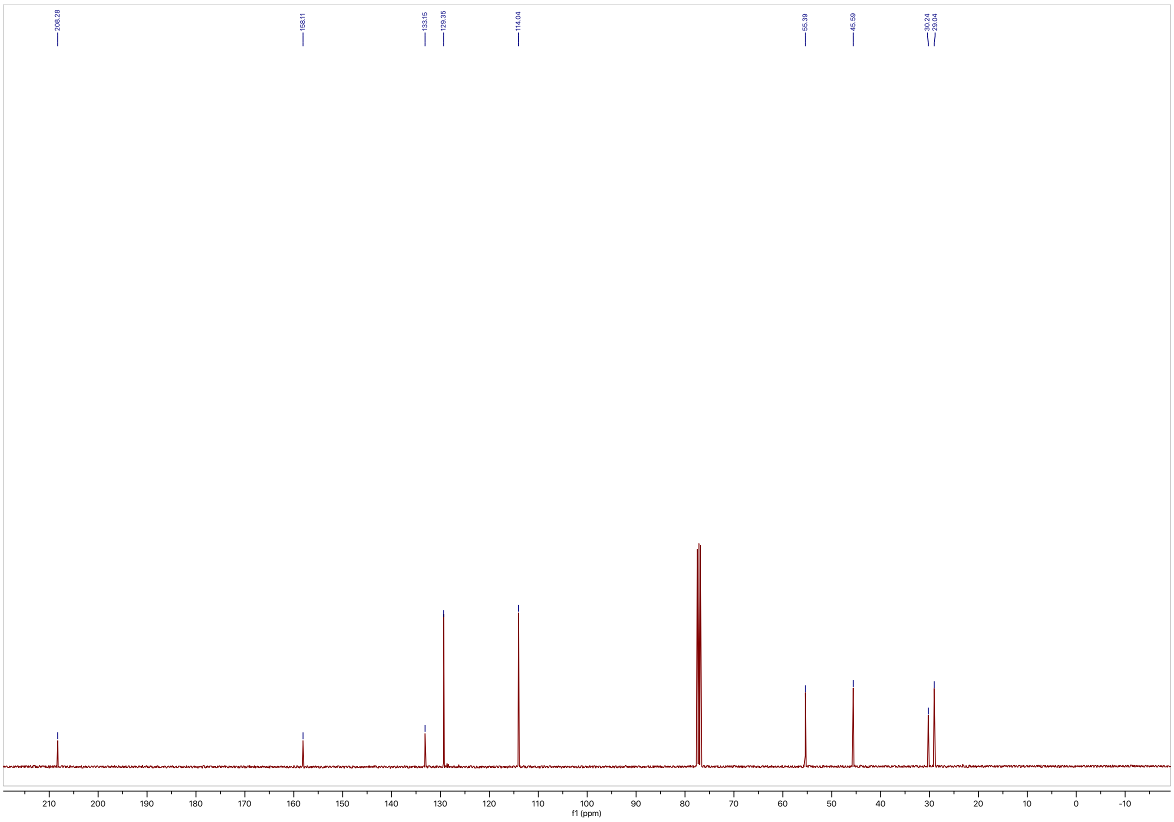

1-(4-Methoxyphenyl)propan-1-one (2h)

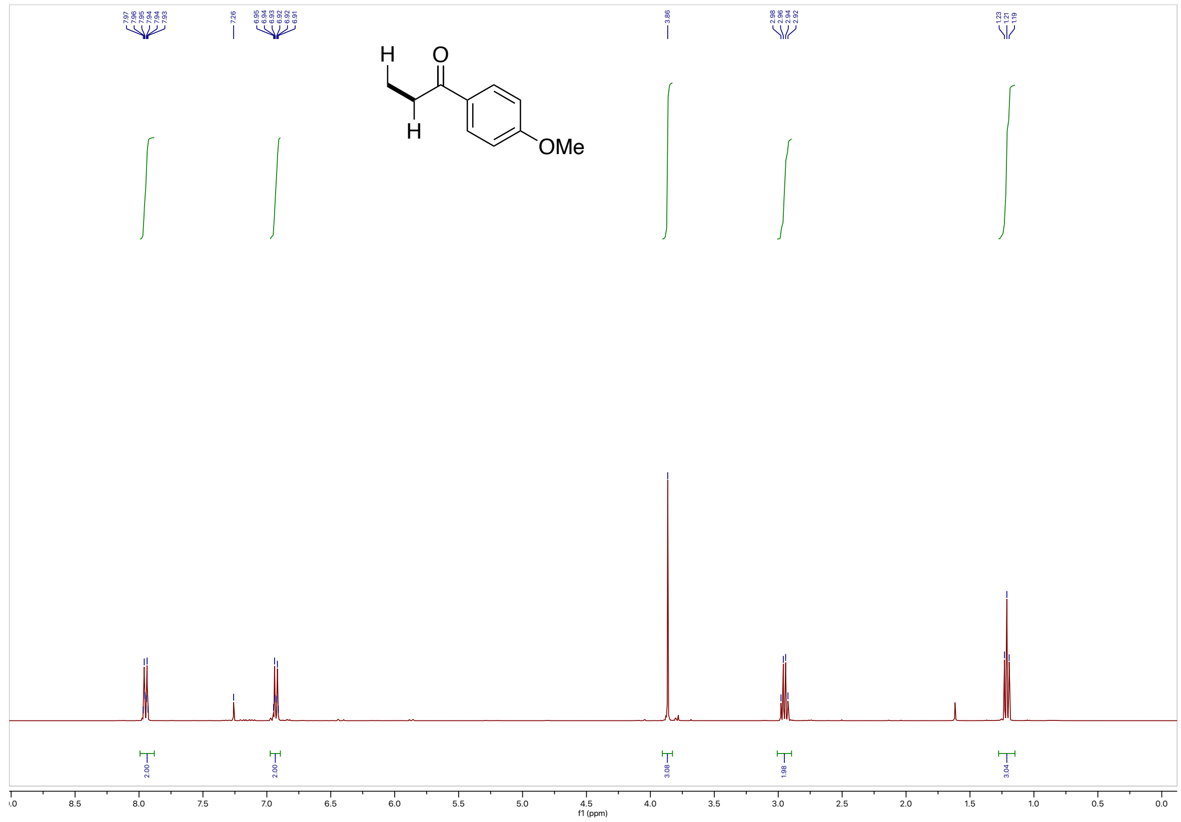

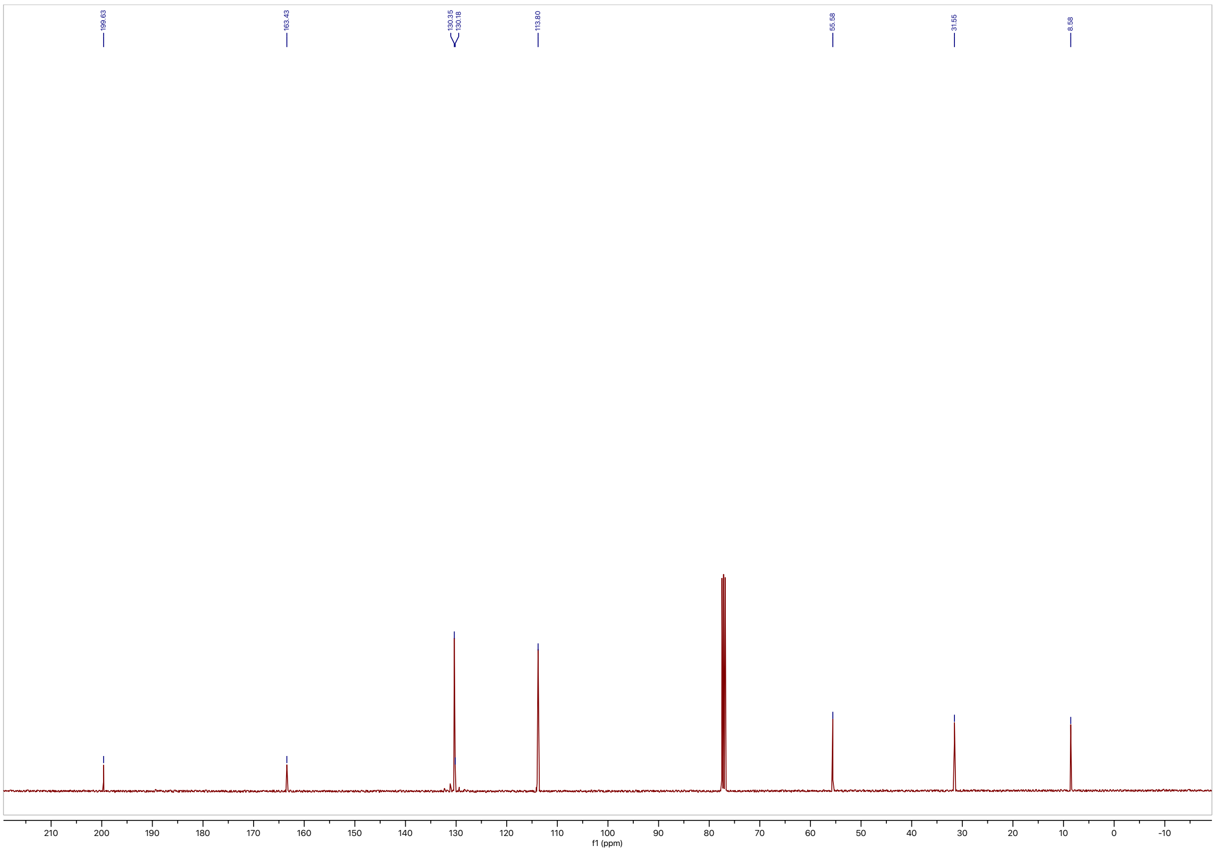

Deuterium-propiophenone (2e-*d₁*)

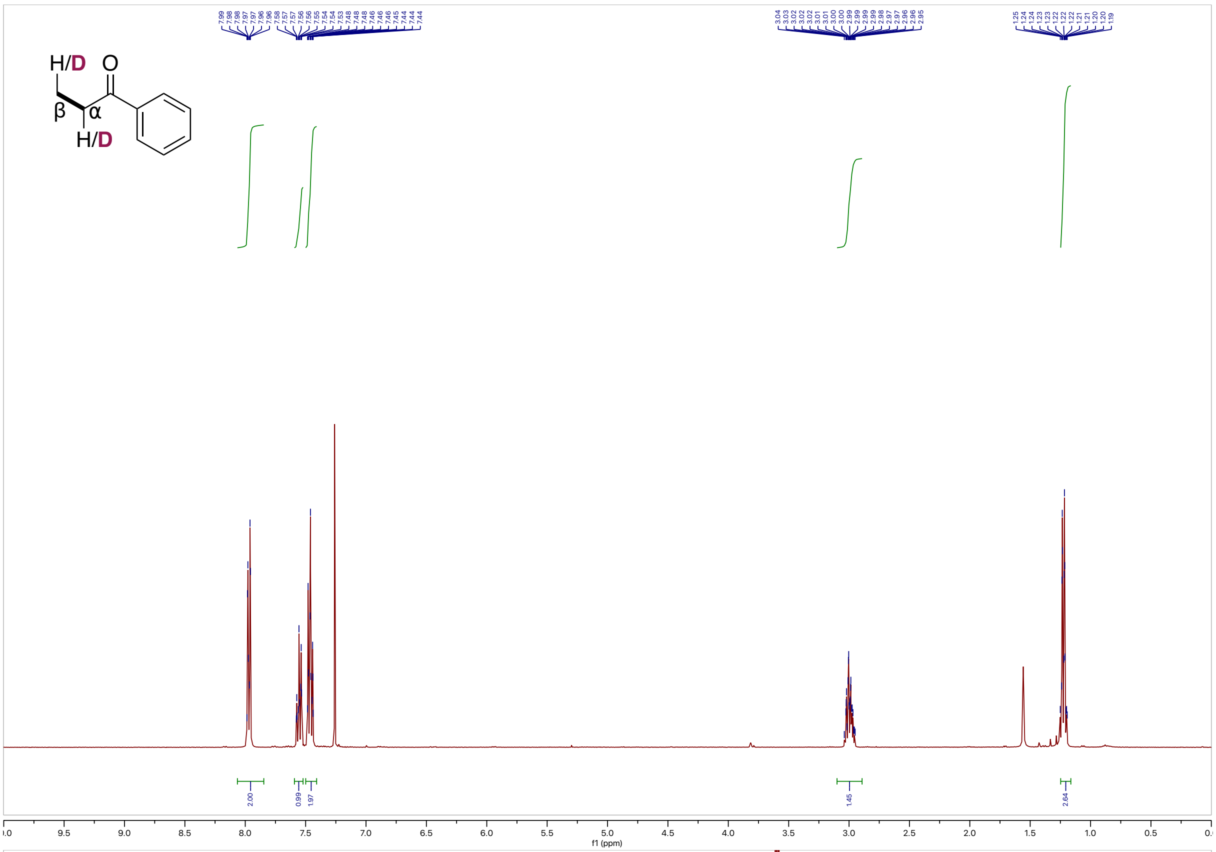

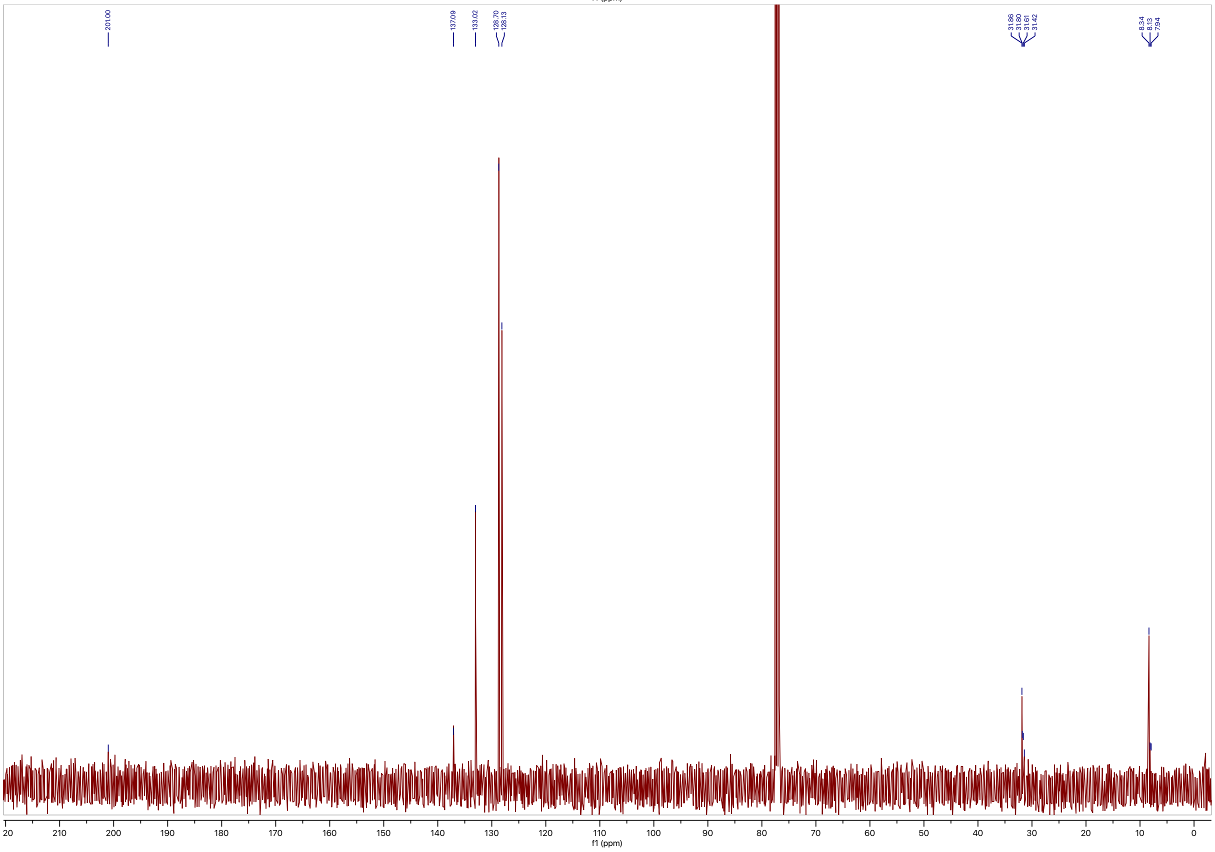

DFT studies

Reaction profile of the Ir(III) catalyzed isomerization and isomerization-chlorination of allylic alcohol 1b

The mechanism of the Ir-catalyzed isomerization and isomerization-chlorination of 1,2-disubstituted allylic alcohol **1b** was also investigated by DFT calculations. The calculated Gibbs energy profile is depicted in Figure S5 and the corresponding optimized structures are given in Figure S6.

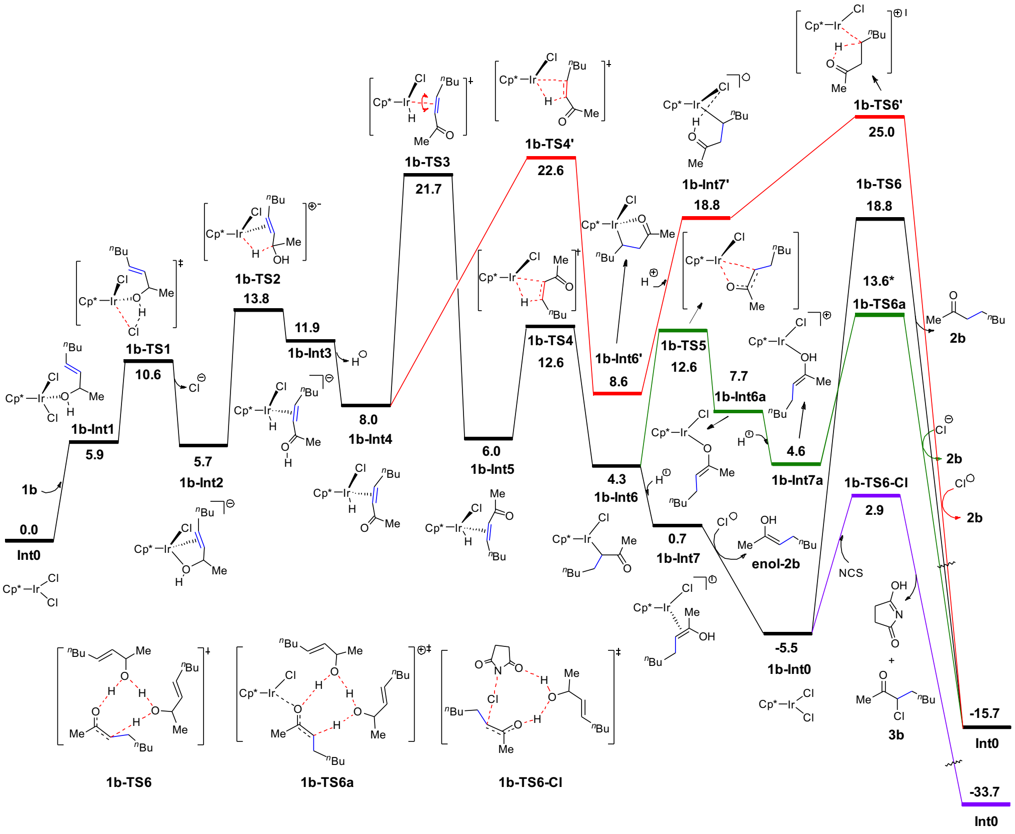

Figure S5. Calculated Gibbs energy profile (kcal/mol) for the Ir-catalyzed isomerization and isomerization-chlorination of the 1,2-disubstituted allylic alcohol **1b**.

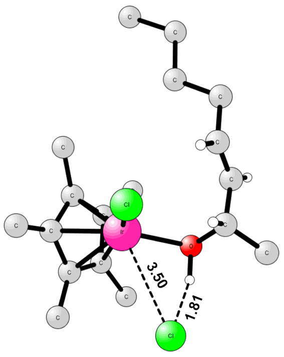

1b-TS1

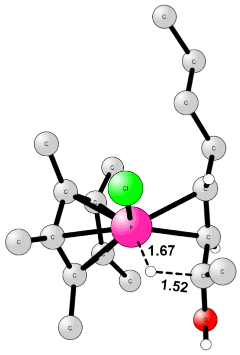

1b-TS2

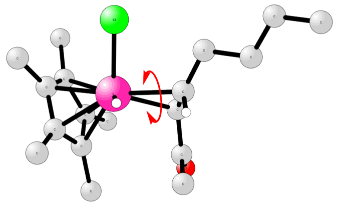

1b-TS3

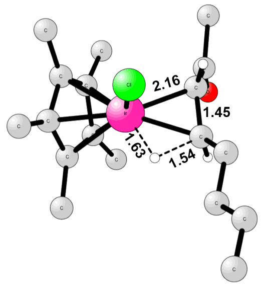

1b-TS4

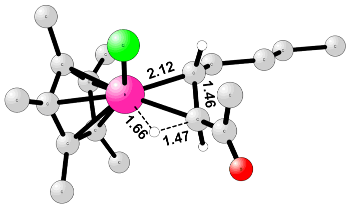

1b-TS4'

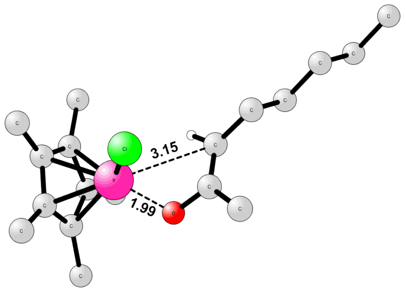

1b-TS5

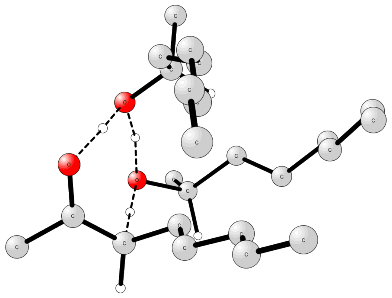

1b-TS6

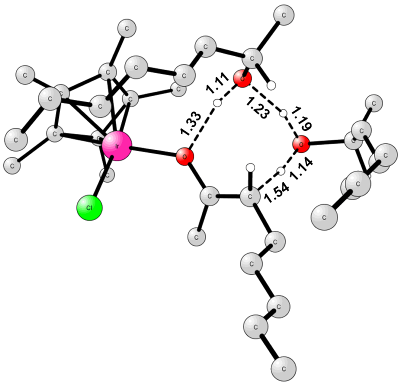

1b-TS6a

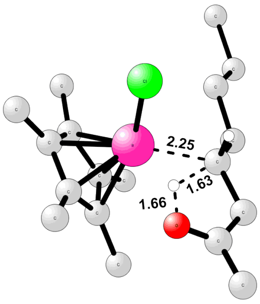

1b-TS6'

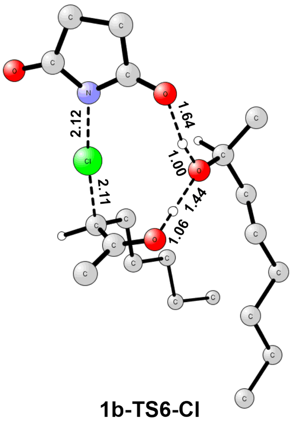

1b-TS6-Cl

Figure S6. Optimized transition state structures for the Ir-catalyzed isomerization and isomerization-chlorination of the 1,2-disubstituted allylic alcohol **1b**. Most hydrogen atoms are omitted for clarity.

Reaction profile of the Ir(III) catalyzed isomerization and isomerization-chlorination of allylic alcohol 1e in acetone

The iridium-catalyzed isomerization and isomerization-chlorination of **1e** were also investigated in pure acetone solvent. The obtained mechanism is summarized in Scheme S4 and the calculated Gibbs energy profile is given in Figure S7. Additional results are shown in Figures S8.

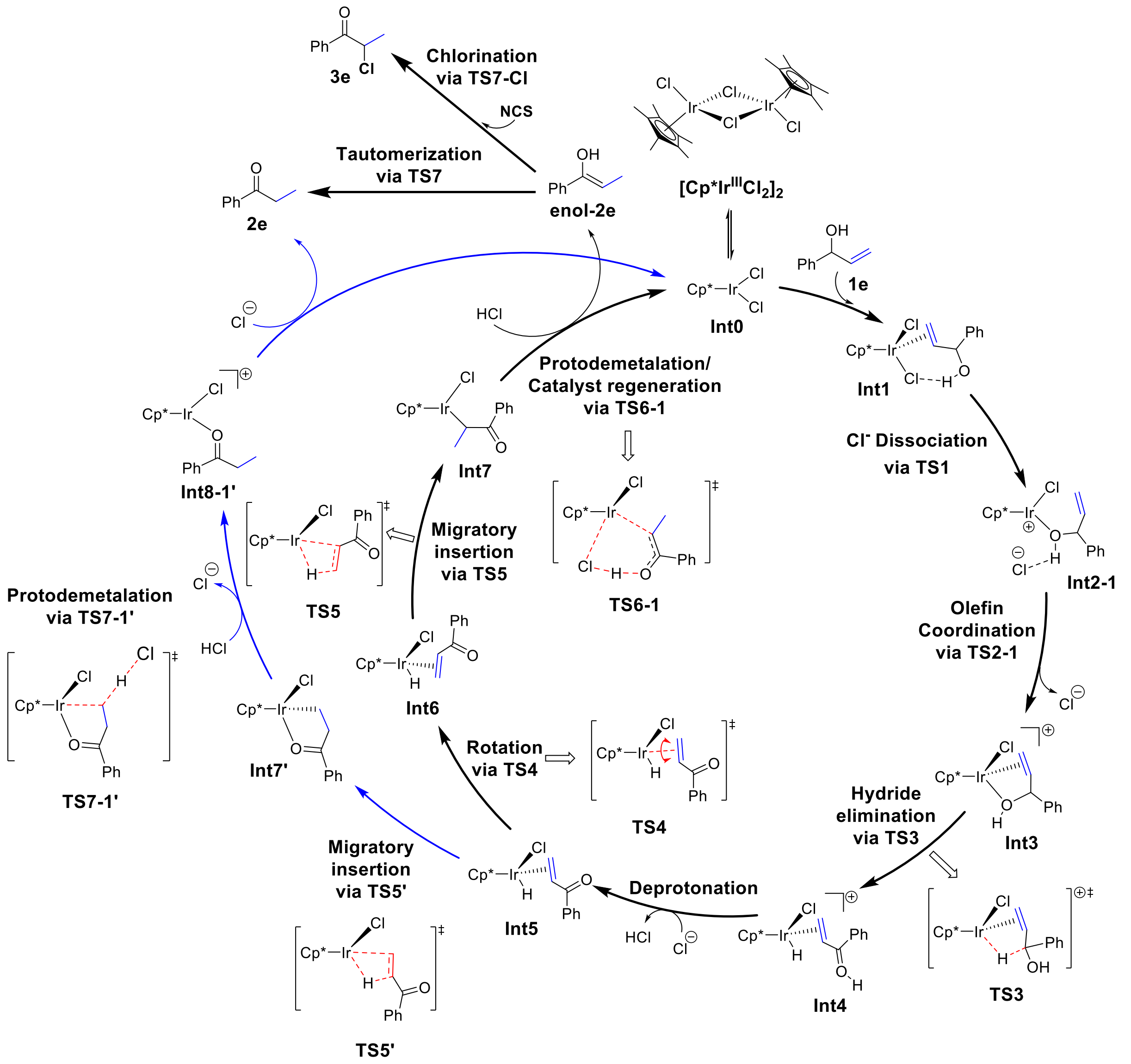

Scheme S4. Catalytic cycle for iridium-catalyzed isomerization and isomerization-chlorination of **1e** in acetone.

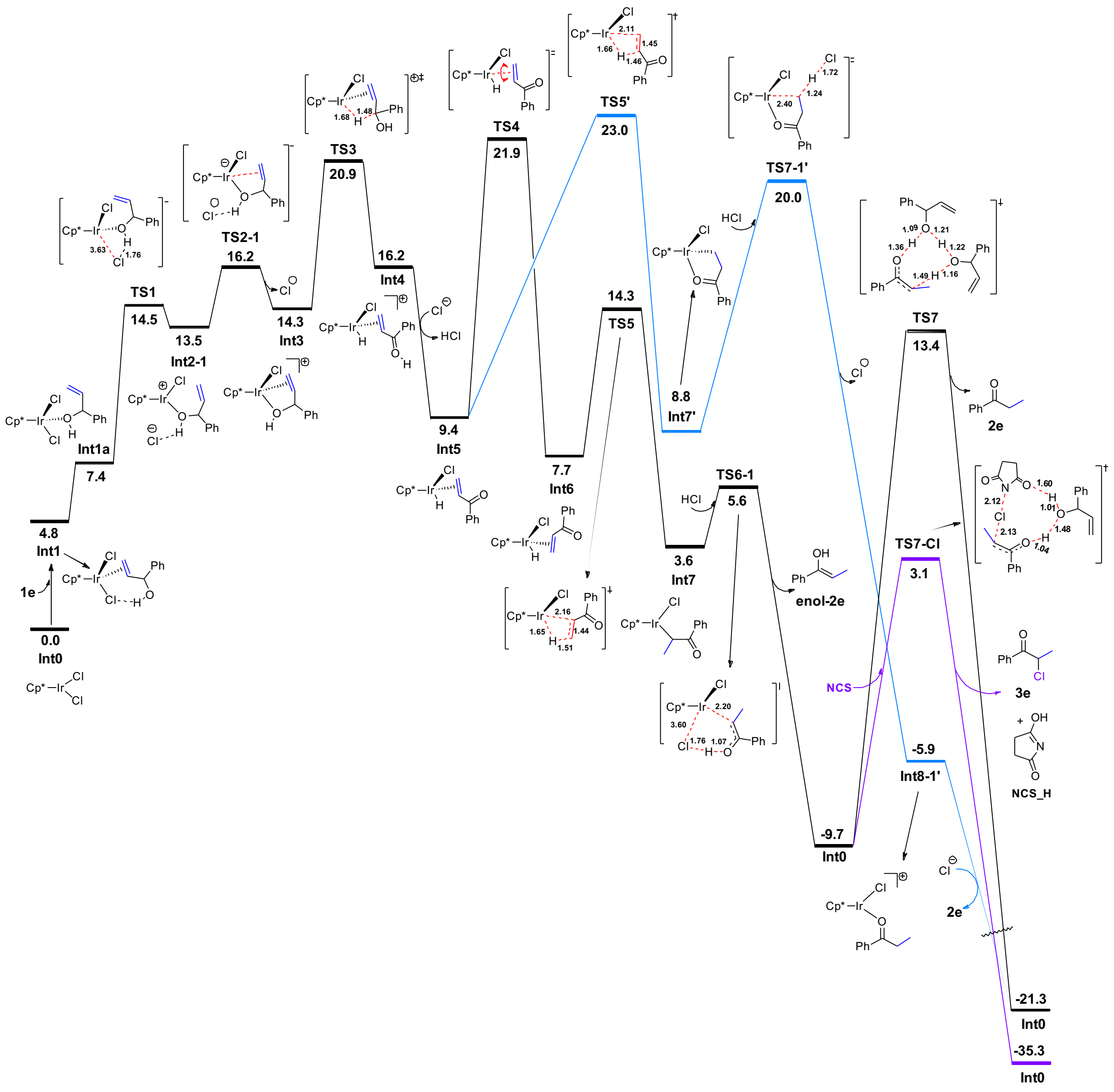

Figure S7. Calculated Gibbs energy profile (kcal/mol) for the iridium-catalyzed isomerization and isomerization-chlorination of **1e** in acetone.

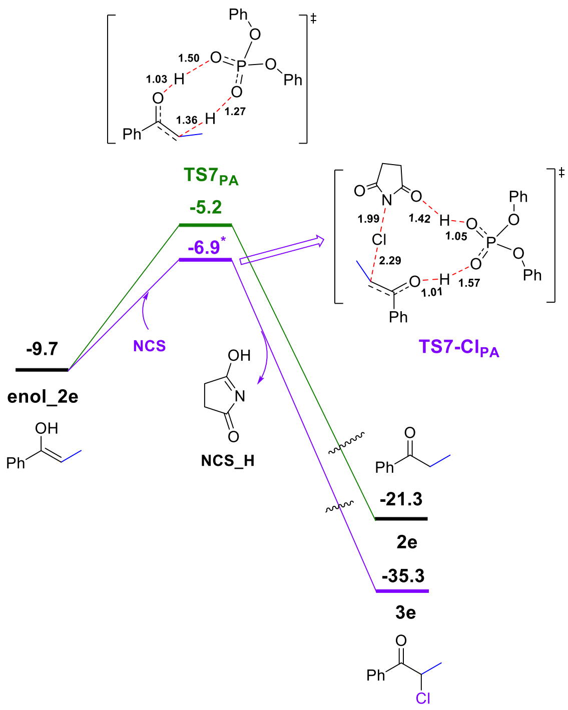

Figure S8. Calculated Gibbs energy profiles (kcal/mol) for the tautomerization (**TS7$_{PA}$**) and chlorination (**TS7-Cl$_{PA}$**) of enol with the presence of acid.

Additional results for the reaction profile of the Ir(III) catalyzed isomerization and isomerization-chlorination of allylic alcohol 1e in mixture solvent

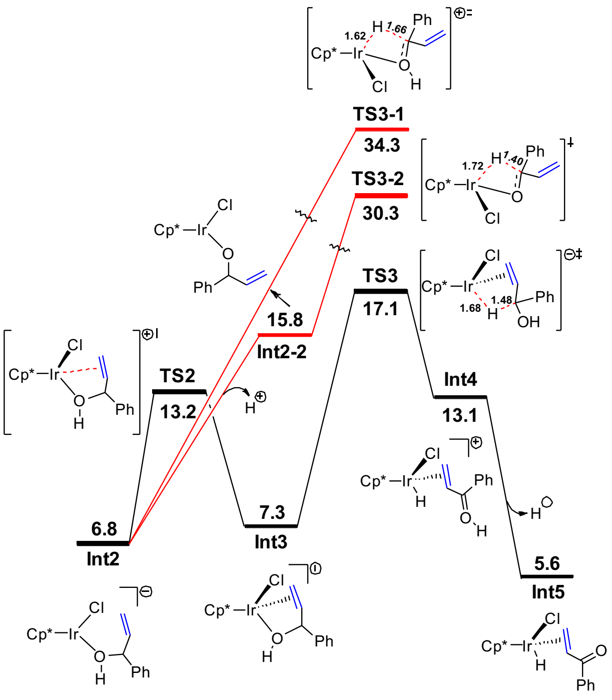

Figure S9. Calculated results of the transition states for the β-hydride elimination (red line) for **1e** in mixed solvent.

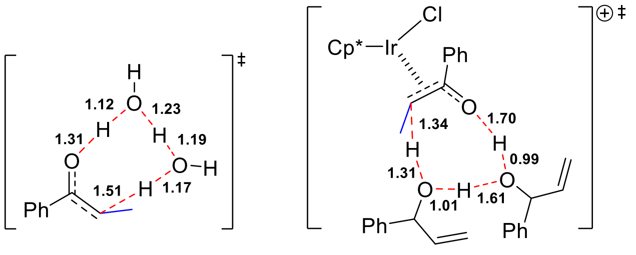

19.2	34.0*	1.4
**TS7-1**	**TS7-2**	**TS7-Cl-1**

Figure S10. Other related transition states.

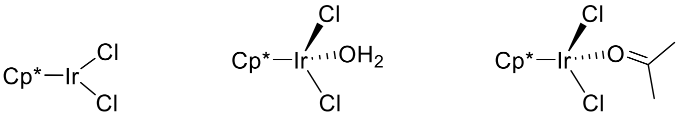

**Int0**	**Int_H$_2$O**	**Int_acetone**
**0.0**	**7.2**	**9.0**

Figure S11. Calculated results of the solvent molecule bound Ir complexes.

Note that **1b-TS6a**, **TS7-Cl$_{PA}$**,and **TS7-2** are marked with an asterisk (*) in Figures S5, S8, and S10. These geometries have one additional imaginary frequency each (<11 cm^{-1}). Many attempts were made to eliminate these frequencies without success. It was therefore replaced by a real frequency of the same magnitude in the RRHO calculations. Experience of similar cases shows that the error bar of this treatment rather small.

Calculated absolute energies and energy corrections.

Stationary point	Thermal correction to Gibbs free energy at 298.15 K (a.u.)	Solvation energy (1,2-ethaneDiol) (a.u.)	Solvation energy (acetone) (a.u.)	Single-point energy B3LYP-D3(BJ)/6-311+G(2d,2p)-LanL2TZ (a.u.)
Int0	0.177731	-0.031727	-0.03876685	-1415.56924
1e	0.131289	-0.0073282	-0.012747807	-424.34247
Int1	0.338956	-0.0263049	-0.03719252	-1839.94537
Int1a	0.333866	-0.0273437	-0.03642263	-1839.93684
TS1	0.330577	-0.0438554	-0.04918539	-1839.9095
Cl^-	-0.015023	-0.1082721	-0.106616512	-460.303727
Int2	0.337494	-0.0927755	-	-1379.44856
Int2-1	0.332171	-	-0.045825	-1839.91606
Int2-2	0.323789	-0.03038793	-	-1379.047812
TS2	0.340167	-0.0903728	-	-1379.44352
TS2-1	0.332064	-	-0.05117642	-1839.9063
Int3	0.342751	-0.0862779	-0.08915502	-1379.4596
TS3	0.335945	-0.0831679	-0.09121115	-1379.44021
TS3-1	0.331783	-0.08479065	-	-1379.406993
TS3-2	0.318755	-0.02925298	-	-1379.02081
Int4	0.33688	-0.0794104	-0.08850632	-1379.45133
Int5	0.324282	-0.024881	-0.03652413	-1379.0701
TS4	0.326127	-0.0231173	-0.03525848	-1379.05332
Int6	0.327014	-0.0156046	-0.02680351	-1379.08527
TS5	0.32527	-0.0190275	-0.02952035	-1379.07031
TS5’	0.321455	-0.024159	-0.0373598	-1379.04478
Int7	0.326234	-0.0239193	-0.03406303	-1379.08384
Int7’	0.326073	-0.0193528	-0.03001527	-1379.07936
Int8	0.338243	-0.0884587	-	-1379.47075
Int8’	0.334482	-0.0856912	-	-1379.44206
enol-2e	0.131419	-0.006742	-0.013395438	-424.357489
TS6	0.322564	-0.0330413	-	-1379.05688
HCl	-0.011159	-	-0.005837941	-460.8368445
TS6-1	0.336929	-	-0.04697915	-1839.932292
Int7a	0.326914	-0.0326893	-	-1379.06791
Int8a	0.335464	-0.0929712	-	-1379.45777
TS7	0.430585	-0.01161317	-0.02710323	-1273.047926
TS7’	0.328942	-0.085525	-	-1379.42921

**TS7-1’**	**0.327128**	**-**	**-0.05685025**	**-1839.889585**
**TS7a**	**0.642132**	**-0.0875532**	**-**	**-2228.18383**
**TS7-1**	**0.169848**	**-0.016515089**	**-**	**-577.2706145**
**TS7-2**	**0.64382**	**-0.0808438**	**-**	**-2228.15455**
**Int8-1’**	**0.336155**	**-**	**-0.09185151**	**-1379.482518**
**NCS**	**0.049112**	**-0.010068983**	**-0.018253663**	**-820.3831089**
**NCS_H**	**0.062016**	**-0.0146046**	**-0.017817015**	**-360.7795937**
**TS7-Cl**	**0.358597**	**-0.0196575**	**-0.03531431**	**-1669.11239**
**TS7-Cl-1**	**0.224604**	**-0.0195048**	**-**	**-1321.22719**
**PA**	**0.165153**	**-**	**-0.02135876**	**-1106.580747**
**$TS7_{PA}$**	**0.313697**	**-**	**-0.02781981**	**-1530.952029**
**$TS7\text{-}Cl_{PA}$**	**0.390505**	**-**	**-0.03887871**	**-2351.369655**
**2e**	**0.132112**	**-0.0058937**	**-0.013518894**	**-424.37639**
**3e**	**0.121325**	**-0.0072839**	**-0.015760102**	**-884.002559**
**1b**	**0.18875**	**-0.008058832**	**-**	**-389.8624591**
**1b-Int1**	**0.393213**	**-0.02640865**	**-**	**-1805.459418**
**1b-TS1**	**0.389646**	**-0.03892499**	**-**	**-1805.435741**
**1b-Int2**	**0.398849**	**-0.0843768**	**-**	**-1344.983328**
**1b-TS2**	**0.394592**	**-0.08647104**	**-**	**-1344.964158**
**1b-Int3**	**0.39566**	**-0.08529072**	**-**	**-1344.969341**
**1b-Int4**	**0.38219**	**-0.01854475**	**-**	**-1344.593817**
**1b-TS3**	**0.385288**	**-0.01816271**	**-**	**-1344.575368**
**1b-Int5**	**0.383752**	**-0.01642682**	**-**	**-1344.600569**
**1b-TS4**	**0.381414**	**-0.01904003**	**-**	**-1344.585082**
**1b-Int6**	**0.381735**	**-0.02329859**	**-**	**-1344.594461**
**1b-TS4’**	**0.379243**	**-0.01863777**	**-**	**-1344.56746**
**1b-Int6’**	**0.38348**	**-0.01773765**	**-**	**-1344.59491**
**1b-Int7**	**0.395099**	**-0.08990164**	**-**	**-1344.981989**
**1b-Int7’**	**0.391609**	**-0.08471962**	**-**	**-1344.954925**
**enol-2b**	**0.187666**	**-0.005755112**	**-**	**-389.8725094**
**1b-TS5**	**0.378673**	**-0.03154026**	**-**	**-1344.569849**
**1b-Int6a**	**0.380522**	**-0.03375327**	**-**	**-1344.577289**
**1b-Int7a**	**0.392974**	**-0.09554218**	**-**	**-1344.968063**
**1b-TS6**	**0.608596**	**-0.0121008**	**-**	**-1169.605751**
**1b-TS6’**	**0.38719**	**-0.08876148**	**-**	**-1344.936633**
**1b-TS6a**	**0.817081**	**-0.08518187**	**-**	**-2124.745749**
**1b-TS6-Cl**	**0.470623**	**-0.01825662**	**-**	**-1600.149305**
**2b**	**0.187688**	**-0.006317016**	**-**	**-389.8880855**
**3b**	**0.176349**	**-0.007281511**	**-**	**-849.5163828**
**Int0-H_2O**	**0.203074**	**-0.027629**	**-**	**-1492.05185**
**Int0-actone**	**0.256668**	**-0.0248193**	**-**	**-1608.82026**

Cartesian coordinates.

Int0

Ir	-0.15382200	0.39496500	-0.00174100
Cl	-2.31817700	1.29491700	-0.02793100
Cl	0.81019300	2.53301000	0.05727800
C	-0.27054300	-1.56544300	0.90888500
C	1.08606200	-1.02404400	1.04990700
C	1.64137300	-0.84583800	-0.26044600
C	0.60871900	-1.20529000	-1.21398100
C	-0.55205700	-1.70294900	-0.48802700
C	-1.18220600	-1.93213600	2.03308800
H	-0.99997200	-1.30527500	2.90803000
H	-2.22672300	-1.80687100	1.74409100
C	1.78312900	-0.75174700	2.34157700
H	2.29038900	-1.65797700	2.69464500
H	1.07691400	-0.43571900	3.11162600
H	2.52758800	0.03685600	2.22340200
C	3.00870500	-0.33045600	-0.57197200
H	3.26351300	0.50678100	0.08118600
H	3.06898500	0.03013600	-1.59986900
H	3.75733200	-1.12017100	-0.44137100
C	0.73857200	-1.14331800	-2.69972900
H	1.15194500	-2.08632200	-3.07816300
H	1.40191600	-0.33176500	-3.00276400
H	-0.23112900	-0.98138100	-3.17309600
C	-1.81709000	-2.22587600	-1.08627900
H	-1.96431300	-1.83901400	-2.09599000
H	-2.68157400	-1.92253300	-0.49286300
H	-1.79412200	-3.32063700	-1.13811100
H	-1.02425800	-2.97792800	2.32328400

1e

C	-2.86570100	0.43059600	-0.20660500
C	-2.48955600	-0.90922400	-0.29675400
C	-1.15183300	-1.27151000	-0.12338800
C	-0.18220600	-0.29979800	0.13290800
C	-0.56539200	1.04466300	0.21845000
C	-1.89990200	1.40722900	0.05377100
H	-3.90575700	0.71444200	-0.33667600
H	-3.23585300	-1.67305300	-0.49433900
H	-0.85932500	-2.31701100	-0.18384900
H	0.19482100	1.79415600	0.41512300
H	-2.18937800	2.45175800	0.12350900
C	1.28254500	-0.67546100	0.30989000
H	1.33970300	-1.77481800	0.40090800
C	2.08281400	-0.26806700	-0.90147500
C	3.09920100	0.59074200	-0.88265400
H	1.75396700	-0.72616000	-1.83238000
H	3.62985900	0.85392300	-1.79233100
H	3.42700400	1.04663700	0.04477200
O	1.84811900	-0.05595400	1.45846800
H	1.22064000	-0.17160800	2.18291400

Int1

Ir	-0.58810300	-0.26989500	0.25609600
Cl	0.71060600	-1.29412900	-1.53605300
Cl	-0.28333100	-2.39787800	1.40675700
C	-1.89447200	1.47697400	-0.33195100
C	-2.16457800	1.08977700	1.04831600
C	-2.68183500	-0.24603700	1.03264900
C	-2.66711800	-0.72361000	-0.34387100
C	-2.22725100	0.36727900	-1.17693200
C	-1.49042100	2.83582800	-0.81038800
H	-0.63499300	2.76945000	-1.48595500
H	-2.32920400	3.30910800	-1.33338600
C	-2.04964200	1.98202600	2.24297600
H	-2.93912700	2.61589800	2.33765600
H	-1.18161800	2.64156400	2.16961200
H	-1.94997500	1.39920500	3.16024100
C	-3.19541100	-1.01610400	2.20137600
H	-2.80823300	-0.62244700	3.14219700
H	-2.90347600	-2.06434400	2.13173900
H	-4.29017100	-0.95142000	2.22379300

C	-3.13580300	-2.06569200	-0.80467200
H	-4.23009600	-2.09958000	-0.86294300
H	-2.79532900	-2.84238500	-0.11626000
H	-2.72943000	-2.29751200	-1.79049300
C	-2.10582500	0.34179300	-2.66397700
H	-1.70226100	-0.61406000	-3.00259100
H	-1.44080600	1.13275500	-3.01427000
H	-3.09053500	0.49039400	-3.12239000
H	-1.21319600	3.48427900	0.02286300
C	4.55009700	4.79947300	0.39002800
C	3.61026200	4.92172400	-0.63365600
C	2.80036000	3.83888700	-0.98023800
C	2.92706500	2.62162100	-0.30584100
C	3.87197200	2.50316700	0.72044100
C	4.68026800	3.58427800	1.06629600
H	5.18102300	5.64217100	0.65654000
H	3.50946400	5.86147200	-1.16915200
H	2.08031400	3.91958600	-1.78564200
H	3.97638500	1.55823800	1.24719100
H	5.41444900	3.47748300	1.85940300
C	2.03013100	1.44381800	-0.64235400
H	2.63958600	0.53680100	-0.72725500
C	1.06307100	1.25731800	0.52000300
C	1.18554100	0.24884300	1.48138300
H	0.54820600	2.17200700	0.79715000
H	0.82832600	0.39145300	2.49518300
H	1.91248300	-0.54458600	1.34255400
O	1.35951800	1.69331700	-1.86624400
H	1.03122300	0.81498000	-2.13764500

Int1a

Ir	-0.83848700	-0.34655700	-0.17025500
Cl	0.89714000	-0.24922600	1.52589200
Cl	-0.42120200	-2.73443000	-0.51488700
C	-2.00400700	1.25658000	-1.08424100
C	-2.69543800	-0.00743000	-1.25545500
C	-2.98030900	-0.52444000	0.06750000
C	-2.43907200	0.39500400	1.04334300
C	-1.84315600	1.51594400	0.32374000
C	-1.52786900	2.12366600	-2.20251400
H	-1.13181500	1.51792900	-3.02065900
H	-0.73619600	2.79426400	-1.87016800
C	-3.09604000	-0.63857500	-2.54958200
H	-4.13027600	-0.38338400	-2.81030000
H	-2.44905600	-0.30336300	-3.36307900
H	-3.01314800	-1.72570100	-2.48769400
C	-3.69112800	-1.80441400	0.35984100
H	-3.43858100	-2.56449200	-0.38049900
H	-3.41170800	-2.19408100	1.33945400
H	-4.77523200	-1.64007000	0.34530300
C	-2.52637900	0.28024300	2.53018900
H	-3.33203700	0.91839900	2.91251700
H	-2.72779500	-0.74761200	2.83563800
H	-1.58522600	0.58471500	2.99136800
C	-1.22024500	2.71094200	0.96797100
H	-0.59714300	2.40217200	1.81046200
H	-0.58206100	3.25018400	0.26644800
H	-1.99158000	3.39860100	1.33475800
H	-2.35894200	2.72401900	-2.59075600
C	2.09363400	0.22234600	-1.52781900
H	2.40988800	-0.01439900	-0.51052800
O	0.78211600	-0.38802900	-1.74623000
H	0.88598500	-1.36470600	-1.75134500
C	1.92551600	1.70750300	-1.67180900
C	2.02242700	2.55130700	-0.64492400
H	1.74793700	2.07056900	-2.68285800
H	1.93535700	3.62553200	-0.78276400
H	2.18131200	2.18567900	0.36503700
C	3.07066300	-0.33455900	-2.53764300
C	4.35927200	-0.69717200	-2.13520800
C	2.71202600	-0.46036400	-3.88602100
C	5.28117900	-1.17329800	-3.06750600
H	4.63622100	-0.60797000	-1.08857400
C	3.63039600	-0.94311500	-4.81627900
H	1.70689500	-0.19221300	-4.19694600
C	4.91850800	-1.29748500	-4.40928800

H	6.27878000	-1.45507900	-2.74464400
H	3.34190000	-1.04395200	-5.85836800
H	5.63390800	-1.67381700	-5.13424300

TS1

Ir	-0.68519900	0.17333700	-0.00823800
Cl	0.87029500	-0.02507600	1.73601200
Cl	-0.13877800	-2.97787500	-1.72888800
C	-2.11043800	1.44643400	-1.00448700
C	-2.47773200	0.09169000	-1.29478900
C	-2.70968800	-0.57721200	-0.02883800
C	-2.55879300	0.39105300	1.05101500
C	-2.17073300	1.63800400	0.45034700
C	-1.73362200	2.49133900	-2.00219500
H	-1.26862800	2.03648500	-2.87836600
H	-1.02239100	3.20206700	-1.57710400
C	-2.56682400	-0.55919000	-2.63522000
H	-3.61650300	-0.75489800	-2.88322200
H	-2.14778600	0.08080100	-3.41340000
H	-2.01766400	-1.50728600	-2.63490600
C	-3.06420600	-2.01753600	0.11046100
H	-2.40189100	-2.62612000	-0.51363700
H	-2.97301500	-2.35214900	1.14442000
H	-4.10207900	-2.16744300	-0.21273200
C	-2.77186700	0.12661200	2.50560000
H	-3.82398500	0.27750000	2.77342200
H	-2.49517900	-0.89844700	2.75760400
H	-2.15873900	0.78926100	3.11826100
C	-1.89101700	2.91850700	1.16417300
H	-1.55821100	2.73177900	2.18583300
H	-1.11190100	3.48928300	0.65488800
H	-2.79809400	3.53383300	1.20033500
H	-2.62118200	3.04588300	-2.32905100
C	2.12737000	0.08285400	-1.38712800
H	2.52976600	-0.20796600	-0.41309800
O	0.74500700	-0.33610700	-1.47592300
H	0.58641600	-1.38368600	-1.61560700
C	2.12060300	1.58286600	-1.51326500
C	2.47219500	2.41707500	-0.53484800
H	1.80879300	1.95836200	-2.48700400
H	2.46845600	3.49390500	-0.67735100
H	2.77352000	2.04655400	0.44020400
C	2.94285900	-0.55206400	-2.49498600
C	4.33013400	-0.64051700	-2.33667000
C	2.35735000	-0.99660100	-3.68348400
C	5.12459300	-1.16722400	-3.35259800
H	4.78551400	-0.29477700	-1.41234300
C	3.15324900	-1.53125400	-4.69706200
H	1.28061300	-0.95899200	-3.79940800
C	4.53631800	-1.61478200	-4.53741000
H	6.19980200	-1.23622600	-3.21671000
H	2.68774200	-1.89184500	-5.60933500
H	5.15248300	-2.03454800	-5.32689700

Int2

Ir	-0.10893100	1.14848200	0.42451600
Cl	0.68218700	3.28785500	-0.09360600
C	-1.87625400	0.21763700	1.30731100
C	-1.17768000	-0.81974200	0.60139400
C	-1.05490700	-0.39875300	-0.77198000
C	-1.75095100	0.87565400	-0.93724900
C	-2.25480500	1.26009600	0.34950000
C	-2.21697800	0.21854500	2.75715000
H	-1.50827900	-0.37927900	3.33074200
H	-2.22633200	1.23282900	3.16094300
C	-0.73983000	-2.13135900	1.15869400
H	-1.52747300	-2.87311600	0.98031000
H	0.17348100	-2.49138000	0.68547000
H	-0.55907100	-2.07720700	2.23077100
C	-0.40908000	-1.16683100	-1.87353300
H	0.29389300	-1.90757700	-1.49041000
H	0.11648500	-0.50742400	-2.56715800
H	-1.18096200	-1.70076000	-2.44154900
C	-1.92835200	1.60993800	-2.22442000
H	-2.82509800	1.24153800	-2.73582900
H	-1.07374000	1.46113400	-2.88665100

H	-2.04268900	2.68151900	-2.05824800
C	-3.05216400	2.48199100	0.66145100
H	-2.85859700	2.83615600	1.67543800
H	-4.12098200	2.25227900	0.57982000
H	-2.82134400	3.29274700	-0.03028600
H	-3.21889800	-0.20605200	2.89572500
C	1.10891400	-2.73264800	4.46807800
C	1.89755600	-3.22086600	3.42564300
C	2.41489000	-2.34565100	2.46969700
C	2.14643900	-0.97508900	2.54800400
C	1.37371900	-0.48462200	3.60881300
C	0.85343800	-1.36171400	4.56071200
H	0.70881600	-3.41254300	5.21322600
H	2.11348800	-4.28203100	3.35636800
H	3.03534600	-2.72774200	1.66549900
H	1.18537600	0.58033300	3.69120100
H	0.26274000	-0.97329900	5.38472700
C	2.75305700	-0.02607600	1.54166800
H	3.64433800	0.44855600	1.96760600
C	3.07829100	-0.61571300	0.20532500
C	4.28879600	-0.55656600	-0.34825200
H	2.25056400	-1.10014600	-0.30858800
H	4.49244500	-0.98917600	-1.32221900
H	5.12545300	-0.08307700	0.15912100
O	1.79962300	1.09364400	1.35156200
H	2.26369200	1.89235800	1.03368300

TS2

Ir	0.05079600	1.04027600	0.30126900
Cl	1.09548800	3.02803900	-0.37957100
C	-1.75909500	0.27011400	1.24348600
C	-1.15252300	-0.82498700	0.54421800
C	-1.04601700	-0.44482600	-0.85092100
C	-1.62990400	0.87017500	-1.03009500
C	-2.06503800	1.32362600	0.27214100
C	-2.06538700	0.32306800	2.70119400
H	-1.36367100	-0.28457200	3.27345400
H	-2.02548200	1.34675300	3.07795700
C	-0.78340300	-2.14841900	1.12857300
H	-1.63098100	-2.83560800	1.02080800
H	0.07107800	-2.59616900	0.62014200
H	-0.53879900	-2.07221300	2.18761900
C	-0.45948700	-1.27646100	-1.93968700
H	0.22760400	-2.02813000	-1.54911100
H	0.07152300	-0.65928200	-2.66690100
H	-1.26496200	-1.80009800	-2.46876400
C	-1.80215100	1.59717200	-2.32314200
H	-2.78440700	1.36735100	-2.75092400
H	-1.03888300	1.30654500	-3.04665600
H	-1.73224300	2.67646200	-2.18074100
C	-2.75389000	2.61084800	0.56988400
H	-2.52540800	2.96252600	1.57727000
H	-3.83872500	2.46529900	0.49859100
H	-2.46500000	3.38773000	-0.13871200
H	-3.07768500	-0.06043100	2.87664300
C	1.22323200	-2.94480900	4.61172100
C	2.03904000	-3.32289600	3.54446300
C	2.47414500	-2.36993100	2.62345800
C	2.09972200	-1.02828500	2.76247800
C	1.30716300	-0.64751200	3.85148100
C	0.86518300	-1.60407000	4.76675900
H	0.88135500	-3.68682100	5.32581000
H	2.33801500	-4.35938900	3.42706600
H	3.11364700	-2.67612600	1.80113200
H	1.03400000	0.39344500	3.97703900
H	0.25003800	-1.29895900	5.60758900
C	2.57294500	-0.00858700	1.75372200
H	3.54531100	0.40437600	2.04503700
C	2.62176800	-0.52649000	0.33304600
C	3.26778300	0.09759200	-0.65652700
H	2.06931800	-1.44000400	0.13742000
H	3.23518900	-0.27304500	-1.67564600
H	3.84383200	1.00384500	-0.49068700
O	1.63681100	1.13756600	1.80503200
H	2.10778500	1.95216200	1.54177100

Int3

Ir	0.27757900	0.89043400	0.25243600
Cl	0.86613000	3.19935000	-0.09080100
C	-1.74755900	0.32159400	1.19575100
C	-1.13346100	-0.81988300	0.55315100
C	-0.98109800	-0.50389500	-0.85564200
C	-1.43413400	0.85263700	-1.06201600
C	-1.92030800	1.35628700	0.22430000
C	-2.08130500	0.41019900	2.64582700
H	-1.41122500	-0.20834100	3.24456100
H	-2.02510800	1.43852300	3.00611100
C	-0.88941100	-2.15087300	1.18865700
H	-1.80684900	-2.74921800	1.14275400
H	-0.10722900	-2.70823200	0.67109600
H	-0.59637100	-2.05538100	2.23478900
C	-0.51175600	-1.44335100	-1.91630000
H	0.23320600	-2.14627000	-1.53815800
H	-0.08895800	-0.90979500	-2.76873700
H	-1.36286700	-2.03058500	-2.27980300
C	-1.52800700	1.57977600	-2.36185700
H	-2.52876400	1.44603500	-2.78868900
H	-0.79781200	1.20708300	-3.08157800
H	-1.35035600	2.64702300	-2.22296700
C	-2.54730400	2.69311100	0.43792900
H	-2.55806500	2.96736000	1.49352600
H	-3.58295200	2.67472400	0.07986100
H	-2.00732500	3.46952200	-0.10676800
H	-3.10531400	0.05259800	2.80734300
C	1.42655200	-3.06796300	4.61547000
C	2.25711400	-3.33716900	3.52569400
C	2.59117700	-2.31923400	2.63381800
C	2.09864200	-1.02280000	2.82534300
C	1.28729600	-0.74923900	3.92988900
C	0.94973600	-1.77264100	4.81877200
H	1.16422100	-3.86063800	5.30836200
H	2.64659300	-4.33797400	3.37051300
H	3.24493800	-2.53897100	1.79364600
H	0.92381400	0.25799600	4.09196100
H	0.32056100	-1.55294400	5.67555300
C	2.44244300	0.04645300	1.81487800
H	3.48641900	0.36693300	1.90975800
C	2.09575700	-0.37189000	0.38650900
C	2.28102800	0.50077700	-0.68769500
H	1.86177200	-1.41747100	0.22261800
H	2.14914300	0.14134800	-1.70217100
H	2.84592500	1.42102200	-0.58561600
O	1.57407400	1.20924000	2.04848400
H	2.02904800	2.05158000	1.85962600

TS3

Ir	-0.05286800	0.56922900	-0.10796500
Cl	0.58013100	2.74614700	-0.92501900
C	-1.66989100	1.41047700	1.18629500
C	-1.19316100	0.23188900	1.84647600
C	-1.38793900	-0.89340000	0.96095800
C	-2.06747500	-0.41936200	-0.22898300
C	-2.21437100	1.00626700	-0.11388500
C	-1.71954300	2.79894800	1.73166600
H	-1.42133500	3.52240900	0.97040800
H	-2.73934000	3.03450900	2.05580700
C	-0.56212200	0.14925000	3.19795500
H	-0.22414400	1.12720600	3.54222600
H	0.29152600	-0.53245200	3.19060700
H	-1.28905900	-0.23285300	3.92359200
C	-1.06191500	-2.31459000	1.29364200
H	-0.08610200	-2.39309100	1.77861300
H	-1.05386500	-2.94409100	0.40205700
H	-1.81369200	-2.72204300	1.97874100
C	-2.55522300	-1.26946000	-1.35575400
H	-3.55958000	-1.64333900	-1.12575200
H	-1.91137400	-2.13627600	-1.51765300
H	-2.61423900	-0.70304500	-2.28607800
C	-2.90293300	1.92467500	-1.06734000
H	-2.99756800	1.47813000	-2.05799800
H	-2.34979900	2.86017100	-1.16759600
H	-3.90907300	2.15341600	-0.69750600

H	-1.05454700	2.91723300	2.58825800
C	5.59300400	2.18946000	-1.07517300
C	4.89697000	2.46998400	0.10317600
C	3.80135600	1.69158900	0.46284200
C	3.39905600	0.62858400	-0.35679300
C	4.09489100	0.34932800	-1.53965100
C	5.19299200	1.13153300	-1.89293000
H	6.44332600	2.80068700	-1.35895600
H	5.19721600	3.30259000	0.73004400
H	3.23211800	1.93291300	1.35705700
H	3.78998900	-0.47933300	-2.16948300
H	5.73530700	0.91378200	-2.80685400
C	2.28660400	-0.26600000	0.07867600
H	1.43033300	0.79917800	0.65095600
C	1.36186900	-0.89179500	-0.87179300
C	0.86386500	-0.17718100	-1.98214000
H	1.07145500	-1.91512200	-0.65720800
H	0.17653900	-0.68631400	-2.64803600
H	1.40062900	0.66816600	-2.39438000
O	2.51619800	-1.05388800	1.16883900
H	3.29077200	-0.71398500	1.64257500

Int4

Ir	0.06648200	0.45502200	0.10805000
Cl	0.70342700	1.85778700	-1.75640300
C	-1.80753800	1.47366100	0.60132200
C	-1.41631400	0.70531700	1.76441500
C	-1.42611900	-0.68676000	1.38886300
C	-1.93150100	-0.79286100	0.03207700
C	-2.15208500	0.52681700	-0.45893300
C	-2.03622900	2.94913300	0.52531100
H	-1.71294400	3.34066700	-0.44103400
H	-3.10182800	3.17235300	0.64995900
C	-1.13017600	1.24051700	3.13141300
H	-0.79697800	2.27814400	3.08961500
H	-0.36150600	0.65491800	3.63907500
H	-2.04077300	1.20163300	3.74011300
C	-1.13266100	-1.83616200	2.29880500
H	-0.40343300	-1.56401000	3.06415100
H	-0.75146300	-2.69988800	1.74949000
H	-2.04954600	-2.15273300	2.80919300
C	-2.20001700	-2.06887000	-0.69688700
H	-3.22493500	-2.40297900	-0.49740700
H	-1.53128400	-2.86928200	-0.37220400
H	-2.09295200	-1.94582400	-1.77585200
C	-2.67727500	0.91671200	-1.79949200
H	-2.68875800	0.07221500	-2.48950200
H	-2.06189800	1.70595100	-2.23710900
H	-3.70214800	1.29184900	-1.69970500
H	-1.48416900	3.47752600	1.30401200
C	5.12930800	2.28905800	-2.04761400
C	4.64457600	2.88287500	-0.87783600
C	3.87809200	2.13930000	0.00907300
C	3.57198500	0.79624400	-0.28256700
C	4.05965600	0.20502000	-1.46033700
C	4.84435400	0.95221000	-2.33222000
H	5.72893300	2.87188700	-2.73897800
H	4.85292800	3.92644300	-0.66925800
H	3.45455800	2.61587400	0.88911100
H	3.86073600	-0.83999400	-1.66568600
H	5.23456500	0.49167000	-3.23321700
C	2.79427400	0.01810900	0.67274300
H	0.90858300	1.58428200	0.83343400
C	1.77510100	-0.93217000	0.36996600
C	1.17312200	-1.08185100	-0.92082000
H	1.55317000	-1.63490100	1.16698100
H	0.59528900	-1.98435600	-1.08913000
H	1.62084600	-0.64570700	-1.80372000
O	3.08773400	0.12464700	1.96010400
H	3.83776300	0.72975300	2.08426900

Int5

Ir	-0.07832300	0.20506300	-0.11026700
Cl	0.48208200	2.06506900	-1.55490200
C	-1.69414900	1.22179600	1.00219200
C	-1.25848300	0.07846500	1.76602500

C	-1.54773400	-1.09640900	0.97922800
C	-2.29109200	-0.68647300	-0.20289600
C	-2.37056500	0.72475700	-0.19558100
C	-1.65960800	2.66040200	1.41379100
H	-1.37529000	3.28846800	0.56625800
H	-2.63986200	2.98908700	1.77960600
C	-0.67849000	0.10028200	3.14633800
H	-0.18562100	1.05255700	3.34824300
H	0.06438300	-0.68704000	3.28382900
H	-1.46887000	-0.04088000	3.89340500
C	-1.33849700	-2.51551500	1.40680800
H	-0.55545500	-2.59168400	2.16379400
H	-1.05867500	-3.15344200	0.56391000
H	-2.25922800	-2.92606200	1.83826100
C	-2.84897600	-1.62295300	-1.22722600
H	-3.80956700	-2.03607900	-0.89553900
H	-2.17718000	-2.46819400	-1.40178400
H	-3.00983500	-1.12079400	-2.18280800
C	-2.99696200	1.60979700	-1.22256700
H	-3.37569300	1.03660000	-2.07059300
H	-2.25970000	2.32708800	-1.59689000
H	-3.83233300	2.16893500	-0.78623700
H	-0.92747300	2.82520300	2.20658000
C	4.59918900	-1.12016300	3.63991500
C	3.29539700	-1.58358400	3.45365800
C	2.69959100	-1.50055900	2.19667500
C	3.40111300	-0.95718700	1.11146300
C	4.70949400	-0.49594700	1.30841300
C	5.30484800	-0.57544600	2.56396000
H	5.06310500	-1.18240500	4.61989200
H	2.74349200	-2.00663500	4.28800500
H	1.68225700	-1.84813000	2.06520600
H	5.23301800	-0.07534300	0.45699100
H	6.31827000	-0.21277000	2.70706700
C	2.81485900	-0.83344500	-0.26467900
H	1.14016400	0.80695300	0.67658800
C	1.42144700	-1.34212800	-0.48877900
C	0.74170400	-1.04448600	-1.69132400
H	1.14361600	-2.22881900	0.07406200
H	-0.02747300	-1.71792200	-2.05810600
H	1.23702400	-0.42510600	-2.42970800
O	3.46178300	-0.33795600	-1.17518800

TS4

Ir	3.15940400	1.18820800	0.10497200
Cl	3.99891000	3.16748300	-1.06277100
C	1.50096000	2.38777200	0.96415100
C	1.74954700	1.26117900	1.81742200
C	1.47432900	0.07476700	1.05053000
C	0.84906500	0.49681700	-0.22024000
C	0.87990900	1.89271700	-0.27629500
C	1.60123500	3.83524400	1.32644100
H	2.04276200	4.40206900	0.50394700
H	0.60802000	4.24590700	1.54577500
C	2.17988800	1.30501500	3.25074000
H	2.77190700	2.19729000	3.46019600
H	2.78175700	0.43267600	3.51215500
H	1.29937700	1.31953800	3.90461100
C	1.46095500	-1.32412100	1.58273900
H	2.23517200	-1.46793100	2.33953000
H	1.62544200	-2.05828100	0.79008800
H	0.49161100	-1.55559000	2.04222300
C	0.28993900	-0.44017300	-1.24231100
H	0.23078200	0.02863800	-2.22636300
H	-0.72082900	-0.75783800	-0.95740700
H	0.89745500	-1.34330600	-1.33696900
C	0.45579600	2.78856300	-1.39222300
H	0.09542700	2.22115700	-2.25213000
H	1.30447400	3.40178100	-1.71377500
H	-0.34546800	3.46082100	-1.06431000
H	2.23225200	3.97999800	2.20536200
C	9.24311800	2.69702200	-1.55245300
C	8.46736900	2.41015900	-0.42735100
C	7.24146800	1.76478300	-0.57094500
C	6.77939300	1.40797300	-1.84467300
C	7.55628700	1.71125400	-2.97062600

C	8.78474800	2.34793500	-2.82564500
H	10.20034000	3.19743900	-1.43786200
H	8.81434800	2.69761600	0.56054700
H	6.62333900	1.58016200	0.30023200
H	7.17064900	1.43923200	-3.94703700
H	9.38488700	2.57631600	-3.70135600
C	5.47620000	0.71797300	-2.05671400
H	4.37132600	1.35762500	1.08714800
C	4.84314500	0.02462700	-0.87584200
C	3.62556400	-0.63238800	-1.04939800
H	5.51342800	-0.29260900	-0.08663300
H	3.37638500	-1.49108100	-0.43708700
H	3.14923600	-0.55478100	-2.02208000
O	4.97354300	0.62369300	-3.16741800

Int6

Ir	0.12950000	0.77912000	0.01785100
Cl	0.67627400	2.72746400	-1.33793100
C	-1.93807500	1.35945500	0.45783500
C	-1.51397100	0.37855100	1.42915600
C	-1.26772800	-0.85234500	0.71509400
C	-1.70550100	-0.67986900	-0.66237300
C	-2.06456400	0.67683100	-0.83079100
C	-2.38408600	2.76401300	0.71818300
H	-2.00879500	3.43295200	-0.05956700
H	-3.47875600	2.82901500	0.73575400
C	-1.42435000	0.56887000	2.91166300
H	-1.23368900	1.61283500	3.16575700
H	-0.62081700	-0.03290900	3.34072600
H	-2.36485700	0.26778500	3.38879100
C	-0.86507100	-2.16174400	1.31690300
H	-0.38992500	-2.02036200	2.28984200
H	-0.16432900	-2.68605400	0.66355500
H	-1.74605900	-2.79882400	1.46188200
C	-1.80817200	-1.78013800	-1.66501200
H	-2.73553100	-2.34174800	-1.49454400
H	-0.97574800	-2.48144400	-1.57428500
H	-1.82582800	-1.40082500	-2.68751800
C	-2.50996200	1.34203800	-2.09206900
H	-2.31534200	0.71336700	-2.96247500
H	-1.97192600	2.28376700	-2.23105100
H	-3.58382800	1.55997600	-2.05479600
H	-2.00655400	3.12605700	1.67648700
C	0.35446300	-1.03454600	-5.83837600
C	0.49062900	0.12439000	-5.07159400
C	0.86580400	0.04090000	-3.73118800
C	1.09208100	-1.21374700	-3.14558500
C	0.94781400	-2.37472700	-3.91968700
C	0.58812800	-2.28610200	-5.26068800
H	0.06615500	-0.96423600	-6.88326200
H	0.30315300	1.09756000	-5.51516400
H	0.94118900	0.94642200	-3.13899000
H	1.11637200	-3.33421300	-3.44299900
H	0.48484000	-3.18815600	-5.85635300
C	1.41018000	-1.37018200	-1.69469700
H	0.81376100	1.69189400	1.09182700
C	1.86228500	-0.17614200	-0.93460600
C	2.01283900	-0.23916800	0.47161900
H	2.38161300	0.59505000	-1.49203400
H	2.72174600	0.41810600	0.95938300
H	1.81798400	-1.18271100	0.97083900
O	1.27674800	-2.46903600	-1.14805900

TS5

Ir	-0.20701000	0.53382600	-0.14942400
Cl	0.61261600	2.62400700	-1.10233400
C	-2.04431700	1.42962900	0.72514400
C	-1.61957600	0.38474000	1.59734900
C	-1.63132000	-0.86010800	0.84443200
C	-2.19439800	-0.58385800	-0.45787300
C	-2.36783600	0.83081400	-0.57312200
C	-2.19967900	2.88050900	1.04947700
H	-1.74621600	3.49600900	0.26915800
H	-3.26123500	3.14266000	1.13164500
C	-1.24028800	0.52540600	3.03848800
H	-0.86943400	1.52852000	3.25580400

H	-0.46185000	-0.18849500	3.31547800
H	-2.10980300	0.33734100	3.67990400
C	-1.31577100	-2.22564700	1.37028600
H	-0.67267600	-2.16775400	2.25143500
H	-0.79725900	-2.81796700	0.61276200
H	-2.23250600	-2.75397300	1.65949900
C	-2.56667700	-1.62386200	-1.46036400
H	-3.54225800	-2.04546900	-1.18744400
H	-1.84267100	-2.44111200	-1.47729500
H	-2.64473700	-1.21268100	-2.46695000
C	-2.90578800	1.58057000	-1.74971300
H	-2.77619000	1.00697900	-2.66979100
H	-2.37364000	2.52692500	-1.86991200
H	-3.97398900	1.79592000	-1.62477600
H	-1.71269200	3.13075300	1.99377700
C	-1.25158300	-1.34473400	-5.89968000
C	-0.93226600	-0.16895000	-5.21602500
C	-0.29496200	-0.22841600	-3.97743900
C	0.01521100	-1.47373500	-3.40818800
C	-0.31363800	-2.64842600	-4.09667700
C	-0.93775900	-2.58572000	-5.33944100
H	-1.74477000	-1.29383100	-6.86598900
H	-1.18109900	0.79615900	-5.64688900
H	-0.07462600	0.68841800	-3.44025800
H	-0.07723500	-3.59867400	-3.63006300
H	-1.18423400	-3.50034600	-5.87039900
C	0.62519000	-1.59802300	-2.04600100
H	1.15714500	0.57688600	0.77363000
C	1.22903600	-0.38150300	-1.47752600
C	1.83424300	-0.39681900	-0.16557200
H	1.59837200	0.35417600	-2.18152600
H	2.72718800	0.20973100	-0.03427400
H	1.82729600	-1.33985400	0.37252400
O	0.57326200	-2.67026400	-1.43573900

Int7

Ir	-0.90867300	0.45537700	-0.07673400
Cl	-0.46188700	-0.03024800	-2.33371200
C	-2.74015000	1.49592500	0.41924200
C	-1.91664500	1.37756900	1.62433100
C	-1.76110700	-0.01754200	1.88537900
C	-2.50478900	-0.74947000	0.85279100
C	-3.18381500	0.18385500	0.01406100
C	-3.13871300	2.78660200	-0.21619100
H	-3.34835700	2.65603300	-1.27920000
H	-4.04197700	3.17943700	0.26699300
C	-1.39986800	2.49288200	2.47318600
H	-2.03947100	2.62576500	3.35425000
H	-1.38978100	3.43560800	1.92234000
H	-0.38014000	2.28105400	2.80502100
C	-1.11026300	-0.62722200	3.08162200
H	-0.29263900	-0.00403600	3.44585200
H	-0.70618600	-1.61537100	2.85846900
H	-1.85219200	-0.73396300	3.88297100
C	-2.55257600	-2.23934800	0.76075400
H	-3.28107100	-2.64004800	1.47634300
H	-1.57619000	-2.67035400	0.99478700
H	-2.83837400	-2.56645500	-0.24008500
C	-4.04603800	-0.13482300	-1.16486400
H	-3.76590400	-1.09285300	-1.60743000
H	-3.94068800	0.62214700	-1.94474800
H	-5.10329400	-0.18519600	-0.87880400
H	-2.34853900	3.53302800	-0.11388600
C	1.14960400	0.96548200	0.12841800
H	1.66511000	0.44330000	-0.68062900
C	1.53635300	0.42449800	1.45388700
O	1.60137600	1.13022500	2.46730500
C	1.79766000	-1.05525000	1.58105900
C	1.53875500	-1.96992700	0.54850600
C	2.27168900	-1.53319400	2.80997300
C	1.72867800	-3.33626300	0.75560400
H	1.17356200	-1.62623400	-0.41250600
C	2.47105100	-2.89608800	3.01347800
H	2.46306000	-0.81005900	3.59524700
C	2.19279700	-3.80287500	1.98737500
H	1.51781300	-4.03547200	-0.04810700

H	2.83880000	-3.25393100	3.97072900
H	2.34167300	-4.86714400	2.14516300
C	1.34220200	2.47576100	-0.00989700
H	0.81132600	3.02015300	0.77252300
H	2.40475000	2.73720200	0.07804800
H	0.98919100	2.82188500	-0.98487100

Int8

Ir	-0.73224600	0.77189700	0.00245200
Cl	-0.33274900	0.44474000	2.28348200
C	-2.75077200	1.51640600	-0.49675700
C	-1.94616000	1.48124700	-1.71624700
C	-1.54149300	0.12774900	-1.93750700
C	-2.08409100	-0.67610400	-0.84362300
C	-2.87952200	0.18215500	0.01148900
C	-3.35030500	2.75076800	0.08603400
H	-3.60267800	2.61412600	1.13776000
H	-4.26980500	3.00276200	-0.45620100
C	-1.71261300	2.64536800	-2.61803000
H	-2.55407600	2.73681400	-3.31473200
H	-1.63769600	3.57731500	-2.05627400
H	-0.79970000	2.52678300	-3.20164700
C	-0.80361900	-0.40023400	-3.12262700
H	-0.16485800	0.36524300	-3.56510600
H	-0.19215700	-1.26716400	-2.86324400
H	-1.51672300	-0.71890700	-3.89173400
C	-1.92609300	-2.14993400	-0.69073100
H	-2.74443100	-2.65849400	-1.21558800
H	-0.98468400	-2.49870400	-1.11875100
H	-1.96068600	-2.44614600	0.35850500
C	-3.64637000	-0.24566100	1.21833800
H	-3.16525400	-1.09111100	1.71220600
H	-3.71775400	0.56159500	1.94836300
H	-4.66106700	-0.54407200	0.93241900
H	-2.66725200	3.59849600	0.00169200
H	1.76202400	-0.64226300	-1.51091100
C	2.16386300	-0.24715300	-0.57047600
C	1.47895800	1.02981400	-0.11367600
H	3.24462100	-0.09595800	-0.68833700
H	1.73139900	1.31530900	0.90301300
H	2.02280600	-1.02674600	0.17993600
C	1.31402700	2.11556200	-1.00374400
O	1.51631000	1.96296000	-2.31758500
C	0.97070200	3.48651900	-0.61004500
C	0.58582300	3.78605700	0.71347000
C	1.00756400	4.51556400	-1.56820000
C	0.22739000	5.08384500	1.05638700
H	0.54815100	3.00972200	1.46883200
C	0.65134700	5.81351700	-1.21385900
H	1.32204800	4.29420600	-2.58056100
C	0.25472900	6.09940600	0.09456900
H	-0.06885600	5.30584500	2.07602400
H	0.68801300	6.60299400	-1.95691800
H	-0.02334500	7.11210600	0.36797900
H	1.90700400	1.09247700	-2.49221100

enol-2e

C	-4.81429000	10.21356800	5.57955600
C	-3.92416700	9.26317700	5.07880500
C	-4.11600000	8.71804700	3.81062900
C	-5.20753900	9.11229700	3.02116100
C	-6.08551100	10.08499900	3.52610900
C	-5.89526000	10.62325900	4.79553400
H	-4.66300900	10.63916900	6.56700800
H	-3.07670200	8.94285600	5.67783400
H	-3.42606300	7.97903900	3.42101700
H	-6.90601500	10.43835600	2.91058700
H	-6.58360400	11.37620800	5.16799900
C	-5.41022800	8.50860700	1.68805400
H	-7.19637500	6.83626400	-0.28898100
C	-6.59414700	8.43916300	1.05175300
C	-6.81050200	7.86466800	-0.31927100
H	-7.46868600	8.80158200	1.57942900
H	-7.54148000	8.45890200	-0.87801400
H	-5.89491800	7.85462500	-0.92432300
O	-4.24232600	8.00099100	1.17181200

H	-4.45074400	7.48099200	0.38557600

TS7

C	-3.53910500	10.75130200	5.50183900
C	-2.67936900	10.36958100	4.46889100
C	-3.19068800	9.77155900	3.32073000
C	-4.56991100	9.54582300	3.18159400
C	-5.42139100	9.91513300	4.23296400
C	-4.91020000	10.51814800	5.38135400
H	-3.14300800	11.21991900	6.39789500
H	-1.61004100	10.53727700	4.56176700
H	-2.54643800	9.46948800	2.50349000
H	-6.48633500	9.72440700	4.16330700
H	-5.58258400	10.80031000	6.18605800
C	-5.05198100	8.93385300	1.89988900
H	-6.21534000	7.62491300	-0.17291600
C	-6.41941200	8.94828500	1.54154900
C	-6.96064300	7.90001900	0.57679200
H	-7.11680900	9.24756200	2.32064400
H	-7.24954700	6.98117400	1.10309600
H	-7.84405800	8.26757200	0.04462800
O	-4.12780900	8.51349800	1.11647300
H	-4.30045500	8.82795700	-0.19197200
O	-4.44781400	9.30800300	-1.15971400
H	-5.29120700	10.12523800	-0.88924200
O	-6.23570000	10.71469100	-0.40218600
H	-6.35040800	10.07093000	0.55824200
C	-6.13410600	12.11177200	-0.08515100
H	-6.54075600	12.22523300	0.93163700
C	-3.19944100	9.88877500	-1.59265100
H	-2.95181100	10.72236700	-0.92838600
C	-4.68043300	12.55926100	-0.04507100
C	-4.05831100	13.13227900	-1.15940000
C	-3.92696800	12.32408400	1.11080200
C	-2.70102100	13.45516000	-1.12003100
H	-4.63537800	13.30275400	-2.06173800
C	-2.56855000	12.63439400	1.14893600
H	-4.40059500	11.88198700	1.98052500
C	-1.95158300	13.20099800	0.03045400
H	-2.22643600	13.89600400	-1.99161300
H	-2.00024800	12.43257500	2.05153400
H	-0.89409200	13.44642600	0.05669500
C	-2.09456400	8.85919100	-1.47510000
C	-1.09851700	9.01761900	-0.50879300
C	-2.07804400	7.73325700	-2.30456600
C	-0.08997100	8.06307800	-0.37271200
H	-1.12108400	9.89057500	0.13780500
C	-1.07536600	6.77597900	-2.16583700
H	-2.85425900	7.61708700	-3.05459000
C	-0.07840300	6.94044500	-1.20060700
H	0.68086400	8.19240300	0.38121500
H	-1.06832000	5.90209200	-2.81056800
H	0.70364000	6.19439300	-1.09474300
C	-6.97789000	12.92838600	-1.02718200
C	-7.66475800	12.43121500	-2.05262900
H	-6.98888800	13.99687200	-0.81655700
H	-8.25671100	13.07146700	-2.69883400
H	-7.65784600	11.36712400	-2.26162600
C	-3.35372400	10.42108200	-2.98935800
C	-4.44582200	10.29579900	-3.74097800
H	-2.47576800	10.94173700	-3.36609700
H	-4.49025500	10.70884800	-4.74350100
H	-5.32525400	9.77845700	-3.37318100

TS6

Ir	-0.43435200	0.28638800	-1.13408300
Cl	0.91649900	1.30332600	0.55202600
C	-2.40613300	1.11463300	-1.57457500
C	-2.14468800	-0.03822000	-2.43722800
C	-1.96217600	-1.18293900	-1.59455300
C	-2.08035200	-0.72927800	-0.20898300
C	-2.41719800	0.67833000	-0.21055500
C	-2.66024600	2.50516200	-2.05915700
H	-2.36943800	3.24381200	-1.31050100
H	-3.72569400	2.64233400	-2.28132600
C	-2.12746600	-0.03108400	-3.93172600

H	-3.13508400	-0.20351900	-4.32933700
H	-1.77285600	0.92620400	-4.31996900
H	-1.46942100	-0.81190200	-4.31753900
C	-1.66449700	-2.57315200	-2.05426300
H	-0.93758200	-2.56020200	-2.86984300
H	-1.24076800	-3.17232200	-1.24642400
H	-2.57603300	-3.06993800	-2.40647800
C	-1.96939900	-1.58721200	1.00790700
H	-2.95357300	-1.99126000	1.27597400
H	-1.28988700	-2.42509600	0.84138900
H	-1.59191700	-1.01022000	1.85358800
C	-2.64967800	1.51697500	1.00485700
H	-1.91366600	1.28841300	1.77871600
H	-2.55268300	2.57869100	0.77258500
H	-3.65360900	1.34505500	1.41006700
H	-2.09735000	2.71306100	-2.97156400
C	6.01981800	-1.25321900	-1.22264300
C	4.92492200	-1.42195800	-0.37117600
C	3.67814000	-0.91105400	-0.72218800
C	3.51187000	-0.21045000	-1.92673900
C	4.61283900	-0.05482300	-2.78080500
C	5.85984300	-0.57109900	-2.42970300
H	6.99105100	-1.65519500	-0.94869700
H	5.04556200	-1.95092500	0.56992400
H	2.82272600	-1.02169400	-0.06581400
H	4.48176100	0.45245500	-3.73106800
H	6.70376700	-0.44909300	-3.10265600
C	2.16882600	0.31253100	-2.27251100
H	2.45578000	3.57627400	-2.51398600
C	1.91377400	1.53175500	-2.81434500
C	2.87120400	2.67142000	-2.97629600
H	0.87894600	1.71252400	-3.10708000
H	3.06076000	2.91518500	-4.03103600
H	3.83091500	2.46673300	-2.49794300
O	1.16380400	-0.54260200	-1.99804800

Int7a

Ir	-0.33215700	0.16669900	-0.25226300
Cl	0.14376800	-0.03881500	2.06497500
C	-1.31429100	0.14277500	-2.22570600
C	-0.74006900	-1.14173200	-1.93912400
C	-1.38610100	-1.68012700	-0.74081400
C	-2.34829400	-0.72773100	-0.28676400
C	-2.25841300	0.42751600	-1.16213900
C	-1.04154400	1.02577300	-3.40024800
H	-0.74140200	2.02724700	-3.07859700
H	-1.93557700	1.11316700	-4.02785900
C	0.28105600	-1.86144400	-2.75682000
H	-0.21804600	-2.57459400	-3.42461700
H	0.87395100	-1.17409900	-3.35964000
H	0.97194400	-2.41872000	-2.12220800
C	-1.09697400	-3.00544000	-0.11443300
H	-0.07483000	-3.32437700	-0.32730900
H	-1.20996000	-2.95168700	0.96964000
H	-1.78059200	-3.77130200	-0.50021300
C	-3.22625900	-0.86395300	0.91522600
H	-4.13056900	-1.43311200	0.67002600
H	-2.69895400	-1.37412800	1.72344300
H	-3.53093800	0.11410800	1.29220100
C	-3.06901800	1.67712400	-1.05526900
H	-3.30292200	1.90966400	-0.01485000
H	-2.53097800	2.52596300	-1.48215600
H	-4.01356400	1.56099600	-1.60116700
H	-0.23306300	0.62394400	-4.01074500
C	3.91753700	-0.96659600	-3.68903400
C	3.86282800	-1.02108600	-2.29408300
C	3.13472500	-0.07249500	-1.58048600
C	2.45266900	0.96158200	-2.24598800
C	2.51276500	1.00451500	-3.64865700
C	3.23889700	0.05007200	-4.36257700
H	4.48030100	-1.71026700	-4.24525700
H	4.38875700	-1.80701800	-1.75965900
H	3.07964800	-0.11655400	-0.49883300
H	1.96564700	1.76862100	-4.18731100
H	3.26502600	0.09640900	-5.44753500
C	1.64865600	1.91997000	-1.44602200

H	2.26208500	5.03654900	-2.48318300
C	1.41559800	3.21693000	-1.73900600
C	2.00520400	4.03676600	-2.85109200
H	0.78301800	3.74147800	-1.02587900
H	1.30600100	4.18542000	-3.68694100
H	2.91482600	3.58953700	-3.25923300
O	1.18211600	1.40679200	-0.27284200

Int8a

Ir	-0.50880200	0.25665900	-0.20783500
Cl	-0.37183700	0.98544100	2.00698600
C	-1.40232300	0.04535900	-2.21468400
C	-0.74879000	-1.17970800	-1.88071000
C	-1.35044600	-1.68628600	-0.64794000
C	-2.40004600	-0.78567100	-0.24147100
C	-2.41028700	0.30919800	-1.19193200
C	-1.13815900	0.91356800	-3.39864900
H	-1.13935600	1.97068000	-3.12221300
H	-1.91990100	0.76181900	-4.15154500
C	0.31402900	-1.87419000	-2.66201200
H	-0.14151700	-2.67485100	-3.25684600
H	0.83190300	-1.19498700	-3.33787900
H	1.06048300	-2.32541600	-2.00718000
C	-0.96009900	-2.95027300	0.03743800
H	0.10682200	-3.15069900	-0.07680900
H	-1.19626600	-2.91828400	1.10160500
H	-1.50961100	-3.78807100	-0.40923000
C	-3.31372300	-0.94834300	0.92821400
H	-4.22179700	-1.47767100	0.61913400
H	-2.83722200	-1.51932100	1.72599300
H	-3.60216500	0.01908800	1.34158400
C	-3.33790300	1.47457700	-1.17660600
H	-3.66372900	1.71392600	-0.16369500
H	-2.87196000	2.36025400	-1.61226000
H	-4.22728000	1.23414900	-1.77246900
H	-0.17474400	0.68132000	-3.85052200
C	3.75903800	-0.97278500	-3.91143200
C	3.78736000	-1.08475700	-2.51942700
C	3.16230200	-0.12606800	-1.72579800
C	2.50784400	0.96870400	-2.31659000
C	2.47931100	1.07078400	-3.71790200
C	3.10265400	0.10620500	-4.50811900
H	4.24777500	-1.72009300	-4.52793300
H	4.30365300	-1.91679400	-2.05134000
H	3.18918100	-0.21190900	-0.64517000
H	1.95878400	1.89809200	-4.18685100
H	3.07445700	0.19655000	-5.58915400
C	1.86718000	1.98304900	-1.47113600
H	2.91694300	5.02026200	-2.18031200
C	1.77669600	3.31029300	-1.60787600
C	2.44982000	4.14939900	-2.65111500
H	1.19158100	3.84548000	-0.86045800
H	1.72579300	4.53636800	-3.37854400
H	3.21934300	3.59795100	-3.19336200
O	1.28751600	1.39134000	-0.30699700
H	1.36113200	1.95107800	0.48854800

TS7a

C	-6.00386400	4.89104800	-0.84608400
C	-6.81572000	5.99066700	-0.55712800
C	-6.25822800	7.26461100	-0.44972000
C	-4.87798600	7.44541100	-0.61801800
C	-4.06306900	6.33673400	-0.89734300
C	-4.62929700	5.06749800	-1.01849100
H	-6.44246700	3.90316000	-0.94442000
H	-7.88425400	5.85706200	-0.42071100
H	-6.89164800	8.11934300	-0.23612800
H	-2.99987200	6.47836500	-1.05894600
H	-3.99695400	4.21973000	-1.26327400
C	-4.26135700	8.79436500	-0.52870500
C	-4.77853100	9.90189700	-1.19305700
O	-3.21453200	8.93806500	0.25096800
H	-3.22709700	10.30086300	1.27587600
O	-3.50441900	11.10946800	1.77821800
H	-4.97225600	11.09042700	1.47387400
O	-5.88382000	10.82533700	1.05356100

H	-5.55969600	10.36975500	0.06958800
C	-6.80003100	11.98629100	0.90561600
H	-6.22521200	12.80583600	0.46400000
C	-2.70829300	12.20850700	1.22898700
H	-2.84327200	12.21519500	0.14047900
C	-7.90236200	11.55084800	-0.02363200
C	-8.59570200	10.35571900	0.20982600
C	-8.23790800	12.33673200	-1.12872900
C	-9.60054200	9.94654300	-0.66420700
H	-8.34342300	9.74516500	1.07191900
C	-9.25258100	11.93203300	-1.99770000
H	-7.70410300	13.26522700	-1.31154600
C	-9.93025800	10.73427700	-1.77008900
H	-10.13165400	9.01775300	-0.48100800
H	-9.50711800	12.54814100	-2.85391300
H	-10.71415400	10.41589700	-2.44952300
C	-3.23551800	13.49639900	1.80791800
C	-3.62053700	14.54241800	0.96397900
C	-3.32313800	13.66901000	3.19542600
C	-4.07142600	15.75281600	1.49618000
H	-3.54919700	14.41717400	-0.11338700
C	-3.77378200	14.87471600	3.72809400
H	-3.03904400	12.85678700	3.85740700
C	-4.14476500	15.92146200	2.87932400
H	-4.35801400	16.56200400	0.83208400
H	-3.83387800	15.00147900	4.80443400
H	-4.48959400	16.86270000	3.29525500
C	-7.25438200	12.36180400	2.28174000
C	-6.91059900	13.51016400	2.86272800
H	-7.90135600	11.64421800	2.78121500
H	-7.26397400	13.77071100	3.85505800
H	-6.27055100	14.23237300	2.36599800
C	-1.25983000	11.93881800	1.53837800
C	-0.42677000	11.37827100	0.66175700
H	-0.92504800	12.19920400	2.54008300
H	0.60616600	11.16075100	0.91271800
H	-0.74910000	11.10751100	-0.34011700
C	-5.70717200	9.84109800	-2.38984800
H	-5.13317700	9.89500400	-3.32228500
H	-6.29214900	8.92142900	-2.41512300
H	-6.41142400	10.67750100	-2.38920500
H	-4.10532400	10.76198400	-1.17546600
Ir	-2.42276200	7.59625000	1.65036000
Cl	-0.62691500	7.41663300	0.16601600
C	-2.38970000	5.91249500	3.00452000
C	-3.75937600	6.43455400	2.93408600
C	-3.75432700	7.78529900	3.40652500
C	-2.38411000	8.12424800	3.73412100
C	-1.54812700	6.94195600	3.52825900
C	-1.97940200	4.52894600	2.62285500
H	-0.92351200	4.49052100	2.35321300
H	-2.14785900	3.84440400	3.46232800
C	-4.95830700	5.65233700	2.52675400
H	-4.71848400	4.92001200	1.75677000
H	-5.74381400	6.29933000	2.13765700
H	-5.35203800	5.11643300	3.39911400
C	-4.94779400	8.67486300	3.54142400
H	-5.64890200	8.52504100	2.71775000
H	-4.65838900	9.72552300	3.54896700
H	-5.47338300	8.45751200	4.47817800
C	-1.91500700	9.43208600	4.27357000
H	-2.00093000	9.42607400	5.36720100
H	-2.51185400	10.25354400	3.87649000
H	-0.87017200	9.61214700	4.01641500
C	-0.08316000	6.85035400	3.80016700
H	0.40923500	7.81004100	3.63442000
H	0.39329400	6.11843700	3.14705000
H	0.08677600	6.55169800	4.84076300
H	-2.56021900	4.17014600	1.77095100

PA

H	1.01241700	0.80265500	4.32718400
O	3.09787600	-1.56395700	3.35539600
O	3.51683200	-0.53688800	5.54863000
P	2.91146700	-0.15141200	4.10951500
O	1.34093900	-0.09968500	4.44722000

O	3.44098300	1.05752000	3.44072900
C	4.87538000	-0.83823100	5.71752800
C	5.86990200	0.02740300	5.26682300
C	5.17851900	-2.01129200	6.40145300
C	7.20348100	-0.30760500	5.50651100
H	5.59615600	0.93269500	4.73808400
C	6.51589200	-2.32704200	6.63999200
H	4.37107000	-2.65418800	6.73334100
C	7.53041300	-1.47866700	6.19200000
H	7.98822300	0.35643400	5.15748300
H	6.76278900	-3.23967500	7.17351400
H	8.57019200	-1.72887700	6.37712300
C	2.74139800	-1.76218600	2.01542000
C	3.17316300	-0.90024600	1.00887300
C	1.98440800	-2.89589300	1.73406600
C	2.82341400	-1.18996500	-0.31108600
H	3.75816300	-0.02509500	1.26352700
C	1.64993700	-3.17426100	0.40910900
H	1.67221100	-3.53866100	2.54951600
C	2.06626400	-2.32218100	-0.61572700
H	3.15166500	-0.52500600	-1.10403700
H	1.06100500	-4.05711600	0.18050700
H	1.80302400	-2.54042800	-1.64583600

TS7$_{PA}$

C	0.15912000	5.78274800	6.07601500
C	1.43337300	5.25932800	6.30681300
C	1.96472300	4.30973600	5.44095500
C	1.21473900	3.86014500	4.34185300
C	-0.06467300	4.39156100	4.11367900
C	-0.58667300	5.35136400	4.97588600
H	-0.25312200	6.52756700	6.75026900
H	2.01244900	5.58529300	7.16501000
H	2.94696200	3.89015500	5.61506400
H	-0.63787200	4.07144500	3.25041300
H	-1.57221200	5.76615500	4.78954400
C	1.79293200	2.82333700	3.47023100
H	1.25519500	1.84182600	0.64444700
C	1.00997200	1.86423500	2.80144400
C	1.47357500	1.20304000	1.50780400
H	-0.05979300	2.05660000	2.85500800
H	2.54202700	0.98687300	1.51868100
H	0.95175600	0.25295400	1.36483100
O	3.10268200	2.78180500	3.49509000
H	3.47335500	1.83707900	3.31763200
H	1.21413200	0.87205000	3.71052600
O	3.13958600	-2.02786500	4.06415200
O	3.62888200	-0.44296800	5.90549500
P	3.03402200	-0.45237400	4.38585600
O	1.53604200	-0.12329000	4.43322300
O	3.88345600	0.39832200	3.46378500
C	3.79252600	0.77681100	6.55853400
C	2.83361400	1.17397800	7.48819500
C	4.92262700	1.55243700	6.30398400
C	3.02689400	2.35994700	8.19755300
H	1.95994200	0.55101100	7.64177300
C	5.10347000	2.73791200	7.01756600
H	5.63382700	1.22023300	5.55715400
C	4.16231600	3.14041400	7.96892400
H	2.28620500	2.67479700	8.92597500
H	5.98318000	3.34583800	6.83009800
H	4.31240600	4.05969900	8.52683700
C	4.35137300	-2.72340700	4.08630600
C	4.40016800	-3.88021900	4.85914700
C	5.44307100	-2.30713900	3.32607300
C	5.57051000	-4.63847000	4.87165000
H	3.52765800	-4.16555100	5.43604100
C	6.60971200	-3.07251200	3.35520900
H	5.37044200	-1.39876100	2.74074900
C	6.67815200	-4.23655900	4.12245200
H	5.61603600	-5.54210100	5.47181400
H	7.46727600	-2.75567600	2.76929400
H	7.58864700	-4.82740700	4.13646900

TS7-Cl$_{PA}$

H	-2.03494400	2.48984000	3.90640700

C	-2.49855600	2.65011000	2.92994800
C	-1.63238500	3.55863000	2.09743700
H	-2.59837400	1.67081000	2.44342000
H	-0.57604100	3.31635800	2.08366800
H	-3.49754400	3.05189700	3.09477100
C	-2.08106500	4.27687300	0.98237900
O	-3.34036900	4.55081600	0.72487000
C	-1.14735500	4.86995700	0.00452000
C	0.12784700	4.33167600	-0.22952500
C	-1.54984600	6.01150700	-0.70851000
C	0.98566200	4.92953000	-1.14799600
H	0.44203600	3.43133600	0.28652300
C	-0.68688000	6.60985400	-1.62107500
H	-2.53434500	6.42289500	-0.52126500
C	0.58261000	6.07171900	-1.84292900
H	1.96664900	4.50059400	-1.32653100
H	-1.00296100	7.49873600	-2.15809100
H	1.25436600	6.53794600	-2.55735300
C	-1.66436500	8.01579200	4.81499200
C	-1.05646000	8.89553600	5.89939200
C	0.04808500	8.02724200	6.51347900
C	0.03473100	6.73604900	5.68738300
N	-0.96527100	6.86661700	4.71657300
H	-0.68872000	9.81711900	5.43873500
H	-0.14910700	7.76609300	7.55578500
Cl	-1.30845900	5.42276100	3.39603200
O	0.74012700	5.76651900	5.84238500
O	-2.66382900	8.34714900	4.15821100
H	1.04520400	8.47169900	6.46312100
H	-1.84711500	9.16502300	6.60264300
H	-4.00078100	4.40485100	1.47523500
H	-3.55801300	7.46979300	3.48523700
C	-9.16294900	4.09356800	6.38103200
C	-8.39109000	3.40833200	5.44217700
H	-9.61293100	6.01791000	7.24642800
C	-9.01488600	5.47470400	6.52128700
C	-7.47136300	4.08666800	4.64086700
C	-7.33075000	5.46388800	4.80555700
C	-8.09522300	6.16636800	5.73458500
C	-2.86733500	5.18961000	6.72261500
C	-2.43225900	5.78950000	7.90150600
C	-3.02365900	6.97466400	8.34887900
C	-4.05206900	7.55479600	7.60506600
C	-4.50056400	6.96299000	6.42208900
C	-3.89976900	5.77972400	5.99478400
H	-1.62235100	5.33345000	8.46182600
O	-6.44270700	6.22821900	4.04396900
O	-4.28051400	5.10069700	4.84576700
P	-5.01141900	5.67558100	3.52300700
O	-4.31559200	6.94570100	2.98514000
O	-5.13233400	4.53913600	2.55674700
H	-7.96250100	7.23875000	5.82579400
H	-2.40531900	4.28773600	6.33986500
H	-9.87669900	3.55638400	6.99734200
H	-8.50479900	2.33523600	5.32300900
H	-4.51756300	8.47549800	7.94327900
H	-2.68689000	7.43985600	9.26999700
H	-5.29400300	7.41816500	5.84386000
H	-6.87855700	3.56593200	3.89858400

2e

C	-3.46549000	-3.43379200	-3.39692200
C	-4.09117900	-3.15985400	-2.17730300
C	-3.32661100	-2.97464100	-1.03047000
C	-1.92743500	-3.06073100	-1.08638200
C	-1.30749200	-3.33604800	-2.31364200
C	-2.07412100	-3.52156800	-3.46330000
H	-4.06134200	-3.57844700	-4.29326200
H	-5.17358200	-3.09155100	-2.12506400
H	-3.78540200	-2.76083100	-0.07135200
H	-0.22722800	-3.40682500	-2.37846700
H	-1.58658100	-3.73417900	-4.40980400
C	-1.15887400	-2.85114900	0.18282200
H	0.63585100	-3.92798800	-0.25527900
C	0.36121100	-2.94108100	0.14079600
C	1.00630600	-2.70154800	1.50255000

H	0.72807400	-2.21743600	-0.59944300
H	0.65601200	-3.43479000	2.23306300
H	2.09532200	-2.77259300	1.43055800
O	-1.74239200	-2.61274600	1.23001000
H	0.74539900	-1.71262000	1.88715700

NCS

C	-1.83300200	-1.07231400	0.27833600
C	-0.30920000	-1.09996700	0.27837800
C	0.14572400	0.37126000	0.27835000
C	-1.12756700	1.20858100	0.27819000
N	-2.19218600	0.28825500	0.27825600
H	0.02425600	-1.65634600	1.15820200
H	0.73518200	0.64241700	-0.60137600
Cl	-3.81995700	0.79194500	0.27813900
O	-1.24009800	2.40791300	0.27804600
O	-2.60287200	-1.99884100	0.27837600
H	0.73498900	0.64248800	1.15818500
H	0.02428100	-1.65638000	-0.60141700

TS7-Cl

C	-5.57755900	10.62991200	5.54196300
C	-4.32452700	10.67716900	4.92520000
C	-4.12987700	10.05907800	3.69558300
C	-5.19373700	9.39157700	3.06110200
C	-6.44892800	9.34396300	3.69309800
C	-6.63715700	9.95992600	4.92574300
H	-5.72807500	11.11295700	6.50261900
H	-3.50255800	11.20117600	5.40226500
H	-3.16764000	10.09478500	3.19946000
H	-7.27046100	8.80575700	3.23433500
H	-7.60764800	9.91397100	5.40910300
C	-4.95883900	8.75936600	1.75772400
H	-6.39873300	6.42886400	0.43789800
C	-6.00820500	8.50280900	0.82686900
C	-5.95031800	7.28887300	-0.07437700
H	-6.99937900	8.74126300	1.19702700
H	-6.51411600	7.46381000	-0.99350500
H	-4.92924000	7.01374200	-0.34191300
O	-3.70788300	8.52132000	1.48374600
H	-3.42387600	8.53068500	0.47716600
C	-5.67116600	12.25729900	-2.80990800
C	-4.72093500	13.19060000	-3.58395500
C	-3.36538900	13.03038200	-2.88549000
C	-3.62023500	11.95294600	-1.83288700
N	-4.93361700	11.62193200	-1.82081300
H	-4.69987800	12.86811600	-4.62874200
H	-3.01900000	13.93993600	-2.38519000
Cl	-5.58566100	10.17924800	-0.41526200
O	-2.73097700	11.45993300	-1.12448300
O	-6.85213400	12.09436000	-3.03824100
H	-2.56383800	12.68648900	-3.54344200
H	-5.12228900	14.20702100	-3.56153200
H	-2.76105900	9.88079000	-0.84360400
O	-2.73352100	8.87430000	-0.78958300
C	-3.21812200	8.34174000	-2.02763700
H	-4.31211800	8.43014400	-2.05404400
C	-2.68132300	9.12015800	-3.21718800
C	-2.85220900	6.88393500	-2.11182500
C	-1.31111300	9.36946700	-3.34198500
C	-3.55938400	9.59756200	-4.19317800
C	-2.10578200	6.23149200	-1.22182600
H	-3.24840200	6.37232900	-2.98695800
C	-0.82659400	10.09382100	-4.42849300
H	-0.63617200	9.00462400	-2.57453300
C	-3.07388100	10.31065900	-5.29144200
H	-4.62672400	9.42766200	-4.08398200
H	-1.87685100	5.17764900	-1.34355500
H	-1.69807800	6.73725400	-0.35344300
C	-1.70659000	10.56345500	-5.40847600
H	0.23753500	10.29249100	-4.51429100
H	-3.76402500	10.67327900	-6.04747500
H	-1.32672500	11.12378500	-6.25763500

NCS-H

C	1.14282800	7.67053200	1.89619400

C	0.30324300	8.79841200	1.35207500
C	-1.00318500	8.58053400	2.12573300
C	-0.70783800	7.34515500	3.00609800
N	0.62203000	6.88802600	2.77572500
H	0.78743000	9.75991700	1.54949700
H	-1.86066800	8.36753800	1.48245700
O	-1.48175200	6.83438300	3.77838800
O	2.38527800	7.53391700	1.44102500
H	-1.27750200	9.42275200	2.76584500
H	0.20267300	8.70496100	0.26617700
H	2.77106500	6.76486300	1.89525300

3e

H	-0.21275300	2.76370000	4.39402000
C	0.37935300	3.17009400	3.57185900
C	-0.52585900	3.76251500	2.51079900
H	1.03766600	3.95340100	3.96059000
H	-1.16252900	4.54677500	2.92474500
H	1.00516900	2.38563800	3.14521900
C	0.25384700	4.29339700	1.29498100
O	1.42971700	3.99370800	1.16950500
C	-0.43322100	5.18496800	0.31674900
C	-1.77909600	5.56423600	0.43280100
C	0.32825900	5.67481700	-0.75630500
C	-2.34902600	6.42003400	-0.50751300
H	-2.39323000	5.18096700	1.23914800
C	-0.24242200	6.52854400	-1.69331900
H	1.36632800	5.37002400	-0.82815500
C	-1.58320300	6.90360700	-1.56954000
H	-3.39185800	6.70607800	-0.41342100
H	0.35351400	6.90315500	-2.51978900
H	-2.03055500	7.57012700	-2.30088900
Cl	-1.69527500	2.48720100	1.89632100

TS5’

Ir	-1.01425000	0.55688500	0.29378100
Cl	-1.19121800	1.02847600	2.67178800
C	-2.79347900	1.66526000	-0.37249500
C	-1.89277100	1.57744900	-1.48407900
C	-1.69159500	0.17515300	-1.77838900
C	-2.58688500	-0.59421500	-0.92200000
C	-3.24572900	0.30652700	-0.04970100
C	-3.31807300	2.90243300	0.28393200
H	-3.32690600	2.78074900	1.36971900
H	-4.34044200	3.11688200	-0.05121900
C	-1.26444500	2.72397000	-2.21115400
H	-1.88340300	3.01825900	-3.06751300
H	-1.15375500	3.59254700	-1.55950300
H	-0.27455800	2.46200300	-2.59204500
C	-0.87859900	-0.37888000	-2.90709100
H	-0.02212300	0.26217600	-3.12971100
H	-0.49619200	-1.37422800	-2.66646400
H	-1.47765400	-0.46533500	-3.82211600
C	-2.74808900	-2.08237600	-0.96335700
H	-3.48271200	-2.37079300	-1.72513200
H	-1.80547300	-2.57674600	-1.21051200
H	-3.08616500	-2.47122000	-0.00129400
C	-4.23227200	-0.01105100	1.02754000
H	-3.92051600	0.44512800	1.97125000
H	-5.22576300	0.37221000	0.76647100
H	-4.31494500	-1.08773300	1.18751300
H	-2.69549300	3.76885500	0.05245700
C	1.35378400	-4.55300700	3.22773100
C	1.85725000	-3.56742000	4.08117700
C	1.96895300	-2.25411900	3.63759100
C	1.57078600	-1.90731500	2.33868700
C	1.06580700	-2.90164900	1.48808300
C	0.96162800	-4.21911900	1.93050900
H	1.26620900	-5.57873100	3.57343300
H	2.15971300	-3.82577900	5.09132300
H	2.35574800	-1.47005600	4.27872100
H	0.75367200	-2.65794600	0.47804500
H	0.57330200	-4.98380300	1.26476300
C	1.71824800	-0.47933900	1.92550900
H	-0.12043500	-0.74287500	0.80092100
C	1.13544500	-0.03590600	0.58037600

C	0.92966200	1.38496300	0.35957600
H	1.50622200	-0.62461700	-0.25936700
H	1.23128900	1.78995500	-0.60317100
H	1.11526100	2.02972700	1.21230500
O	2.32267300	0.32158200	2.61314500

Int7'

Ir	0.21686500	0.33308300	1.84169000
Cl	1.35173900	-1.08670000	0.20240800
C	-0.96745000	1.58185300	0.28037600
C	-0.50265600	2.46804200	1.26723900
C	-0.97874400	1.99905400	2.57666300
C	-1.81614500	0.84836700	2.35383000
C	-1.71620500	0.50786300	0.94831600
C	-0.67811800	1.59695100	-1.18592100
H	-0.18726400	0.66427200	-1.48131500
H	-1.60558000	1.69631800	-1.76178000
C	0.38519000	3.65822100	1.08339100
H	-0.17111200	4.59113300	1.23620600
H	0.81997300	3.68106900	0.08265400
H	1.20857800	3.64137400	1.80355300
C	-0.79797500	2.73837700	3.86538400
H	0.20220700	3.17499600	3.92746700
H	-0.92421900	2.07396500	4.72326900
H	-1.52620100	3.55413700	3.96016500
C	-2.63941500	0.13332200	3.37677100
H	-3.65977900	0.53478000	3.39410000
H	-2.21649800	0.24562300	4.37747100
H	-2.69871300	-0.93494900	3.15712900
C	-2.44736700	-0.58384700	0.23246200
H	-2.72455100	-1.38680500	0.91895500
H	-1.81312000	-1.01707900	-0.54396100
H	-3.36415500	-0.20431300	-0.23651800
H	-0.01966500	2.42426600	-1.45754100
C	6.68286500	0.33866300	4.78079600
C	6.00509100	1.39168300	4.15839300
C	4.71883200	1.19794700	3.67013900
C	4.08908600	-0.05173200	3.80597000
C	4.77775000	-1.10419000	4.43003600
C	6.06956400	-0.90823400	4.91240800
H	7.68935800	0.49005600	5.15918800
H	6.48500800	2.35955300	4.05224900
H	4.18003200	1.99793400	3.17571900
H	4.31102900	-2.07734700	4.52841500
H	6.59786100	-1.72762100	5.38937000
C	2.72460000	-0.22959900	3.27362200
H	2.30517700	-2.13980400	2.57215600
C	1.95152200	-1.50810700	3.39823000
C	0.44780900	-1.23679700	3.20292400
H	2.18867600	-2.02600900	4.33465800
H	-0.03889200	-2.14258400	2.83381200
H	-0.01390000	-0.96660700	4.16089000
O	2.19229800	0.72127000	2.66376100

Int8'

Ir	-0.37810800	-0.56544900	0.38714600
Cl	1.19239900	-0.65416300	2.10003800
C	-1.60560200	1.33953900	0.40392500
C	-2.11983500	0.53589000	1.44549300
C	-2.49189400	-0.73817200	0.85396200
C	-2.34004900	-0.65318100	-0.59607000
C	-1.72283800	0.60446400	-0.87070000
C	-1.01210700	2.70213900	0.53297000
H	-0.16747900	2.83292300	-0.14744100
H	-1.76281400	3.46002400	0.27853400
C	-2.16821500	0.86100300	2.90286000
H	-1.44600100	1.63679100	3.15999600
H	-1.93645100	-0.01596200	3.51071200
H	-3.16629900	1.21581900	3.18316500
C	-3.12148500	-1.87021000	1.59581500
H	-2.69097800	-1.97656300	2.59311100
H	-3.00632000	-2.81681700	1.06539400
H	-4.19525900	-1.67798500	1.71105800
C	-2.87508100	-1.63340800	-1.58991800
H	-3.93867600	-1.43750000	-1.76830200
H	-2.79213800	-2.66339200	-1.23623400

H	-2.36458200	-1.55152300	-2.55147300
C	-1.39792500	1.17147500	-2.21408700
H	-1.25606000	0.38391500	-2.95668100
H	-0.48700500	1.77304800	-2.18106800
H	-2.21274300	1.81883700	-2.55935100
H	-0.66406900	2.89340000	1.54847100
C	-0.27649900	-3.93047000	-0.97312900
H	-0.23512200	-2.43317600	-2.43823800
C	0.45314300	-2.82477100	-1.67618200
C	0.95773300	-1.72940400	-0.74238600
H	1.27763000	-3.28372600	-2.23632100
H	1.74425900	-2.13257300	-0.09703000
O	-0.65215900	-3.74068200	0.24465700
H	-0.44640900	-2.78409100	0.52222400
H	1.43758600	-0.95359800	-1.35521600
C	-0.60559200	-5.19664900	-1.56882100
C	-0.26779200	-5.46756800	-2.91498100
C	-1.28940600	-6.17559800	-0.80812700
C	-0.61447100	-6.68320600	-3.48455100
H	0.25953200	-4.73022900	-3.50863800
C	-1.62473200	-7.38976900	-1.38540100
H	-1.54002400	-5.96904900	0.22560500
C	-1.28959500	-7.64328100	-2.72142800
H	-0.35767200	-6.89118700	-4.51721800
H	-2.14366200	-8.14283500	-0.80278300
H	-1.55300700	-8.59613400	-3.16953800

TS7’

Ir	-0.31774700	-0.40247200	0.49781200
Cl	1.35458100	0.09970300	2.04051000
C	-1.71550400	1.31837100	0.42869800
C	-2.08399100	0.49197100	1.52431200
C	-2.37156200	-0.83881500	0.99065600
C	-2.28176800	-0.78707500	-0.45821800
C	-1.81750400	0.52091000	-0.80361900
C	-1.29931400	2.74993900	0.48909500
H	-0.54521200	2.97742700	-0.26709200
H	-2.16572700	3.39584300	0.30381600
C	-2.11929900	0.87634300	2.96559000
H	-1.43406800	1.69890900	3.17238500
H	-1.83516100	0.03889800	3.60459300
H	-3.13216800	1.19057600	3.24201800
C	-2.81479700	-2.01539100	1.79144700
H	-2.40772100	-1.98241000	2.80306300
H	-2.50007800	-2.95016600	1.32589200
H	-3.90922600	-2.01466900	1.86570200
C	-2.67842900	-1.87050400	-1.40784300
H	-3.71743200	-1.72161900	-1.72275100
H	-2.60776800	-2.85527400	-0.94655600
H	-2.05963900	-1.86418100	-2.30775800
C	-1.58255100	1.04150100	-2.18224100
H	-1.35296700	0.23564900	-2.88190000
H	-0.76340300	1.76293100	-2.20418900
H	-2.48429600	1.55150000	-2.54227200
H	-0.88658100	3.00296400	1.46604700
C	0.12945000	-3.77866300	-1.00749200
H	0.45711900	-2.22988800	-2.44646800
C	0.98224400	-2.66216500	-1.58638400
C	1.22110000	-1.63564600	-0.48685100
H	1.92156600	-3.07659400	-1.96670000
H	2.02719300	-1.92503300	0.18949400
O	-0.31307200	-3.55383100	0.14385500
H	0.11892300	-2.28206500	0.27990000
H	1.52173100	-0.67322800	-0.92956800
C	-0.18439700	-5.00479100	-1.71773100
C	0.28321100	-5.21956000	-3.02894200
C	-0.97161900	-5.98804100	-1.08407500
C	-0.03812700	-6.39577300	-3.69516700
H	0.89447700	-4.47345700	-3.52474400
C	-1.28578400	-7.16157800	-1.75479400
H	-1.31481800	-5.81620100	-0.07018700
C	-0.82042700	-7.36475200	-3.05913900
H	0.32098000	-6.56314100	-4.70469800
H	-1.88670500	-7.92177800	-1.26756600
H	-1.06556700	-8.28451000	-3.58065700

1b

**C**	**1.46097300**	**-0.40718000**	**0.51231600**
**H**	**2.08563300**	**-1.10933500**	**1.09513800**
**C**	**2.19829000**	**-0.07533500**	**-0.75623300**
**C**	**2.53997100**	**1.15290900**	**-1.14496000**
**H**	**2.44103300**	**-0.93376100**	**-1.38309100**
**H**	**2.29000100**	**1.98810000**	**-0.49377800**
**O**	**1.26176300**	**0.79148800**	**1.25408400**
**H**	**0.75358800**	**0.56463400**	**2.04222900**
**C**	**0.12428400**	**-1.09766700**	**0.21072700**
**H**	**0.27447000**	**-2.00921800**	**-0.37673100**
**H**	**-0.38864600**	**-1.38029400**	**1.13822700**
**H**	**-0.52106400**	**-0.41993100**	**-0.35522500**
**C**	**3.27911300**	**1.47446100**	**-2.41183300**
**H**	**3.41404800**	**0.56359900**	**-3.00917300**
**H**	**2.67743600**	**2.16266300**	**-3.02415100**
**C**	**4.64809000**	**2.12569300**	**-2.15175100**
**H**	**4.51050600**	**3.02581200**	**-1.53707500**
**H**	**5.26166600**	**1.43993800**	**-1.55290800**
**C**	**5.39265400**	**2.49563200**	**-3.43771200**
**H**	**4.77052300**	**3.17917800**	**-4.03121900**
**H**	**5.52288500**	**1.59409900**	**-4.05142000**
**C**	**6.75618300**	**3.13916200**	**-3.17481900**
**H**	**7.26784500**	**3.39436600**	**-4.10844700**
**H**	**6.65089800**	**4.05947500**	**-2.58938300**
**H**	**7.40839300**	**2.46318400**	**-2.61047500**

1b-Int1

**Ir**	**-0.90117200**	**-0.91040800**	**-0.12775200**
**Cl**	**0.38891600**	**-2.44338200**	**1.24271500**
**Cl**	**-1.12752600**	**-2.61679000**	**-1.87063400**
**C**	**-1.27259600**	**1.18039000**	**0.38708700**
**C**	**-2.16818400**	**0.77432700**	**-0.68972300**
**C**	**-2.98405500**	**-0.30297300**	**-0.18893300**
**C**	**-2.56361900**	**-0.60449800**	**1.16619000**
**C**	**-1.52948100**	**0.35822500**	**1.53471900**
**C**	**-0.26784800**	**2.28165600**	**0.29055600**
**H**	**0.14566600**	**2.34215700**	**-0.71790900**
**H**	**0.56297700**	**2.11982200**	**0.97664600**
**C**	**-2.27824100**	**1.41486100**	**-2.03573000**
**H**	**-3.00327200**	**2.23775700**	**-2.01909600**
**H**	**-1.31648300**	**1.81931000**	**-2.35813200**
**H**	**-2.60160300**	**0.69009100**	**-2.78546300**
**C**	**-4.05220400**	**-1.01872900**	**-0.94712000**
**H**	**-3.82970100**	**-1.04092000**	**-2.01435000**
**H**	**-4.14369400**	**-2.05207300**	**-0.61047300**
**H**	**-5.01416500**	**-0.51423300**	**-0.79599100**
**C**	**-3.16186800**	**-1.62119700**	**2.08332300**
**H**	**-3.94120900**	**-1.16542300**	**2.70624800**
**H**	**-3.60766700**	**-2.44179600**	**1.51872300**
**H**	**-2.39638400**	**-2.04631800**	**2.73483900**
**C**	**-0.87559400**	**0.43147900**	**2.87474300**
**H**	**-0.51309500**	**-0.55517800**	**3.17367800**
**H**	**-0.02324100**	**1.10962700**	**2.86361400**
**H**	**-1.58993400**	**0.78813200**	**3.62577100**
**H**	**-0.73836700**	**3.24372000**	**0.52650900**
**C**	**2.27209300**	**-0.86287800**	**-0.99124300**
**H**	**2.21954100**	**-1.73996100**	**-0.34385100**
**O**	**0.91078900**	**-0.57817400**	**-1.45632200**
**H**	**0.64633100**	**-1.28614600**	**-2.08273400**
**C**	**2.75376000**	**0.31722300**	**-0.20782100**
**C**	**3.01992900**	**0.26415100**	**1.09958500**
**H**	**2.94006900**	**1.23177600**	**-0.77314500**
**H**	**2.79506500**	**-0.65957400**	**1.63283600**
**C**	**3.13787300**	**-1.14221200**	**-2.21553300**
**H**	**4.16008500**	**-1.37234300**	**-1.90198800**
**H**	**2.75406300**	**-2.00198800**	**-2.77504700**
**H**	**3.16556700**	**-0.27360200**	**-2.88157300**
**C**	**3.62494100**	**1.37600500**	**1.90700200**
**C**	**2.77997200**	**1.78169300**	**3.12421400**
**H**	**3.80740800**	**2.25063800**	**1.26999600**
**H**	**4.61027500**	**1.04676100**	**2.26993900**
**C**	**3.48203800**	**2.78211400**	**4.04663100**
**H**	**1.83478700**	**2.21906400**	**2.77753100**
**H**	**2.50972100**	**0.88327100**	**3.69497400**
**C**	**2.61634400**	**3.19476100**	**5.23902100**

H	4.42142800	2.34168600	4.40606600
H	3.76446800	3.67230100	3.46873000
H	3.13739300	3.90588300	5.88759500
H	1.68563900	3.66780600	4.90536600
H	2.34430700	2.32483500	5.84715700

1b-TS1

Ir	-0.62562700	0.16548000	0.01428000
Cl	0.98914800	-0.17698700	1.68741600
Cl	-0.24034300	-2.78172000	-1.83184100
C	-2.06227500	1.55728000	-0.78979600
C	-2.48146800	0.24129000	-1.17687200
C	-2.67657500	-0.52764000	0.03794800
C	-2.43508300	0.33344800	1.18499600
C	-2.04116500	1.62052000	0.67597900
C	-1.71637400	2.68165400	-1.70875900
H	-1.32400200	2.30363200	-2.65397200
H	-0.95798500	3.33082300	-1.26675100
C	-2.65992000	-0.28588000	-2.56224700
H	-3.72707500	-0.41275900	-2.77848800
H	-2.24149100	0.39883200	-3.30177900
H	-2.15543400	-1.25222900	-2.66567500
C	-3.09250300	-1.95744900	0.07901700
H	-2.56359500	-2.52752500	-0.68822300
H	-2.87443300	-2.40677100	1.04861300
H	-4.17440400	-2.01925700	-0.09612600
C	-2.58353800	-0.04382300	2.62297800
H	-3.59437700	0.18756400	2.97823500
H	-2.40444600	-1.11054600	2.76564100
H	-1.86417000	0.49347500	3.24318600
C	-1.72579100	2.82911700	1.49343300
H	-1.30825300	2.54971300	2.46185400
H	-1.00735500	3.47763600	0.99060100
H	-2.64276700	3.40569800	1.66661800
H	-2.60560500	3.28747200	-1.92005300
C	2.19662200	0.05188300	-1.46893900
H	2.53124300	-0.38800600	-0.52636800
O	0.75053800	-0.14467900	-1.57858200
H	0.51463900	-1.13733100	-1.80009000
C	2.42308300	1.53176200	-1.45959500
C	2.79372300	2.22050500	-0.37829800
H	2.24080000	2.04741600	-2.40388000
H	2.93941300	1.68166600	0.55688500
C	2.84702000	-0.65584600	-2.65139200
H	3.93412300	-0.54884000	-2.59069800
H	2.59441000	-1.71946500	-2.64688300
H	2.50590600	-0.22052100	-3.59609200
C	2.94845200	3.71192200	-0.32230100
C	1.79293000	4.36796800	0.45391900
H	2.98946500	4.12856000	-1.33630500
H	3.89707000	3.97177400	0.16727000
C	1.94480200	5.88038600	0.63008800
H	0.85752100	4.15463000	-0.08126000
H	1.69591900	3.88867300	1.43726000
C	0.75802700	6.50980500	1.36341100
H	2.87188000	6.08869400	1.17982700
H	2.06239500	6.35049900	-0.35528300
H	0.88402700	7.59030700	1.48345200
H	-0.17670300	6.34373500	0.81474700
H	0.63684400	6.07503600	2.36208700

1b-Int2

Ir	-0.67566500	0.67206700	0.91548000
Cl	0.24907500	-1.16281900	2.18643800
C	-2.17679100	1.79362500	-0.29114000
C	-2.76208100	1.40091100	0.98166700
C	-2.75224700	-0.03681200	1.05383100
C	-2.14456900	-0.54460300	-0.16628600
C	-1.83163000	0.59199100	-1.00313000
C	-2.10450900	3.18887200	-0.82189700
H	-1.28682600	3.30775600	-1.53494200
H	-3.03708100	3.43981000	-1.33997500
C	-3.42069700	2.32329900	1.95171400
H	-4.47952000	2.42075700	1.68411400
H	-2.98223200	3.32157400	1.92346000
H	-3.36679700	1.94891500	2.97344300

C	-3.31365000	-0.88173200	2.14733300
H	-3.46705700	-0.30893400	3.06223600
H	-2.65157800	-1.71926600	2.37345000
H	-4.28332700	-1.28251600	1.82988000
C	-1.99242900	-1.98649900	-0.52446700
H	-2.93561600	-2.37780700	-0.92210300
H	-1.71478600	-2.57557500	0.35108000
H	-1.22101300	-2.12855600	-1.28334600
C	-1.20040400	0.51965300	-2.35181500
H	-0.47600700	-0.29494800	-2.40716600
H	-0.69369000	1.45029000	-2.60984600
H	-1.97311200	0.33425000	-3.10702500
H	-1.96807000	3.91774500	-0.02079600
C	1.82384000	1.92229900	0.47968400
H	2.73877400	1.78314000	1.06294900
C	0.68222500	2.44140800	1.34650200
C	0.47733200	1.92837200	2.61751800
H	0.19487200	3.36260700	1.03764000
H	1.17089000	1.17035800	2.97466200
O	1.36446200	0.58474600	0.03783000
H	1.77896300	-0.12461800	0.56777100
C	2.08864500	2.75569100	-0.75340000
H	2.42295200	3.75536300	-0.46003900
H	2.86417800	2.29663200	-1.36963600
H	1.17834700	2.85684600	-1.35181200
C	-0.40066000	2.52982800	3.67211300
C	-1.07793800	1.48984700	4.57283500
H	-1.13441000	3.20571400	3.22185800
H	0.25200800	3.16206400	4.29340300
C	-1.97636900	2.11552500	5.64215300
H	-1.65476400	0.79901900	3.94791000
H	-0.30789700	0.87152900	5.04995900
C	-2.67278800	1.06459900	6.50890100
H	-1.37740600	2.78151800	6.27603400
H	-2.72856900	2.75633200	5.16082300
H	-3.30761000	1.53102500	7.26705900
H	-3.30643900	0.40633800	5.90303700
H	-1.94254100	0.43344000	7.02568800

1b-TS2

Ir	-0.01809900	0.52150600	-0.16358400
Cl	0.65289400	2.64227100	-1.11733200
C	-1.61547400	1.44771200	1.06273600
C	-1.18273900	0.27062800	1.76187600
C	-1.42123400	-0.87179800	0.91153200
C	-2.10352600	-0.40944600	-0.28231000
C	-2.18978800	1.01917300	-0.21509000
C	-1.61819500	2.85224300	1.56905200
H	-1.36134600	3.55086800	0.77106300
H	-2.61299500	3.10884100	1.95035300
C	-0.56504200	0.21477400	3.12104900
H	-0.15406500	1.18206400	3.41292600
H	0.23149000	-0.53139400	3.16215400
H	-1.32419100	-0.06584400	3.85984800
C	-1.16194200	-2.29661500	1.28251200
H	-0.25102600	-2.39082600	1.87689700
H	-1.06021600	-2.92950300	0.39868200
H	-1.99512400	-2.68949900	1.87619900
C	-2.69787500	-1.28672600	-1.33245100
H	-3.71765200	-1.55656600	-1.03249600
H	-2.13816600	-2.21562300	-1.45312300
H	-2.75593500	-0.78785700	-2.29913000
C	-2.86089700	1.93196000	-1.18623300
H	-3.03810500	1.44245500	-2.14434300
H	-2.25404000	2.82222800	-1.36280000
H	-3.82906000	2.24889000	-0.78173800
H	-0.89830900	2.98739300	2.37767600
C	2.28835100	-0.32344700	0.15800800
H	1.39319200	0.78934400	0.68108000
C	1.43718700	-0.96823000	-0.84223700
C	1.06253200	-0.31452900	-2.02662400
H	1.12361100	-1.98509800	-0.62431200
H	1.61789200	0.56499100	-2.33482400
O	2.47234100	-1.11515300	1.24579800
H	3.12956100	-0.72228600	1.83882800
C	0.26964400	-0.97628700	-3.11400700

C	-0.56685300	0.01866100	-3.92652300
H	-0.35977000	-1.77008600	-2.69907100
H	0.98501600	-1.47300900	-3.78607400
C	-1.40112100	-0.65166000	-5.02083100
H	-1.21648800	0.57891800	-3.24447400
H	0.09839500	0.76758300	-4.37357400
C	-2.27523800	0.34473800	-5.78499000
H	-0.73260700	-1.17115600	-5.71880900
H	-2.03318700	-1.43171900	-4.57374000
H	-2.85346200	-0.15226800	-6.56861600
H	-2.98403700	0.84564300	-5.11528500
H	-1.66573900	1.12070500	-6.25928000
C	3.42649400	0.59424700	-0.22591800
H	4.22856400	-0.01409800	-0.65696300
H	3.10554700	1.34580600	-0.94624300
H	3.80992000	1.11995100	0.65348400

1b-Int3

Ir	0.04504400	0.57936800	-0.12152300
Cl	0.61343200	2.65318300	-1.24213000
C	-1.59172800	1.44176700	1.04678800
C	-1.18133400	0.22838400	1.71411800
C	-1.45183100	-0.86852300	0.81725800
C	-2.16367000	-0.35647300	-0.33889100
C	-2.21666500	1.05883500	-0.22277900
C	-1.60304200	2.82639400	1.60988100
H	-1.34138400	3.55706100	0.84235500
H	-2.60081600	3.06768600	1.99317700
C	-0.64806700	0.11022700	3.10699700
H	-0.16647200	1.03459900	3.42880700
H	0.07468400	-0.70341100	3.19448200
H	-1.47189200	-0.09855800	3.79900000
C	-1.22810000	-2.31473900	1.12343500
H	-0.36881700	-2.45653700	1.78136300
H	-1.06848500	-2.89996100	0.21507400
H	-2.10764300	-2.72918700	1.62973000
C	-2.82440200	-1.19192200	-1.38441500
H	-3.84962700	-1.41685500	-1.06575200
H	-2.31672100	-2.14698200	-1.52753100
H	-2.88088500	-0.68206300	-2.34543800
C	-2.86170500	2.02044700	-1.16278100
H	-3.11525200	1.54892500	-2.11292000
H	-2.19858700	2.86518900	-1.36341200
H	-3.78455900	2.40873500	-0.71685400
H	-0.88960100	2.92911100	2.42896800
C	2.53108600	-0.41085200	0.07526900
H	1.10571200	1.20932300	0.86556100
C	1.51319100	-0.95977100	-0.74944000
C	1.04030600	-0.30622600	-1.92067600
H	1.16583900	-1.95306200	-0.47979500
H	1.62649500	0.52114300	-2.30635200
O	2.80854700	-1.12099900	1.15937300
H	3.51326700	-0.70664500	1.68155700
C	0.22443200	-1.01003800	-2.96709700
C	-0.57151500	-0.03782900	-3.84399700
H	-0.43249800	-1.75555300	-2.50919300
H	0.92480900	-1.57137500	-3.60388600
C	-1.38503400	-0.73833800	-4.93503500
H	-1.22793900	0.56493200	-3.20615400
H	0.12085100	0.67818600	-4.30360200
C	-2.22091500	0.24125400	-5.76128500
H	-0.70455100	-1.29281900	-5.59348200
H	-2.04159300	-1.49184100	-4.47859900
H	-2.78300700	-0.27563800	-6.54380400
H	-2.94131500	0.77659700	-5.13189000
H	-1.58560500	0.99075400	-6.24434100
C	3.45283000	0.70667300	-0.29392500
H	4.31456700	0.27643700	-0.82029800
H	2.96984600	1.44400900	-0.93372000
H	3.81540900	1.22184800	0.60053700

1b-Int4

Ir	1.48086500	0.23943100	0.64197200
Cl	3.21325000	1.94141100	0.73610400
C	-0.17020300	1.67922400	1.00666100
C	-0.64021200	0.35761200	1.31385600

C	-0.61542400	-0.39668800	0.08341800
C	-0.27662100	0.50150300	-1.01262400
C	0.01565700	1.75968500	-0.44779000
C	-0.06909600	2.84969000	1.93350000
H	0.86387600	3.39174100	1.76073400
H	-0.90745400	3.54086600	1.78451100
C	-1.10133200	-0.16973400	2.63779100
H	-0.72604100	0.44529100	3.45764600
H	-0.74782700	-1.19131700	2.79442000
H	-2.19693700	-0.17245500	2.68809500
C	-1.10766000	-1.80397900	-0.05630800
H	-0.75736800	-2.43056600	0.76756000
H	-0.77084100	-2.25485200	-0.99277000
H	-2.20426100	-1.81673000	-0.06059600
C	-0.28328100	0.13942800	-2.46461200
H	0.39386800	0.77261900	-3.04054400
H	-1.28930000	0.24948000	-2.88828500
H	0.02163700	-0.89795400	-2.62068300
C	0.44953000	2.99914600	-1.15756100
H	0.60287400	2.82106400	-2.22341200
H	1.38806000	3.36665400	-0.73045400
H	-0.30635100	3.78514700	-1.04729000
H	-0.07687400	2.52625100	2.97622900
C	2.30011000	-2.40742800	1.82084500
H	1.99871600	0.03644800	2.10403700
C	2.13386500	-1.82644900	0.45623600
C	3.04826700	-1.01271800	-0.25882000
H	1.39138800	-2.37155100	-0.11968600
O	1.39919400	-3.09104000	2.29218900
C	3.59692500	-2.17182700	2.56819600
H	4.38449500	-2.78713100	2.11690800
H	3.91799500	-1.12904300	2.50940900
H	3.47089400	-2.46778300	3.61029200
H	3.97239600	-0.71576200	0.22953700
C	3.10326800	-1.03843100	-1.76879900
H	3.89772500	-1.73986400	-2.06743700
H	2.16852700	-1.45963500	-2.15939500
C	3.36832800	0.32186100	-2.41862300
H	4.30233400	0.74022200	-2.02527200
H	2.58618400	1.01985400	-2.10152900
C	3.42428100	0.25831700	-3.94641700
H	2.49670500	-0.19254000	-4.32683300
H	4.23527300	-0.41327200	-4.25857600
C	3.62229600	1.63576100	-4.58327900
H	3.66099500	1.57583300	-5.67565100
H	2.80415600	2.31389900	-4.31364400
H	4.55395700	2.09706300	-4.23838900

1b-TS3

Ir	1.01739600	0.36096700	-0.04690500
Cl	1.63265600	2.26342700	-1.47078000
C	-0.57134000	1.63259300	0.81513400
C	-0.22731800	0.62008200	1.77043600
C	-0.57953200	-0.65210300	1.19949800
C	-1.30981300	-0.39354700	-0.05776100
C	-1.30148000	0.98474500	-0.29020900
C	-0.48616700	3.11261800	1.00848200
H	-0.17657000	3.60254500	0.08368500
H	-1.46264300	3.51092400	1.31092100
C	0.32777400	0.84261300	3.14298900
H	0.93013400	1.75137900	3.18462900
H	0.95536900	0.00695900	3.45785600
H	-0.49146100	0.94258800	3.86558800
C	-0.63360200	-1.94162700	1.95899900
H	0.18682300	-2.01881800	2.67548800
H	-0.59957500	-2.80863000	1.29969900
H	-1.57255800	-1.99069000	2.52567700
C	-1.94514200	-1.45964900	-0.88980400
H	-2.25235000	-1.07485000	-1.86413600
H	-2.83888200	-1.84591300	-0.38362500
H	-1.26701100	-2.30408800	-1.04290300
C	-1.86518300	1.73690300	-1.44940600
H	-2.26178000	1.06442200	-2.21221600
H	-1.08663000	2.35784000	-1.90438000
H	-2.67607500	2.39749100	-1.12014100
H	0.24149400	3.36955900	1.78049400

C	1.43396600	-2.69861000	-0.32817300
H	2.30207700	0.65757900	0.80778100
C	1.62875700	-1.48475500	-1.16191100
C	2.79100300	-0.70829000	-1.09338700
H	1.06184100	-1.49683300	-2.09059400
O	0.56244800	-3.51172500	-0.61948800
C	2.36675900	-2.95097900	0.84248100
H	3.34349200	-3.27694700	0.46741800
H	2.51823600	-2.04737400	1.43738000
H	1.94989400	-3.74682400	1.45975300
H	3.49084500	-0.93033700	-0.29470900
C	3.41814900	-0.08500400	-2.30710500
H	2.65098600	0.17188400	-3.04157900
H	3.91768400	0.84899500	-2.03894300
C	4.43586200	-1.06418000	-2.92267700
H	3.93030900	-2.00353900	-3.18507300
H	5.19929400	-1.32455400	-2.17593400
C	5.12016000	-0.48713500	-4.16601500
H	5.61504600	0.45558600	-3.89932100
H	4.35457700	-0.22909700	-4.90906800
C	6.13708500	-1.44896700	-4.78447000
H	6.60801900	-1.01669400	-5.67305200
H	6.93237600	-1.69455900	-4.07161400
H	5.65998700	-2.38910600	-5.08354000

1b-Int5

Ir	-0.07280900	0.73308000	-0.48396700
Cl	-0.24131600	2.43162800	-2.21417000
C	-1.76729600	1.42843300	0.73832000
C	-0.99442200	0.47061200	1.49175400
C	-1.11794300	-0.79799400	0.80675200
C	-2.09405100	-0.66034500	-0.26204600
C	-2.46835200	0.69971700	-0.31712200
C	-2.01888100	2.86444400	1.07976500
H	-2.00987600	3.47783600	0.17559400
H	-2.99125900	2.98708900	1.57258100
C	-0.30953800	0.70607400	2.80145600
H	0.02285700	1.74185100	2.89315600
H	0.56498000	0.06338900	2.91540800
H	-0.99605900	0.49545000	3.63055000
C	-0.54947000	-2.10924700	1.24754200
H	0.29912700	-1.97252100	1.92076500
H	-0.22092000	-2.69208300	0.38324600
H	-1.31337300	-2.68731600	1.78168200
C	-2.60899100	-1.78905700	-1.09203000
H	-3.24154900	-2.44695800	-0.48324400
H	-1.78953000	-2.39477600	-1.49080600
H	-3.20911200	-1.42747900	-1.92930700
C	-3.40571600	1.34774700	-1.28082700
H	-3.82696200	0.62553500	-1.98189800
H	-2.87711800	2.11621800	-1.85491400
H	-4.23022600	1.82954000	-0.74392600
H	-1.24815500	3.24971400	1.75027400
C	0.48340400	-1.36241800	-2.53205600
H	0.98429700	1.74990600	0.05181800
C	1.26678300	-0.29180000	-1.87307100
C	1.78818100	-0.44823300	-0.56454100
H	1.68878400	0.46604900	-2.52676900
H	1.58184700	-1.40494700	-0.09003100
O	0.28128000	-2.45650000	-2.00525100
C	3.07550800	0.20312300	-0.12121000
C	-0.06605200	-1.02634800	-3.90125200
H	-0.50945000	-0.02560800	-3.89501500
H	0.75319100	-1.01090200	-4.63030900
H	-0.79553800	-1.77797300	-4.20656200
H	3.89737000	-0.45841500	-0.43707400
H	3.21378300	1.15247200	-0.65040100
C	3.17867600	0.42361900	1.38940600
H	2.98108300	-0.52575500	1.90880900
H	2.38801400	1.11605100	1.70135400
C	4.53775000	0.97079900	1.83191900
H	4.73651400	1.91075700	1.30011700
H	5.32940400	0.27402000	1.52593500
C	4.61706300	1.20939200	3.34139800
H	3.85424600	1.92598100	3.66662000
H	4.45415000	0.27888200	3.89718700

H	5.59371200	1.60629100	3.63571300

1b-TS4

Ir	-0.22942600	0.49908300	-0.15137000
Cl	0.54921700	2.65821000	-0.98952500
C	-2.01434700	1.34303700	0.88257900
C	-1.54771700	0.23720500	1.65155200
C	-1.60732400	-0.95200100	0.81587300
C	-2.22491900	-0.57536800	-0.43865400
C	-2.41296200	0.84125700	-0.43434800
C	-2.14016800	2.76809500	1.31577100
H	-1.71919000	3.43387600	0.55868100
H	-3.19373500	3.03094900	1.46884100
C	-1.07992900	0.27894500	3.07312900
H	-0.67770900	1.26093400	3.32822500
H	-0.29818000	-0.46128000	3.25471100
H	-1.91148300	0.06313600	3.75478300
C	-1.28761800	-2.35216400	1.23725800
H	-0.59310300	-2.35693100	2.08065200
H	-0.82195200	-2.90340100	0.41780900
H	-2.19572500	-2.88257000	1.54957000
C	-2.63196600	-1.53258300	-1.50982200
H	-3.62448400	-1.93742000	-1.27641600
H	-1.93636900	-2.37076000	-1.57996100
H	-2.69220200	-1.04620500	-2.48528000
C	-3.01386600	1.68352300	-1.51402900
H	-2.99247000	1.16566300	-2.47513100
H	-2.45364100	2.61480600	-1.62527200
H	-4.05743800	1.93104300	-1.28390900
H	-1.60491700	2.94779400	2.24994000
C	0.55975400	-1.59778600	-2.17501800
H	1.15189100	0.46132600	0.72094700
C	1.12525200	-0.36397100	-1.59639700
C	1.84646500	-0.40216700	-0.34205700
H	1.41798800	0.39968100	-2.31190000
H	1.83438300	-1.37113100	0.15521100
O	0.45374000	-2.65427300	-1.55502300
C	3.13743800	0.38558400	-0.20218500
C	0.10682500	-1.48863700	-3.61932700
H	-0.46088000	-0.56697200	-3.78013100
H	0.98685400	-1.44125200	-4.27179400
H	-0.48946800	-2.35970400	-3.89380100
H	3.90820000	-0.19021400	-0.73449300
H	3.02650300	1.34167400	-0.72126600
C	3.57935600	0.62487300	1.24173600
H	3.63325200	-0.33325500	1.77862800
H	2.81195300	1.22495900	1.75076600
C	4.92663200	1.34496100	1.34195200
H	4.86750500	2.29005600	0.78688100
H	5.69741600	0.74339500	0.84181600
C	5.34789900	1.62277100	2.78640200
H	4.60966100	2.25173100	3.29644500
H	5.44068400	0.69202600	3.35762600
H	6.31236200	2.13847900	2.83064800

1b-Int6

Ir	-1.07908400	0.98272100	-0.57589700
Cl	-1.00613500	2.84063300	-2.00645900
C	-2.97659200	1.15594000	0.67278800
C	-1.85892300	0.49855500	1.37373200
C	-1.56048600	-0.74144700	0.71248800
C	-2.34087800	-0.74426100	-0.50183100
C	-3.28129400	0.38674700	-0.46511800
C	-3.60553900	2.44524200	1.09035000
H	-4.07908200	2.94623700	0.24454900
H	-4.36755100	2.27396400	1.86024900
C	-1.26157900	0.97219300	2.65796400
H	-1.21943500	2.06304500	2.69315800
H	-0.24670700	0.59023100	2.78170100
H	-1.86548600	0.62959200	3.50749000
C	-0.67824000	-1.85244300	1.18285700
H	-0.13870100	-1.57034400	2.08883200
H	0.05152900	-2.12290000	0.41453200
H	-1.28958200	-2.73162400	1.41882200
C	-2.31896600	-1.83220600	-1.52462100
H	-2.99329700	-2.64411300	-1.22378800

H	-1.31033100	-2.23714400	-1.62886300
H	-2.64808800	-1.46501300	-2.49896900
C	-4.29332300	0.69018300	-1.52088400
H	-3.90962900	0.44032400	-2.51270700
H	-4.55648600	1.74916600	-1.52583100
H	-5.20828700	0.10797600	-1.35756700
H	-2.85969100	3.12833800	1.50337500
C	1.00957700	-0.47852300	-1.82234900
H	1.59055100	1.35136200	1.06253200
C	1.00081400	0.70233600	-0.90827700
C	1.79663900	0.51483600	0.38290300
H	1.33589300	1.59366400	-1.44776100
H	1.47289300	-0.39847200	0.88714100
O	1.11781100	-1.63914300	-1.41878500
C	3.31014700	0.42999700	0.13839300
C	0.88139400	-0.20150200	-3.31023600
H	0.27002000	0.68189700	-3.50789100
H	1.88409300	-0.00628300	-3.71068300
H	0.47667700	-1.07782000	-3.82090300
H	3.51445300	-0.42103900	-0.52305200
H	3.64358000	1.33073600	-0.39522500
C	4.11580700	0.27570100	1.43084100
H	3.77444700	-0.62203900	1.96603800
H	3.90395200	1.12513400	2.09635000
C	5.62688300	0.18043800	1.19977800
H	5.96529900	1.07387000	0.65806200
H	5.83662300	-0.67200000	0.54015300
C	6.42453500	0.03489300	2.49790000
H	6.25625400	0.89073600	3.16159100
H	6.12787300	-0.86800100	3.04387500
H	7.50029300	-0.03084700	2.30527200

1b-Int7

Ir	-1.01697300	-0.79505400	-0.14082200
Cl	-0.48001700	-3.00283700	-0.62519500
C	-1.87608100	1.12693100	0.61261300
C	-1.47754400	0.25882400	1.69368600
C	-2.30782200	-0.94924300	1.66325300
C	-3.12496400	-0.87304000	0.50161800
C	-2.83989000	0.40571400	-0.16897900
C	-1.42083900	2.53544000	0.41373900
H	-1.70529600	2.91335700	-0.56844200
H	-1.89039900	3.17828300	1.16677800
C	-0.48881100	0.59956600	2.75693700
H	0.25676700	1.30330400	2.38405200
H	0.01839300	-0.29159200	3.13205700
H	-1.00203500	1.07237700	3.60288600
C	-2.26013600	-2.06515800	2.65086500
H	-1.26185400	-2.17853700	3.07716300
H	-2.54079200	-3.01365600	2.19148000
H	-2.95706400	-1.86158400	3.47258200
C	-4.12233500	-1.88315700	0.04120600
H	-5.11280100	-1.63331900	0.43922000
H	-3.85903300	-2.88556000	0.38066000
H	-4.19333200	-1.90362000	-1.04785700
C	-3.53297900	0.88620100	-1.39746500
H	-3.67713200	0.07350700	-2.11278200
H	-2.97657900	1.68474100	-1.88829500
H	-4.52356200	1.27432100	-1.13073300
H	-0.33832800	2.62684000	0.51330700
C	1.42629400	0.04326000	-0.55879800
H	-0.88984600	0.76966200	-2.93858000
C	0.53992300	-0.13050700	-1.62687200
C	0.07097400	1.02831500	-2.48193700
H	0.68663600	-1.07260700	-2.15296600
H	-0.08913900	1.91715300	-1.86848900
O	1.60553600	1.28399700	-0.07802400
C	1.08218100	1.35822500	-3.59546300
C	2.32783400	-1.02233200	-0.01576400
H	2.03422400	-2.00937000	-0.36838000
H	3.35112100	-0.81165900	-0.35322900
H	2.32589200	-1.02496800	1.07967600
H	2.04741200	1.61580400	-3.14053600
H	1.25398400	0.46507300	-4.20944800
C	0.60433600	2.51054000	-4.48295500
H	0.43377800	3.40070000	-3.86111600

H	-0.36980500	2.25083600	-4.92045500
C	1.58752700	2.85664900	-5.60622800
H	1.75447600	1.96586900	-6.22553700
H	2.56122000	3.11129600	-5.16748300
C	1.09982400	4.01106600	-6.48412700
H	0.14327100	3.76884700	-6.95994500
H	0.95601400	4.92294700	-5.89421600
H	1.81805800	4.23736700	-7.27721000
H	2.24413300	1.27533000	0.65032300

enol-2b

C	4.81562900	-1.41463800	-0.49022600
H	2.92084300	-0.08522900	-2.86037100
C	3.94789900	-1.39045400	-1.51313300
C	3.95120200	-0.39796600	-2.64394400
H	3.20352000	-2.18001400	-1.55011600
H	4.47039500	0.53122300	-2.35947400
O	5.82746000	-0.50654700	-0.30530700
C	4.58800200	-0.94139900	-3.93632800
C	4.81116700	-2.42913000	0.61122400
H	4.00430900	-3.15146300	0.47538100
H	5.76654500	-2.96518100	0.64107500
H	4.68805400	-1.93765700	1.58295900
H	5.62511700	-1.23372800	-3.72535100
H	4.06877200	-1.86379800	-4.22804900
C	4.55397100	0.05711000	-5.09571400
H	5.06942700	0.98164300	-4.79753000
H	3.51252900	0.34484400	-5.29848000
C	5.18945500	-0.48192900	-6.38118900
H	4.67308000	-1.40446100	-6.67812300
H	6.22940800	-0.76915100	-6.17599500
C	5.15204800	0.52258200	-7.53519100
H	4.12128400	0.79952300	-7.78365400
H	5.68614400	1.44318600	-7.27385200
H	5.61466300	0.11426100	-8.43937900
H	5.81101200	0.13023500	-1.03215000

1b-TS6

C	-4.76605800	8.66123000	0.32226900
C	-5.42257100	9.62083800	-0.47533800
O	-4.08417400	8.93726500	1.37211600
H	-4.63612100	9.88103700	2.15814500
O	-5.29259100	10.48672700	2.79667100
H	-6.34432400	10.43178900	2.18500800
O	-7.22069600	10.15335900	1.40966800
H	-6.57177300	9.84897400	0.52083800
C	-8.19970800	11.16855800	1.08034600
H	-8.35006000	11.11758700	-0.00538600
C	-4.76568700	11.78834900	3.12584400
H	-5.11907000	12.51439800	2.38365100
C	-7.68489300	12.53261400	1.44294000
C	-7.33160300	13.47008300	0.56172800
H	-7.57609100	12.72936700	2.51036200
H	-7.45002300	13.26000200	-0.50239400
C	-3.26549000	11.74128300	3.08992300
C	-2.50492500	12.64491000	2.47050600
H	-2.80664500	10.91474000	3.63263600
H	-2.99104500	13.45779400	1.92901600
C	-4.85803800	11.02875100	-0.58618600
H	-4.58921600	11.40791800	0.40341300
H	-5.62548900	11.71154100	-0.97168600
H	-5.85029800	9.22890000	-1.39893400
C	-4.91472100	7.18693500	0.00850700
H	-5.21750500	6.64425000	0.90892400
H	-5.62622500	6.99712700	-0.79804100
H	-3.93585300	6.79369300	-0.28916000
C	-3.61533600	11.11110100	-1.48522500
C	-3.09926500	12.53867000	-1.67623600
H	-3.84420400	10.66889400	-2.46466000
H	-2.82706500	10.48815800	-1.04260100
C	-1.83555400	12.62423600	-2.53571300
H	-3.88852300	13.15605300	-2.12890200
H	-2.89724800	12.98296800	-0.69348200
C	-1.32734500	14.05631500	-2.71586300
H	-1.04809300	12.01159400	-2.07904100
H	-2.03574000	12.17790600	-3.51896500

H	-0.42256100	14.08987600	-3.33170800
H	-2.08335400	14.68601200	-3.19928600
H	-1.08984100	14.51519400	-1.74974600
C	-9.49722400	10.81864700	1.80099900
H	-10.28980700	11.52369200	1.53147500
H	-9.81542900	9.80646000	1.53839000
H	-9.35171700	10.86098900	2.88544200
C	-6.69443700	14.78383600	0.90902300
C	-5.28212300	14.89259400	0.30710600
H	-6.64132900	14.89100600	1.99976500
H	-7.30664200	15.61822100	0.53848800
C	-4.52988900	16.15652400	0.72905400
H	-4.70415800	14.00837900	0.59886000
H	-5.34943200	14.85179800	-0.78796100
C	-3.12422300	16.23332300	0.12714700
H	-5.10855600	17.04198300	0.43414900
H	-4.46324400	16.18732900	1.82501000
H	-2.60568900	17.14794400	0.43125700
H	-2.51203000	15.38193200	0.44491400
H	-3.16141100	16.21505800	-0.96718500
C	-5.30500800	12.16836900	4.50330200
H	-4.97847300	13.17645800	4.77493200
H	-6.39960300	12.13999400	4.51019500
H	-4.94311500	11.46483600	5.25866600
C	-1.00517800	12.63319800	2.42914400
C	-0.45909100	12.52919500	0.99541200
H	-0.62479400	11.79980400	3.03199000
H	-0.61671000	13.55549300	2.88621600
C	1.07002000	12.53765900	0.92868000
H	-0.84699800	11.61357900	0.53182000
H	-0.85125000	13.36285700	0.39820700
C	1.60183600	12.43686700	-0.50306900
H	1.44708800	13.45587600	1.39899600
H	1.46250900	11.70524900	1.52770200
H	2.69624800	12.44553900	-0.52719200
H	1.26498700	11.51114900	-0.98270600
H	1.24650900	13.27274900	-1.11552200

2b

C	0.81709500	-0.16323700	-0.05922300
C	1.81892200	0.17603300	-1.15344300
C	2.35821500	-1.04906600	-1.91031400
H	1.32639000	0.87019200	-1.84046400
H	1.51595700	-1.62151600	-2.32081500
O	-0.29284700	0.33472600	-0.03678000
C	1.25896400	-1.15660200	1.00254200
H	2.27365600	-0.94212200	1.35435900
H	0.55660600	-1.13540000	1.83667800
H	1.27387100	-2.16775000	0.57923000
C	3.31239400	-0.66534200	-3.04512900
H	2.79348200	0.00541800	-3.74326300
H	4.15065400	-0.08612700	-2.63318500
C	3.85682100	-1.87458500	-3.81006700
H	4.37437000	-2.54597000	-3.11021400
H	3.01741700	-2.45326800	-4.22044700
C	4.81258300	-1.49493000	-4.94546200
H	5.64983800	-0.91579600	-4.53349800
H	4.29437800	-0.82457100	-5.64404400
C	5.35242000	-2.70948100	-5.70400500
H	6.03211200	-2.41131100	-6.50850600
H	5.90225100	-3.38030300	-5.03417200
H	4.53720900	-3.28801100	-6.15292900
H	2.87802000	-1.71911500	-1.21417500
H	2.66233800	0.70854300	-0.69023500

1b-TS6-Cl

C	-4.25084900	8.66140800	1.51539900
C	-5.52857900	8.55063100	0.89660900
C	-5.77343900	7.50652600	-0.17007200
H	-6.35290400	8.69410800	1.59142400
H	-6.65893800	7.79048600	-0.74777100
H	-4.93682200	7.48658600	-0.86853100
O	-3.13304800	8.44656000	0.90622900
H	-3.13095600	8.48859300	-0.15217300
C	-6.07584400	13.13766700	-1.53299600
C	-5.53978000	14.06464800	-2.64110600

C	-4.26387200	13.37306100	-3.13635500
C	-4.17201900	12.10788100	-2.27815700
N	-5.20573700	12.06135900	-1.41153000
H	-6.30864700	14.17272500	-3.41100400
H	-3.35453500	13.96379700	-2.99251200
Cl	-5.39151300	10.39536200	-0.11266400
O	-3.25898000	11.27566200	-2.39471600
O	-7.08305600	13.31905900	-0.88166600
H	-4.29590700	13.08396500	-4.19096400
H	-5.36557400	15.05683200	-2.21602200
H	-3.00520700	9.81142600	-1.70778400
O	-2.80281900	8.84871800	-1.50739300
C	-3.17425900	8.08384500	-2.66367500
H	-4.24154700	8.25802800	-2.87085100
C	-2.97454800	6.61884400	-2.39009500
C	-2.21183000	6.10331100	-1.42391100
H	-3.48941900	5.95228500	-3.08187300
H	-1.68763700	6.77777700	-0.74865700
C	-4.11422700	9.08609900	2.94345100
H	-3.95863200	8.19326600	3.56242300
H	-3.24307400	9.73316600	3.06426000
H	-5.01524100	9.59137300	3.29364700
C	-5.95694500	6.10308200	0.42815900
C	-6.06650800	5.01720200	-0.64512900
H	-6.84507600	6.08603600	1.07395600
H	-5.10081600	5.87738600	1.07792900
C	-6.22725300	3.60717100	-0.06896800
H	-6.91158100	5.24028600	-1.31083000
H	-5.16620700	5.05335500	-1.27176100
C	-6.18371300	2.51780300	-1.14223100
H	-5.43096600	3.42448500	0.66419000
H	-7.17181300	3.54549900	0.48723900
H	-6.30411800	1.52026900	-0.70816500
H	-6.97846000	2.65809800	-1.88348300
H	-5.22728300	2.53555500	-1.67755300
C	-2.35926400	8.55210100	-3.87330700
H	-2.64355800	7.98682200	-4.76663800
H	-2.53227100	9.61548200	-4.05439400
H	-1.29508900	8.38937200	-3.68170200
C	-2.03579700	4.63457800	-1.16629000
C	-2.39746900	4.24482600	0.27585300
H	-0.99089200	4.34683000	-1.35422700
H	-2.64904800	4.05814100	-1.87072700
C	-2.31184900	2.73894900	0.53573700
H	-1.73610000	4.77875200	0.97173200
H	-3.41294700	4.59278500	0.49514200
C	-2.66089200	2.36455900	1.97792500
H	-2.98942000	2.21729100	-0.15339500
H	-1.30042300	2.38477600	0.29595800
H	-2.59808300	1.28385500	2.13933000
H	-1.97992300	2.84885600	2.68678300
H	-3.67933700	2.68005000	2.23175100

3b

C	-4.20697400	8.44959100	1.62551500
C	-5.57118700	8.55694300	0.92013500
C	-5.74513700	7.55889500	-0.21061600
H	-6.37470800	8.47168000	1.65823800
H	-4.95745200	7.72508400	-0.95170100
H	-5.55018400	6.56740700	0.21848400
O	-3.32626200	7.76241600	1.15138800
Cl	-5.70758200	10.28621300	0.30796800
C	-4.05001800	9.19776500	2.93038400
H	-4.70264800	8.74484800	3.68694400
H	-3.01436200	9.13516900	3.26517400
H	-4.35364300	10.24231200	2.82425700
C	-7.12732200	7.57940800	-0.86503800
C	-7.27039300	6.51981500	-1.96090400
H	-7.31701200	8.57210400	-1.28891900
H	-7.89885400	7.41889100	-0.09856100
C	-8.64533100	6.53086300	-2.63582500
H	-6.49215100	6.67709300	-2.72057100
H	-7.08194100	5.52386000	-1.53588800
C	-8.78088800	5.47197300	-3.73221500
H	-9.42228800	6.37555300	-1.87518700
H	-8.83039100	7.52673400	-3.05983000

H	-9.77105700	5.50052900	-4.19790900
H	-8.03629200	5.62455400	-4.52155200
H	-8.63083400	4.46440600	-3.32831300

1b-TS5

Ir	-1.52754600	0.70020100	0.11737000
Cl	-1.65064500	2.01844600	-1.87607700
C	-3.14669700	1.32426000	1.38515300
C	-2.15552700	0.60262000	2.18654200
C	-2.06372900	-0.72929300	1.66365900
C	-3.00997400	-0.83335700	0.55341800
C	-3.71374000	0.41087800	0.42313900
C	-3.56802400	2.74321800	1.58067100
H	-3.87749100	3.18635500	0.63283600
H	-4.41091300	2.79511200	2.28096600
C	-1.37385100	1.15598600	3.33282800
H	-1.30440400	2.24367300	3.27274000
H	-0.35655700	0.75882600	3.33542000
H	-1.84989500	0.89624100	4.28592200
C	-1.18806200	-1.82443700	2.18169200
H	-0.26945700	-1.41761200	2.60870600
H	-0.90405700	-2.51398200	1.38383500
H	-1.70446200	-2.40039600	2.95916100
C	-3.23230400	-2.06101500	-0.26903100
H	-3.98017600	-2.70713800	0.20717200
H	-2.31001200	-2.63604900	-0.37540500
H	-3.58664800	-1.80762500	-1.26947700
C	-4.77261600	0.75208700	-0.57570300
H	-4.82467600	0.00163100	-1.36616000
H	-4.55594500	1.71041300	-1.05325900
H	-5.75623800	0.81001300	-0.09554300
H	-2.75172700	3.34627700	1.98255400
C	1.13182700	0.71642300	-0.85094000
H	0.69117400	-0.99474600	-3.59294500
C	0.89130900	-0.41357000	-1.55709300
C	1.49813800	-0.81576900	-2.86608000
H	0.17536400	-1.11162400	-1.12021000
H	2.09503000	0.00161300	-3.28426800
O	0.43873600	0.98413200	0.27406900
C	2.36446200	-2.08387800	-2.77117400
C	2.14296400	1.77531900	-1.18565300
H	2.84831600	1.46183700	-1.95741100
H	1.62183600	2.67700300	-1.52482900
H	2.70141300	2.03742200	-0.28103200
H	3.18844600	-1.89956900	-2.06923500
H	1.76877700	-2.89673300	-2.33210700
C	2.92626100	-2.53556500	-4.12143300
H	3.51412800	-1.71793000	-4.56233200
H	2.09577000	-2.71892300	-4.81807900
C	3.79810300	-3.79150600	-4.03069900
H	3.21029300	-4.60793200	-3.58962500
H	4.62719200	-3.60519600	-3.33479200
C	4.35572100	-4.23490900	-5.38535400
H	3.54723900	-4.46155800	-6.08963500
H	4.97136600	-3.44738200	-5.83457000
H	4.97740000	-5.13131200	-5.29171300

1b-Int6a

C	-2.74934300	12.02249200	-0.43092000
C	-3.49740100	12.65092900	0.49478200
O	-1.40580500	11.88260000	-0.27250200
Ir	-0.61271700	10.49920500	0.84508900
Cl	1.46594100	11.37888400	0.09649000
C	0.18648100	8.71709700	1.90548100
C	-0.88444900	8.36995600	0.99888500
C	-2.11218800	9.01872900	1.44905900
C	-1.76546700	9.83958000	2.56856200
C	-0.33507800	9.66973600	2.83545000
C	1.60548900	8.25311100	1.82848400
H	2.29296600	9.07678400	2.03005800
H	1.78924900	7.45330600	2.55530300
C	-0.77811100	7.42092200	-0.14967700
H	0.21742800	7.45534200	-0.59509500
H	-1.50315800	7.66159600	-0.92960700
H	-0.96913400	6.39509800	0.18883400
C	-3.48249300	8.84945100	0.87847300

H	-3.44306100	8.41667500	-0.12269000
H	-3.98727400	9.81612700	0.80755000
H	-4.08602900	8.18654800	1.50935700
C	-2.70746000	10.66810900	3.37772300
H	-3.07851200	10.08686900	4.23096700
H	-3.56104200	10.98871800	2.78008600
H	-2.21481000	11.56113400	3.76865300
C	0.42642900	10.33349700	3.93595800
H	0.02108300	11.32381400	4.15349600
H	1.47607000	10.45455500	3.66393800
H	0.37286700	9.73433600	4.85330400
H	1.84264700	7.87218500	0.83401000
C	-4.95921100	12.98805700	0.41532500
H	-5.38146500	12.67627200	-0.54597600
H	-5.08329000	14.08083200	0.45645000
H	-2.96582700	12.99404400	1.38127900
C	-3.21974300	11.47444400	-1.75084900
H	-4.28882200	11.61539500	-1.91759400
H	-2.66734100	11.95510200	-2.56478500
H	-2.99507300	10.40253800	-1.80446000
C	-5.79591200	12.37219600	1.54989200
C	-7.27187500	12.77427200	1.49913100
H	-5.37062500	12.67114200	2.51822100
H	-5.71545700	11.27701100	1.50506100
C	-8.11094500	12.16136200	2.62422400
H	-7.35066600	13.86962000	1.54198300
H	-7.69474600	12.47869100	0.52850400
C	-9.58594900	12.56457700	2.55834800
H	-8.02629500	11.06686400	2.58249300
H	-7.68972900	12.46163100	3.59315400
H	-10.16373600	12.11458000	3.37199000
H	-9.70012200	13.65207400	2.62937100
H	-10.03846800	12.24742400	1.61206400

1b-Int7a

C	-2.76777800	12.28075000	-0.42406000
C	-3.52887800	12.89787600	0.47750500
O	-1.38347200	12.13844400	-0.08154600
H	-0.79053500	12.39538100	-0.81557200
Ir	-0.55181000	10.39318500	0.81463000
Cl	1.20823300	10.94922200	-0.60808400
C	0.17745400	8.68389800	1.92313300
C	-0.91952300	8.29295100	1.07055100
C	-2.11671000	9.02196900	1.49639500
C	-1.75360100	9.86551600	2.59236400
C	-0.33203500	9.67793200	2.84507000
C	1.56903200	8.14356400	1.87711000
H	2.28803100	8.86437400	2.26842900
H	1.63352500	7.23208000	2.48162200
C	-0.85909000	7.28248200	-0.02265300
H	0.14166100	7.22069800	-0.45154100
H	-1.56387700	7.51332200	-0.82337900
H	-1.11940700	6.29660500	0.38180300
C	-3.47992300	8.89004300	0.90487200
H	-3.43850400	8.48441600	-0.10683700
H	-3.98695400	9.85624500	0.87007700
H	-4.08566200	8.20992000	1.51465800
C	-2.66901700	10.75035900	3.37077500
H	-3.03835700	10.21179100	4.25124100
H	-3.53116700	11.05477800	2.77703100
H	-2.15783700	11.64933700	3.72107500
C	0.45809600	10.36977800	3.90102700
H	0.05183000	11.35753200	4.12440200
H	1.50314600	10.48127300	3.60872900
H	0.42516800	9.77487000	4.82232500
H	1.86558100	7.90122200	0.85559200
C	-5.00722700	13.13357800	0.38956800
H	-5.40243800	12.80933400	-0.57798200
H	-5.18798900	14.21485100	0.44928900
H	-3.02582900	13.26615100	1.36954800
C	-3.09988600	11.67486500	-1.74582100
H	-4.16042300	11.78208300	-1.97296200
H	-2.53503900	12.15407100	-2.55512600
H	-2.84512200	10.60863200	-1.75711600
C	-5.78793300	12.44281000	1.52259300
C	-7.28799800	12.74647300	1.48306100

H	-5.37708600	12.75602800	2.49225800
H	-5.63716600	11.35589000	1.45672900
C	-8.07363200	12.04270000	2.59399900
H	-7.43828400	13.83162300	1.55916200
H	-7.69272800	12.44975100	0.50583600
C	-9.57201300	12.34967900	2.54632200
H	-7.91659300	10.95811400	2.51564800
H	-7.66611100	12.34133700	3.56910200
H	-10.10962100	11.83513800	3.34790800
H	-9.75779400	13.42342400	2.65551200
H	-10.00942400	12.03255000	1.59348200

1b-TS6a

C	-4.65788900	9.49666400	-0.11124900
C	-5.39078400	10.68029200	-0.13923000
O	-3.78412600	9.31916400	0.85417700
H	-4.21609200	9.97005200	2.42455500
O	-4.71812300	10.25632600	3.22597500
H	-6.16753500	10.08485300	2.61312900
O	-6.91096200	9.94761100	1.92506100
H	-6.34045600	10.21942400	0.93943100
C	-8.09886600	10.80293100	2.19220600
H	-8.77751200	10.55563300	1.37240100
C	-4.35905900	11.65397100	3.47990300
H	-4.82516700	12.26636000	2.69628600
C	-7.72485200	12.25089900	2.13444200
C	-8.10889300	13.07349500	1.15418000
H	-7.11692000	12.63105800	2.95413900
H	-8.73803500	12.67521200	0.35611500
C	-2.86974900	11.80868100	3.40278000
C	-2.22610900	12.23295700	2.31247300
H	-2.30716400	11.51512300	4.28775000
H	-2.80975700	12.52158800	1.43628300
Ir	-2.25239000	7.95215400	1.00986500
Cl	-1.52083200	8.59195000	-1.12156800
C	-1.04420900	6.17225000	1.36296300
C	-2.45386100	5.92083400	1.64504900
C	-2.86390100	6.76357600	2.75816000
C	-1.74350300	7.58025000	3.10741700
C	-0.61459100	7.21076600	2.24459400
C	-0.21651200	5.48707500	0.32587900
H	0.52105900	6.16998100	-0.09815100
H	0.30942200	4.63236800	0.76549900
C	-3.29822100	4.89548700	0.96771900
H	-3.02081100	4.78241400	-0.08156600
H	-4.35856400	5.14848200	1.02175100
H	-3.15848100	3.92567800	1.46094700
C	-4.21530000	6.79642100	3.39605600
H	-4.99117800	6.48818400	2.69234400
H	-4.46022600	7.80028600	3.74909400
H	-4.24695900	6.11250200	4.25167600
C	-1.69291900	8.58540500	4.20706300
H	-1.33877900	8.10436800	5.12686600
H	-2.67797500	9.01290400	4.39553000
H	-1.00792000	9.40026100	3.96751100
C	0.75550100	7.79397800	2.33113300
H	0.72378200	8.85142800	2.59774500
H	1.29038700	7.69932200	1.38609300
H	1.32818700	7.26723700	3.10381200
H	-0.83549100	5.12355900	-0.49564800
C	-6.18537400	11.15367500	-1.34120800
H	-6.98282100	11.82104200	-0.99778500
H	-6.68645400	10.31795100	-1.84209300
H	-4.94159300	11.48586700	0.44605300
C	-4.92527800	8.34761400	-1.04057500
H	-5.90702700	8.42085400	-1.50803500
H	-4.16302700	8.33458700	-1.82449400
H	-4.84921000	7.40259800	-0.49504600
C	-5.30776400	11.90999200	-2.35179200
C	-6.09669800	12.51280700	-3.51547600
H	-4.53319100	11.23202900	-2.73372300
H	-4.77317900	12.71135100	-1.82409500
C	-5.21920800	13.31624400	-4.47997100
H	-6.61222700	11.71394600	-4.06645000
H	-6.88688900	13.16540300	-3.11836300
C	-6.01414200	13.95636900	-5.61972400

H	-4.69106600	14.09656300	-3.91623700
H	-4.44212900	12.65978500	-4.89285300
H	-5.36261600	14.52079100	-6.29335600
H	-6.53293600	13.19762100	-6.21577500
H	-6.77211300	14.64719400	-5.23312800
C	-8.68011000	10.35524500	3.52376800
H	-9.60856800	10.89839200	3.71804100
H	-8.89423900	9.28384300	3.51452200
H	-7.98809700	10.56809100	4.34513800
C	-7.70084100	14.50781100	1.01063600
C	-6.80832200	14.70617300	-0.22942700
H	-7.17193400	14.84283300	1.91067100
H	-8.59454100	15.13797200	0.91092500
C	-6.39752900	16.16030200	-0.47073500
H	-5.90996100	14.08395100	-0.12186000
H	-7.33439900	14.32870500	-1.11598700
C	-5.51374200	16.31378200	-1.71112200
H	-7.29822000	16.77797200	-0.57873500
H	-5.86857000	16.54134200	0.41254400
H	-5.22765100	17.35709000	-1.87211000
H	-4.59312100	15.72675000	-1.61509100
H	-6.03363100	15.96772100	-2.61129400
C	-4.93297000	12.01707300	4.83903700
H	-4.73361400	13.06819900	5.06328300
H	-6.01447600	11.85727200	4.87243800
H	-4.47538400	11.40267100	5.62001900
C	-0.73730200	12.28963800	2.15562300
C	-0.25351600	11.34324100	1.04385200
H	-0.25240500	12.03442100	3.10658600
H	-0.42635600	13.31425700	1.91017500
C	1.26747100	11.32701500	0.87572100
H	-0.61222000	10.32748400	1.25656500
H	-0.72678300	11.61947200	0.09348000
C	1.72247100	10.46145700	-0.30158600
H	1.62790400	12.35457600	0.73767100
H	1.73262700	10.97101700	1.80607200
H	2.81371400	10.42109300	-0.36915800
H	1.34416700	9.43809200	-0.21386500
H	1.34078600	10.85747100	-1.24793000

1b-TS4’

Ir	-0.31349900	-0.17106900	0.00474400
Cl	0.90928100	1.66775800	-1.03834000
C	-1.73040000	1.19749800	1.13480500
C	-1.58184100	0.01787300	1.90386500
C	-2.01096200	-1.11376500	1.08917000
C	-2.53565900	-0.59221500	-0.15296300
C	-2.28651200	0.81567200	-0.16980600
C	-1.40865600	2.60183700	1.53298600
H	-0.80138100	3.08447400	0.76281200
H	-2.32651500	3.18619400	1.66953200
C	-1.07235100	-0.08143500	3.30836700
H	-0.42776400	0.76305700	3.55796500
H	-0.49723800	-0.99768100	3.45911900
H	-1.90830000	-0.09261500	4.01843300
C	-2.11670600	-2.52955800	1.56404700
H	-1.28751100	-2.78799800	2.22700400
H	-2.10498200	-3.23048400	0.72636900
H	-3.04915300	-2.68874600	2.11963500
C	-3.24692000	-1.37510600	-1.20855600
H	-4.32582000	-1.37541400	-1.00999400
H	-2.91423500	-2.41353300	-1.23566300
H	-3.08941800	-0.94447700	-2.19932900
C	-2.66608700	1.78776900	-1.24036200
H	-2.83386700	1.28200700	-2.19334300
H	-1.87056100	2.52110600	-1.38935000
H	-3.58586400	2.32043900	-0.96891400
H	-0.84658100	2.63073700	2.46847600
C	2.85024500	-1.52550200	0.02522400
H	1.10292800	-0.56723700	0.78279400
C	1.34137700	-1.69301600	-0.13106000
C	0.62512700	-1.46278600	-1.38766100
H	1.03151100	-2.56493100	0.44355600
H	1.16675200	-0.87494900	-2.12657200
O	3.41473500	-2.21570900	0.85501000
C	3.57991200	-0.56098100	-0.87022000

H	4.62517800	-0.51162200	-0.56374000
H	3.52024500	-0.90492000	-1.90887600
H	3.10697500	0.42543900	-0.84139900
C	-0.14796800	-2.61341100	-1.98952900
H	-0.70144800	-3.14401500	-1.20426200
H	-0.89267500	-2.22458300	-2.69427700
C	0.76503200	-3.61148800	-2.71907500
H	1.51443600	-3.99635100	-2.01365500
H	1.32586200	-3.08131100	-3.50081000
C	0.00268900	-4.78380700	-3.34287400
H	-0.74678500	-4.39449300	-4.04509300
H	-0.55912000	-5.30718400	-2.55708000
C	0.91884100	-5.77445600	-4.06484800
H	1.65734300	-6.20074000	-3.37671200
H	1.46888400	-5.28281100	-4.87493000
H	0.35147600	-6.60275500	-4.50096300

1b-Int6’

Ir	-0.78117400	0.33944400	-0.67892500
Cl	0.89932800	2.10815000	-0.88849100
C	-1.91844900	1.34267000	1.08523700
C	-1.02844100	0.22565300	1.43927300
C	-1.57479600	-0.97770600	0.84211800
C	-2.64874300	-0.57197000	-0.02651200
C	-2.88355600	0.86395400	0.18468600
C	-1.70053300	2.74916700	1.54168600
H	-0.69584200	3.08461000	1.26567600
H	-2.41887000	3.43423300	1.08749900
C	0.06495200	0.31263500	2.45670700
H	0.64048200	1.23011100	2.31504400
H	0.75771500	-0.52671800	2.36543500
H	-0.34149900	0.31066100	3.47613800
C	-1.14063000	-2.38267500	1.10874800
H	-0.10412900	-2.42215300	1.44735700
H	-1.22739900	-3.00663300	0.21622500
H	-1.76648300	-2.82694600	1.89220900
C	-3.53463700	-1.47624400	-0.82503000
H	-4.41728800	-1.77839700	-0.24678300
H	-3.00299100	-2.38236700	-1.12489600
H	-3.88483100	-0.97833100	-1.73283200
C	-3.93933400	1.65031600	-0.52612600
H	-3.95968700	1.40045800	-1.59094000
H	-3.76101700	2.72364300	-0.44152400
H	-4.93325000	1.43487700	-0.11516100
H	-1.80148600	2.82160300	2.63082000
C	-0.18465100	0.20290500	-3.54520400
H	1.74140600	0.31763200	-2.79652200
C	0.98502900	-0.47365400	-2.90992600
C	0.61445100	-1.00265300	-1.50750300
H	1.40839300	-1.22581200	-3.58877800
H	0.09188000	-1.96329500	-1.63535200
O	-1.09706400	0.63755800	-2.82645500
C	-0.25877100	0.38793800	-5.03084800
H	-1.14486100	0.96111000	-5.30516300
H	0.64230100	0.90376000	-5.38057100
H	-0.27680500	-0.59048600	-5.52453200
C	1.86019600	-1.23904600	-0.65315300
H	2.37895400	-0.28431800	-0.51066600
H	1.54906200	-1.56752700	0.34562400
C	2.83247700	-2.28424700	-1.22029300
H	3.22848400	-1.94333500	-2.18658500
H	2.28708300	-3.21720900	-1.42683100
C	4.00696600	-2.58260300	-0.28312700
H	3.61631300	-2.93498000	0.68131200
H	4.54228000	-1.64807100	-0.06956100
C	4.98197300	-3.61665700	-0.85079400
H	5.41502600	-3.27146100	-1.79658200
H	4.47560600	-4.56836700	-1.04920100
H	5.80761700	-3.81583200	-0.15980000

1b-Int7’

Ir	-0.25103100	-0.19595700	0.19045600
Cl	1.41005700	-0.68048600	1.83451800
C	-1.80211700	1.37528500	0.81536300
C	-2.10628000	0.20406300	1.53287100
C	-2.24000600	-0.87395500	0.55732700

C	-2.18577600	-0.30506900	-0.78774500
C	-1.83108100	1.06958800	-0.62953100
C	-1.47046200	2.71983600	1.37035000
H	-0.64541200	3.17850600	0.81952600
H	-2.33637600	3.38681600	1.28349400
C	-2.15858800	0.02003500	3.01493100
H	-1.78101500	0.89768200	3.54059100
H	-1.55686400	-0.83739500	3.32754300
H	-3.18995800	-0.15268500	3.34167500
C	-2.59070000	-2.28496000	0.89141100
H	-2.12955100	-2.59502700	1.83100900
H	-2.27535400	-2.97123600	0.10254900
H	-3.67786900	-2.38212700	1.00093400
C	-2.55332100	-0.99807400	-2.05868300
H	-3.61582700	-0.83920800	-2.27648700
H	-2.39670700	-2.07715900	-1.99039900
H	-1.98026800	-0.61802000	-2.90623600
C	-1.73535700	2.09027900	-1.71471000
H	-1.42492200	1.64515500	-2.66070800
H	-1.02890900	2.88320300	-1.46399700
H	-2.71742500	2.55456700	-1.86595000
H	-1.18621500	2.66322000	2.42149200
C	1.10886500	-3.47961300	-0.65140600
H	-0.16235100	-2.56387200	-2.00141700
C	0.86432100	-2.40003300	-1.63822700
C	1.11336200	-0.94543600	-1.24211300
H	1.48169200	-2.69498600	-2.50859600
H	2.11102500	-0.89988400	-0.78278000
O	1.42984700	-3.28823200	0.55727100
C	1.03372600	-4.90516500	-1.06952300
H	0.70185900	-5.53402600	-0.24110200
H	0.40087700	-5.03624800	-1.94824500
H	2.05033000	-5.22574000	-1.33741800
C	1.16970900	-0.06277000	-2.49788900
H	1.83625600	-0.52506700	-3.24372400
H	0.18141600	-0.01145500	-2.97273500
C	1.68120200	1.35027300	-2.20204800
H	2.69744800	1.28366700	-1.79173100
H	1.07046200	1.79899200	-1.40599300
C	1.68187600	2.27195100	-3.42341700
H	0.66967400	2.31506600	-3.84815000
H	2.31835800	1.83595100	-4.20409500
C	2.16205300	3.68700700	-3.09523900
H	3.18128500	3.67498400	-2.69494900
H	1.51846100	4.15859900	-2.34328500
H	2.15946600	4.32618600	-3.98247600
H	1.48544900	-2.31432900	0.90183000

1b-TS6’

Ir	-0.34098400	-0.53511100	0.38336600
Cl	1.24097600	-0.74309100	2.09041900
C	-1.63280200	1.35893300	0.51924300
C	-2.00794400	0.48434200	1.56217900
C	-2.38854200	-0.79367700	0.94828200
C	-2.35524100	-0.62735800	-0.50515900
C	-1.80964800	0.66259500	-0.76897000
C	-1.10699900	2.74805600	0.65871300
H	-0.35493900	2.96964800	-0.10166700
H	-1.92642600	3.46539600	0.52848900
C	-1.97892700	0.75174100	3.02929000
H	-1.34645700	1.60721300	3.26762500
H	-1.59474600	-0.11073000	3.57705200
H	-2.99276500	0.96261500	3.38804800
C	-2.93367400	-1.97062100	1.68202000
H	-2.50811400	-2.03896800	2.68408100
H	-2.71894200	-2.90141000	1.15676700
H	-4.02144900	-1.86506600	1.77829700
C	-2.86642600	-1.61154000	-1.50521900
H	-3.93381900	-1.43481300	-1.67955900
H	-2.75283300	-2.63493800	-1.14708700
H	-2.35478500	-1.51382900	-2.46481500
C	-1.60132200	1.28955200	-2.10810400
H	-1.53034700	0.53671900	-2.89455500
H	-0.69354000	1.89627600	-2.13195600
H	-2.44647900	1.94563700	-2.34727100
H	-0.66379400	2.91432700	1.64109400

C	-0.19064500	-4.01917800	-1.05019200
H	-0.09995000	-2.57979400	-2.59887700
C	0.56033400	-2.93253000	-1.79296900
C	1.01442800	-1.77688600	-0.90612400
H	1.42877500	-3.37411800	-2.29691600
H	1.78524400	-2.12145800	-0.21359600
O	-0.68705100	-3.75203900	0.04176700
C	-0.31746900	-5.37599800	-1.67121400
H	-1.06971100	-5.96939700	-1.15126400
H	-0.55222200	-5.29823100	-2.73747400
H	0.65213800	-5.88427700	-1.59606100
C	1.54938200	-0.62709900	-1.78245000
H	2.34847000	-1.05512800	-2.40740100
H	0.76365800	-0.30615000	-2.47576300
C	2.12303000	0.58928300	-1.04278100
H	2.90289300	0.26664900	-0.34427800
H	1.35448300	1.05936500	-0.40436000
C	2.66207600	1.65226600	-2.00217100
H	1.86690700	1.95025100	-2.69987500
H	3.45065600	1.20265500	-2.61965000
C	3.20641400	2.88344100	-1.27593900
H	4.02112800	2.61209000	-0.59717500
H	2.42621700	3.36787800	-0.67687900
H	3.59073600	3.62412300	-1.98218800
H	-0.22245700	-2.16400700	0.08480900

Int2-1

Ir	-0.58538100	0.43492500	0.10225800
Cl	1.13843500	1.62510700	1.18196700
Cl	-0.38295100	-3.11428200	-1.90465500
C	-2.33394700	0.20878700	-1.22690400
C	-2.59543300	-0.48313700	-0.00426400
C	-2.50940300	0.49173700	1.08793200
C	-2.23335000	1.78777500	0.53462600
C	-2.05951600	1.60407900	-0.89228800
C	-2.36416500	-0.36308400	-2.60303800
H	-1.98829200	-1.39022500	-2.60588700
H	-1.74681100	0.22555500	-3.28397400
C	-2.95827000	-1.92256000	0.13676800
H	-4.02831200	-2.04868000	-0.07041500
H	-2.38289300	-2.53892300	-0.56095000
H	-2.76655600	-2.28157600	1.14967600
C	-2.71945400	0.17685400	2.53094800
H	-2.32278400	-0.80977900	2.77792000
H	-2.23234700	0.91229200	3.17247800
H	-3.79270500	0.17596200	2.75667700
C	-2.11090300	3.07778200	1.27843800
H	-3.05990100	3.62496200	1.24743500
H	-1.84408300	2.90729800	2.32222700
H	-1.33187200	3.70673900	0.84434800
C	-1.72340000	2.66528300	-1.88504600
H	-1.29645000	3.54293700	-1.39799200
H	-1.00221300	2.29136600	-2.61591300
H	-2.62898300	2.97237800	-2.42198900
H	-3.39549300	-0.35141800	-2.97738200
C	1.98466500	-0.41854100	-1.35320200
H	2.26335800	0.52168400	-0.88075100
O	0.85457200	-0.91920000	-0.53983600
H	0.41768200	-1.74753600	-1.00940500
C	1.48428800	-0.16204500	-2.74710600
C	1.44678500	1.05903300	-3.28325300
H	1.13374400	-1.03175500	-3.29754400
H	1.09702600	1.22526700	-4.29819000
H	1.79217200	1.93080300	-2.73219500
C	3.15304400	-1.36959100	-1.26915000
C	4.32784000	-0.92959500	-0.64871900
C	3.10394800	-2.66020400	-1.81356200
C	5.44515800	-1.76074100	-0.57567400
H	4.36287900	0.06783000	-0.21880400
C	4.21984800	-3.49125800	-1.73219600
H	2.18668600	-3.02404800	-2.26566400
C	5.39257900	-3.04446300	-1.11950600
H	6.35129400	-1.40785800	-0.09224900
H	4.17074800	-4.49318600	-2.14789200
H	6.25970700	-3.69600500	-1.06195100

TS2-1

**Ir**	**-0.86010900**	**0.26257800**	**0.67275500**
**Cl**	**1.42450800**	**-0.88545200**	**-2.29679800**
**Cl**	**0.30705000**	**-1.14310100**	**2.17994600**
**C**	**-2.51612600**	**1.51126800**	**0.00007900**
**C**	**-2.93622900**	**0.66776100**	**1.12508900**
**C**	**-2.80105100**	**-0.70382800**	**0.73922800**
**C**	**-2.23836800**	**-0.72264500**	**-0.61057400**
**C**	**-2.12371100**	**0.65662700**	**-1.07667000**
**C**	**-2.53281400**	**3.00475300**	**-0.00922900**
**H**	**-1.81109600**	**3.39938100**	**-0.72604000**
**H**	**-3.52724500**	**3.37493200**	**-0.28555300**
**C**	**-3.44982400**	**1.17830500**	**2.43165700**
**H**	**-4.52222200**	**1.39450200**	**2.35545700**
**H**	**-2.94063000**	**2.09999200**	**2.72074400**
**H**	**-3.30330600**	**0.44648700**	**3.22723400**
**C**	**-3.13663900**	**-1.91095300**	**1.55133000**
**H**	**-3.19558100**	**-1.66960200**	**2.61339200**
**H**	**-2.37176900**	**-2.68026600**	**1.43210800**
**H**	**-4.10146200**	**-2.32288800**	**1.23418700**
**C**	**-1.91395200**	**-1.92690500**	**-1.42539600**
**H**	**-2.68379500**	**-2.06584700**	**-2.19494800**
**H**	**-1.87766600**	**-2.82387900**	**-0.80578100**
**H**	**-0.94099200**	**-1.80305900**	**-1.91494300**
**C**	**-1.61251400**	**1.05622400**	**-2.42004600**
**H**	**-0.71332500**	**0.48379800**	**-2.67606300**
**H**	**-1.36531500**	**2.11853900**	**-2.45146200**
**H**	**-2.37909300**	**0.85967400**	**-3.17876700**
**H**	**-2.28487500**	**3.40694600**	**0.97537600**
**C**	**5.08360700**	**3.60519700**	**-1.28215900**
**C**	**4.07960600**	**3.01161700**	**-2.04585400**
**C**	**3.03753300**	**2.31474200**	**-1.42994800**
**C**	**3.00342600**	**2.20318900**	**-0.03824500**
**C**	**4.01570600**	**2.79582300**	**0.72727600**
**C**	**5.05044500**	**3.49430100**	**0.10997300**
**H**	**5.89196000**	**4.14488800**	**-1.76647400**
**H**	**4.10639800**	**3.08088000**	**-3.12914000**
**H**	**2.27803900**	**1.82991100**	**-2.03154800**
**H**	**3.98832900**	**2.70958900**	**1.81058700**
**H**	**5.83121100**	**3.94817200**	**0.71317800**
**C**	**1.88763300**	**1.48261700**	**0.69145000**
**H**	**2.29673300**	**0.69537800**	**1.33436600**
**C**	**1.05664600**	**2.44072300**	**1.51258600**
**C**	**0.70631600**	**2.24075600**	**2.78797500**
**H**	**0.71757000**	**3.32356800**	**0.97313900**
**H**	**0.08381100**	**2.95443000**	**3.32018500**
**H**	**1.02903900**	**1.35944100**	**3.33128200**
**O**	**0.93900700**	**0.90268300**	**-0.22175500**
**H**	**1.28225600**	**0.17924700**	**-0.95018900**

HCl

**Cl**	**0.00000000**	**0.00000000**	**0.07142000**
**H**	**0.00000000**	**0.00000000**	**-1.21413500**

Int2-2

**Ir**	**-0.21984900**	**0.99887200**	**-0.34641200**
**Cl**	**1.57566700**	**0.33005400**	**1.06523000**
**C**	**-2.29685600**	**1.59159200**	**-0.52813200**
**C**	**-1.92486500**	**0.81628200**	**-1.67143000**
**C**	**-1.48765100**	**-0.48961700**	**-1.18048500**
**C**	**-1.71206500**	**-0.54515200**	**0.25214100**
**C**	**-2.15664100**	**0.74311500**	**0.66701500**
**C**	**-2.79774400**	**2.99876700**	**-0.53620900**
**H**	**-2.41667600**	**3.54375000**	**-1.40156000**
**H**	**-2.48237200**	**3.53372500**	**0.36212800**
**C**	**-1.93528100**	**1.25849000**	**-3.09823500**
**H**	**-2.92143000**	**1.09416700**	**-3.54823700**
**H**	**-1.68975300**	**2.31964900**	**-3.17811400**
**H**	**-1.19419300**	**0.70597400**	**-3.67799300**
**C**	**-0.98568600**	**-1.60711100**	**-2.03580200**
**H**	**-0.34022400**	**-1.23452200**	**-2.83446600**
**H**	**-0.40762700**	**-2.31978500**	**-1.44574300**
**H**	**-1.82878000**	**-2.14173500**	**-2.49130800**
**C**	**-1.40738800**	**-1.71707600**	**1.12908800**
**H**	**-2.20854800**	**-2.46284500**	**1.07023700**
**H**	**-0.46899500**	**-2.19000900**	**0.83188700**

H	-1.29773000	-1.41093600	2.17018600
C	-2.46523600	1.17266700	2.06414000
H	-1.87056300	0.61174000	2.78669100
H	-2.24546700	2.23309100	2.20452800
H	-3.52655900	1.01289300	2.29031000
H	-3.89386800	3.01473000	-0.57384500
C	2.25261900	2.06866800	-1.60329200
H	2.79734300	1.82460600	-0.68620800
O	0.86884700	2.29254900	-1.33991100
C	2.81346200	3.33914800	-2.18545200
C	3.86801900	3.98147200	-1.68634400
H	2.29916000	3.72268000	-3.06549700
H	4.25246600	4.89176800	-2.13693100
H	4.38701800	3.61391900	-0.80451600
C	2.36344600	0.89441300	-2.56531000
C	3.05876300	-0.26427800	-2.21090200
C	1.68817400	0.92938300	-3.79347400
C	3.07828700	-1.37051200	-3.06395200
H	3.55937600	-0.30663400	-1.24913400
C	1.70768800	-0.17121700	-4.64850600
H	1.13265500	1.82091000	-4.06643700
C	2.40291400	-1.32888000	-4.28373300
H	3.61782400	-2.26676100	-2.77126500
H	1.18548400	-0.12790900	-5.60067700
H	2.41811400	-2.18847300	-4.94753100

TS3-1

Ir	0.50326300	0.36258800	-0.31785500
Cl	1.32498300	2.52947700	-0.92676900
C	0.92384300	-1.42763100	0.88209300
C	0.40315200	-1.87886900	-0.37430200
C	1.33607400	-1.47905700	-1.42570700
C	2.39678600	-0.74609500	-0.83173300
C	2.12581200	-0.64681100	0.60615900
C	0.34094600	-1.70770700	2.22831100
H	-0.75025300	-1.67686600	2.20421100
H	0.68717200	-0.98416200	2.96775800
C	-0.78510400	-2.76110700	-0.57722900
H	-0.45602800	-3.80096100	-0.68746600
H	-1.46635000	-2.71873400	0.27347800
H	-1.33702400	-2.49461800	-1.48163800
C	1.16339100	-1.78925100	-2.87532200
H	0.11057700	-1.77197800	-3.16543600
H	1.70627700	-1.08462200	-3.50628600
H	1.54662100	-2.79541900	-3.08436400
C	3.58408000	-0.15399800	-1.51317700
H	4.46933400	-0.76312900	-1.29769600
H	3.45096700	-0.11578500	-2.59458000
H	3.77119900	0.86190400	-1.16022300
C	3.05905300	-0.05653900	1.61192800
H	3.53630100	0.84250400	1.21860500
H	2.53510400	0.21298000	2.53044700
H	3.84222600	-0.78033100	1.86514700
H	0.64714000	-2.70500900	2.56331800
C	-1.55939500	1.30994300	0.05718400
H	-0.16374800	1.12105900	0.94380200
O	-1.34434300	1.23623100	-1.33412100
C	-1.77541600	2.69754400	0.57829400
C	-1.02356700	3.25807000	1.61293900
C	-2.83698400	3.42088400	0.01121300
C	-1.33089100	4.53216000	2.08580800
H	-0.17869000	2.71473200	2.02526300
C	-3.13155100	4.69866600	0.47953300
H	-3.43613100	2.97789800	-0.77851600
C	-2.38137400	5.25409700	1.51804100
H	-0.73868300	4.96589300	2.88433500
H	-3.94983200	5.25697300	0.03725900
H	-2.61249000	6.25013700	1.88087700
C	-2.46947200	0.23231900	0.49704200
C	-3.14394600	0.26532700	1.65038100
H	-2.55961600	-0.60367900	-0.18926000
H	-3.80159500	-0.54924900	1.93306000
H	-3.06721600	1.10494300	2.33352700
H	-0.95451000	2.09098500	-1.62968700

TS3-2

Ir	0.54645500	0.27922300	-0.08409800
Cl	1.46317900	2.52464200	-0.00179800
C	1.32596200	-1.48544000	0.98008100
C	0.51940100	-1.93026400	-0.14406200
C	1.14660600	-1.44202900	-1.35266500
C	2.29839300	-0.66974800	-0.98454700
C	2.41367100	-0.70209100	0.47581200
C	1.06603900	-1.81771900	2.41560800
H	-0.00368800	-1.92185800	2.60871700
H	1.45289000	-1.03964600	3.07574500
C	-0.63936600	-2.87302400	-0.07705100
H	-0.29273200	-3.91356400	-0.10511800
H	-1.21062100	-2.73822600	0.84429300
H	-1.31766700	-2.72719000	-0.92099000
C	0.61780100	-1.65468900	-2.73473400
H	-0.46910300	-1.54496500	-2.75178800
H	1.03387700	-0.92604600	-3.43172900
H	0.87042300	-2.65922400	-3.09404200
C	3.26501600	0.01931200	-1.89210300
H	4.20483900	-0.54321000	-1.94858300
H	2.86368900	0.11545800	-2.90252500
H	3.47922700	1.02455900	-1.52211300
C	3.51799000	-0.06761900	1.25697900
H	3.70910300	0.94288300	0.88973100
H	3.25847000	0.01068700	2.31432400
H	4.43838000	-0.65753700	1.17077500
H	1.55152000	-2.76435400	2.68145700
C	-1.60655500	1.22046000	0.13812800
H	-0.59818900	0.89112600	1.04604900
O	-1.10128500	1.10163200	-1.09730500
C	-1.85662800	2.62386200	0.63340600
C	-2.05792600	2.89522900	1.99070300
C	-1.89226400	3.66485000	-0.29539200
C	-2.31693000	4.19710700	2.41373100
H	-1.99336200	2.08965900	2.71684200
C	-2.16096500	4.96537600	0.12758800
H	-1.68789000	3.43608800	-1.33411800
C	-2.37626300	5.23418900	1.48008100
H	-2.46388200	4.40397400	3.46957100
H	-2.18810400	5.77279800	-0.59799500
H	-2.57660600	6.24972500	1.80873600
C	-2.63467500	0.17044900	0.46527900
C	-3.78513400	0.35667200	1.11328100
H	-2.39434200	-0.79765600	0.03735400
H	-4.49013900	-0.46035600	1.23336700
H	-4.06719600	1.31823200	1.52684600

TS6-1

Ir	2.78048400	1.55571700	-3.40217100
Cl	4.16289300	-0.12357000	-2.44746500
C	0.67492300	2.17047500	-3.43498800
C	1.21974400	2.40386100	-4.73822100
C	1.65239900	1.11308300	-5.26543400
C	1.34239300	0.08891300	-4.30944500
C	0.81589800	0.74364600	-3.14071100
C	-0.09090800	3.12811300	-2.58714700
H	0.10922900	2.98269500	-1.52471000
H	-1.16260900	2.95885100	-2.75218900
C	1.21686000	3.68929200	-5.49634500
H	0.36622100	3.71102200	-6.18864900
H	1.12805700	4.54270900	-4.82258400
H	2.14195200	3.80012400	-6.06636900
C	2.23333800	0.90033500	-6.61988100
H	2.78438200	-0.03955100	-6.67397000
H	1.41801800	0.86596200	-7.35432900
H	2.91785700	1.71112600	-6.87435400
C	1.58460500	-1.37865100	-4.44351500
H	0.63429400	-1.91107700	-4.56495000
H	2.21090100	-1.59768500	-5.30892600
H	2.09506200	-1.76839700	-3.55946800
C	0.35495600	0.06559300	-1.89359600
H	0.95785700	-0.82031200	-1.68910800
H	0.42908800	0.73451800	-1.03392400
H	-0.69314400	-0.24159700	-1.99809300
H	0.13011000	4.16470000	-2.83348400
C	0.66458200	6.38323100	-0.41118700

C	0.97682500	6.80064000	-1.70696500
C	1.90718600	6.09284900	-2.46230500
C	2.53506200	4.95061600	-1.93538400
C	2.22855600	4.55203600	-0.62432900
C	1.29758000	5.26154100	0.12896300
H	-0.06073600	6.93478300	0.17906100
H	0.49689600	7.67891800	-2.12701900
H	2.16367300	6.40995100	-3.46569300
H	2.71271200	3.68659800	-0.18873100
H	1.06911900	4.94188800	1.14078600
C	3.51679400	4.22451700	-2.77529400
H	5.94069800	1.84653800	-2.28118400
C	4.11461600	2.98393800	-2.38903500
C	5.60266400	2.78999500	-2.70545300
H	3.89918600	2.73048400	-1.35255700
H	6.17665900	3.60625100	-2.24849500
H	5.80699200	2.77705000	-3.77486200
O	3.82011200	4.83875900	-3.87283600
Cl	4.68803300	3.18599400	-5.97789100
H	4.27119100	4.22267800	-4.61847200

TS7-1'

Ir	0.18285900	0.31378000	1.89678900
Cl	1.37343900	-0.99316400	0.22853900
C	-0.77975700	1.72351400	0.50966400
C	-0.47753100	2.39380000	1.74438000
C	-1.19442100	1.73366900	2.82559500
C	-1.94640700	0.64593800	2.23836600
C	-1.66407000	0.60708400	0.82088000
C	-0.27378900	2.05845000	-0.85454900
H	0.57614800	2.74118900	-0.80561300
H	0.05550300	1.15348700	-1.37036000
C	0.43342300	3.56572500	1.91516900
H	-0.14386600	4.49730700	1.93677300
H	1.15382400	3.62731600	1.09807200
H	0.99171600	3.49049200	2.85085600
C	-1.24572200	2.18012400	4.25210200
H	-0.30314900	2.64124800	4.55546500
H	-1.43829500	1.34090400	4.92411300
H	-2.04412800	2.91726600	4.40076000
C	-2.84785200	-0.28930900	2.97831200
H	-2.50229800	-0.45966600	3.99965700
H	-2.90934700	-1.25913300	2.48202100
H	-3.85918700	0.13105400	3.02867800
C	-2.23488000	-0.33595400	-0.18836700
H	-2.61296600	-1.24321000	0.28584400
H	-1.46674800	-0.63176200	-0.90535000
H	-3.06151700	0.13713600	-0.73127600
H	-1.06231200	2.53444300	-1.44937800
C	6.87619400	0.31914100	4.17711600
C	6.25656500	-0.87950000	4.53515400
C	4.91012700	-1.08261000	4.24533400
C	4.17294000	-0.08134200	3.58999900
C	4.80750800	1.12126500	3.22870300
C	6.15001300	1.31971900	3.52300800
H	7.92650500	0.47387600	4.40482900
H	6.82158100	-1.65583900	5.04038600
H	4.43882700	-2.01697800	4.52598300
H	4.22960100	1.87876200	2.71233400
H	6.63599200	2.24809600	3.24036700
C	2.75453200	-0.27040600	3.24933200
H	2.39477600	-2.27375800	2.74257700
C	2.04316500	-1.56543900	3.50315100
C	0.52698700	-1.47910700	3.45437600
H	2.33933500	-1.98885100	4.46904900
H	-0.00775900	-1.78194300	2.54250200
H	0.04783800	-0.80891000	4.16335200
O	2.14083400	0.68048500	2.71408100
Cl	0.17826700	-3.83988800	5.17745100
H	0.21984300	-2.52489100	4.06217900

Int8-1'

C	-5.80868100	5.21228700	-1.59142900
C	-6.28206700	6.52029200	-1.72294100
C	-5.40985400	7.59687700	-1.57744200
C	-4.06270100	7.36898100	-1.25202400

C	-3.58882100	6.04972500	-1.12587200
C	-4.45946000	4.97892300	-1.30885900
H	-6.48721200	4.37598500	-1.72556800
H	-7.32788600	6.70087700	-1.94735800
H	-5.78726200	8.60839100	-1.68074700
H	-2.53609700	5.86812300	-0.93957500
H	-4.08236600	3.96322100	-1.24731700
C	-3.13611300	8.49526800	-1.02752800
C	-3.15544900	9.68908100	-1.93821200
O	-2.29694900	8.47272500	-0.10297500
H	-4.19648900	10.02031600	-2.04346100
Ir	-2.04105800	7.16479000	1.52692600
Cl	-0.18366000	6.33190700	0.39058700
C	-1.83542400	6.20899500	3.45919700
C	-3.03625000	5.75445200	2.80562600
C	-3.92948200	6.90052700	2.61837900
C	-3.26661500	8.06047000	3.12606600
C	-1.96108400	7.64514700	3.62485200
C	-0.68180800	5.36645000	3.89456400
H	0.25447800	5.92443200	3.84783700
H	-0.83295600	5.03023200	4.92625200
C	-3.35474600	4.34890400	2.42461800
H	-2.44775900	3.77601600	2.22826300
H	-3.98609400	4.31564900	1.53527900
H	-3.89579000	3.86317500	3.24564800
C	-5.30471800	6.83475300	2.04328000
H	-5.37639600	6.08039400	1.25899000
H	-5.60878100	7.79273300	1.61966700
H	-6.01865700	6.57361200	2.83326200
C	-3.79734500	9.45618700	3.14619400
H	-4.24538700	9.67462800	4.12205500
H	-4.56549200	9.60044900	2.38460900
H	-3.00359100	10.18507000	2.97046500
C	-0.94678300	8.53980500	4.24932500
H	-1.00265900	9.55262400	3.84692200
H	0.06497800	8.16118400	4.09802300
H	-1.13122900	8.59448400	5.32948000
H	-0.57150200	4.48791000	3.25772900
C	-2.24122300	10.83284900	-1.50926100
H	-2.52805100	11.22013400	-0.52778400
H	-1.20174100	10.50355400	-1.45062300
H	-2.30509600	11.64974000	-2.23112100
H	-2.88843600	9.31291700	-2.93664600

TS7-1

C	-4.98052700	9.97375000	5.86924200
C	-3.85905000	9.81673800	5.05160300
C	-4.01448500	9.44895100	3.71791600
C	-5.29175000	9.24118300	3.17545400
C	-6.41021600	9.38692600	4.00962800
C	-6.25558100	9.75278300	5.34540000
H	-4.86147000	10.25802800	6.91066200
H	-2.86432100	9.97928600	5.45631800
H	-3.15661500	9.30879500	3.07031500
H	-7.40313800	9.19055200	3.62055400
H	-7.13025700	9.85666900	5.98058800
C	-5.40182000	8.85501500	1.73314400
H	-7.58360500	7.43081700	0.05722800
C	-6.58403600	9.10995800	1.00697300
C	-6.94530700	8.28012100	-0.21784600
H	-7.43401400	9.45299700	1.59245700
H	-7.49093000	8.87065800	-0.96171800
H	-6.05635600	7.87694000	-0.70694500
O	-4.31980900	8.40244200	1.19975200
H	-4.11945000	8.77060100	-0.04442800
O	-3.97640200	9.30384400	-1.02233700
H	-3.04290300	9.53687000	-1.10271100
H	-4.69591600	10.28223000	-0.82345900
O	-5.53205200	11.05023600	-0.45497100
H	-5.13100100	11.74957900	0.08012200
H	-6.05544700	10.32922200	0.29948800

TS7-2

Ir	-4.17214100	-0.46056300	0.53039700
Cl	-5.37044900	-0.80349000	2.55295100
C	-4.96228400	0.36049700	-1.32581500

C	-3.72983100	-0.30943700	-1.68094200
C	-3.84635900	-1.68391700	-1.29090700
C	-5.16813300	-1.89091800	-0.70929700
C	-5.87585000	-0.61997300	-0.76615800
C	-5.24861300	1.80583700	-1.54904300
H	-6.06552900	2.15384800	-0.91602500
H	-5.53238500	1.96909300	-2.59547100
C	-2.56894700	0.27188100	-2.41322300
H	-2.61667200	-0.04320100	-3.46215000
H	-2.58459700	1.36130900	-2.38691500
H	-1.62442000	-0.08370800	-1.99752200
C	-2.78809100	-2.71055000	-1.50271800
H	-1.80109500	-2.31387800	-1.25862700
H	-2.96799100	-3.60815100	-0.91169700
H	-2.77286500	-2.99555200	-2.56113500
C	-5.74150400	-3.18831400	-0.24892400
H	-4.95996900	-3.86350600	0.10310400
H	-6.44894200	-3.03346500	0.56677200
H	-6.26601900	-3.67751600	-1.07809200
C	-7.29434300	-0.39510700	-0.36645700
H	-7.54968900	-0.97840900	0.51909200
H	-7.48576800	0.65536000	-0.14384200
H	-7.95452800	-0.69733300	-1.18800100
H	-4.36662900	2.41315700	-1.33619900
H	-1.49719600	-3.05986500	1.02608800
C	-2.06930400	-2.75213400	1.89882200
C	-2.04346400	-1.23416700	2.07944100
H	-1.62091600	-3.24340200	2.76655100
H	-2.71498100	-0.86185100	2.84548000
H	-3.09733900	-3.11398000	1.81569700
C	-1.62187500	-0.30585900	1.03820200
O	-0.79724200	-0.60027000	0.14110300
C	-2.12877200	1.11647600	1.08242200
C	-3.15195000	1.54909200	1.96541200
C	-1.60980900	2.03670300	0.14410600
C	-3.67755800	2.84979700	1.84893700
H	-3.47268800	0.94426600	2.80228700
C	-2.11288400	3.32443800	0.06700300
H	-0.81185400	1.70194400	-0.50648500
C	-3.16886600	3.72949700	0.90549800
H	-4.46272400	3.16123400	2.52944000
H	-1.69037000	4.02768900	-0.64403000
H	-3.56338900	4.73759400	0.83023100
H	-0.85031800	-1.13186600	2.68074000
O	0.36267000	-1.34961900	3.11162200
H	0.82843500	-1.83659700	2.35931600
O	0.89667600	-2.50616800	0.90050300
H	0.32032700	-1.85168000	0.44037200
C	1.85004800	-3.06938400	-0.03397500
H	1.86083100	-4.14938100	0.14697100
C	1.13285800	-0.20264200	3.61879100
H	1.59363200	-0.55391900	4.54638500
C	1.34310900	-2.81015000	-1.44048800
C	1.35687100	-1.51473500	-1.97768900
C	0.78021400	-3.84797000	-2.19016100
C	0.81569100	-1.26715200	-3.23786900
H	1.77968200	-0.69712900	-1.40289300
C	0.24132000	-3.60295500	-3.45493900
H	0.76794100	-4.85462500	-1.78149300
C	0.25532100	-2.31024800	-3.98028200
H	0.84156800	-0.26175900	-3.64807200
H	-0.18009900	-4.42147900	-4.03060700
H	-0.15327000	-2.11839700	-4.96789500
C	2.21534900	0.20116600	2.64197800
C	3.55637600	-0.03023400	2.96236500
C	1.90404700	0.81677600	1.42129100
C	4.57392500	0.35813800	2.08980000
H	3.80649300	-0.51326900	3.90262900
C	2.91843700	1.20599700	0.54956500
H	0.87197600	0.98618100	1.14262200
C	4.25735800	0.98103700	0.88272200
H	5.61049100	0.17683300	2.35479900
H	2.66688900	1.69192500	-0.38857200
H	5.04668900	1.28983100	0.20486400
C	3.21866800	-2.51420600	0.23910100
C	4.25672000	-3.26500600	0.60095000

H	3.32857000	-1.43807100	0.14593200
H	5.23107200	-2.82850600	0.79609100
H	4.17312000	-4.34360200	0.71081600
C	0.17065200	0.90850800	3.93706600
C	-0.19533200	1.22333700	5.17828500
H	-0.21246900	1.47889000	3.09415800
H	-0.87943100	2.04162100	5.37950600
H	0.18381400	0.68017300	6.03993300

TS7-Cl-1

H	1.62366900	3.00090700	2.25751800
C	0.54082500	3.11842200	2.33739400
C	0.04166500	3.99761000	1.21125700
H	0.08373500	2.12314600	2.27074700
H	0.38313000	3.70479200	0.22436500
H	0.32125100	3.53914800	3.31902900
C	-1.26860100	4.55338500	1.22988400
O	-1.85354800	4.93447900	2.33028600
C	-2.03096000	4.82710600	0.00366400
C	-1.76711400	4.15205700	-1.20087400
C	-3.06071400	5.78470100	0.04048300
C	-2.50800000	4.43805600	-2.34289900
H	-0.99943600	3.38798100	-1.24125500
C	-3.79600100	6.06993600	-1.10409000
H	-3.25732900	6.30242100	0.97128300
C	-3.52071400	5.39875900	-2.29825000
H	-2.29936000	3.90870600	-3.26694600
H	-4.58106200	6.81855300	-1.06915400
H	-4.09549700	5.62211900	-3.19184700
C	1.91016500	8.01096100	3.39151300
C	2.78739100	9.24231400	3.63689500
C	3.56440800	9.41334800	2.32569700
C	3.06657900	8.26502700	1.42696900
N	2.12459500	7.53761000	2.14374500
H	2.14347400	10.09129200	3.88450400
H	4.64778300	9.32273200	2.44229000
Cl	1.05448500	5.84517700	1.50225600
O	3.43428500	8.03999500	0.29350900
O	1.13204100	7.55282800	4.24004700
H	3.37126600	10.36349200	1.82029800
H	3.42234200	9.05519100	4.50770100
H	-1.23129600	5.08678500	3.15403000
O	-0.45805300	5.47475300	4.34480100
H	0.19871200	6.22055100	4.18054700
H	-1.00238800	5.77232100	5.08404400

Int0-H2O

Ir	-0.11073539	0.44612588	-0.03114855
Cl	-2.41932614	1.22501909	-0.02281771
Cl	0.58172314	2.31064156	-1.44272036
C	-0.25205217	-1.49295254	0.94036288
C	1.10719142	-0.99752984	1.05769744
C	1.65307662	-0.80959072	-0.26239129
C	0.61663714	-1.18257189	-1.21768980
C	-0.54140037	-1.62390014	-0.47441915
C	-1.16656451	-1.87060934	2.06019434
H	-0.91906827	-1.32023838	2.97030484
H	-2.20278829	-1.63993753	1.80525769
C	1.80446665	-0.70303522	2.34543213
H	2.25445104	-1.61857472	2.74698969
H	1.10618153	-0.31173034	3.08753018
H	2.59631539	0.03480974	2.20730772
C	3.02819764	-0.34782500	-0.61955078
H	3.50302337	0.16583574	0.21843635
H	2.99208890	0.35155366	-1.45756272
H	3.66028742	-1.19851898	-0.90043775
C	0.76816979	-1.15677216	-2.70351598
H	1.26090542	-2.07312734	-3.04993907
H	1.36652208	-0.29817242	-3.01260447
H	-0.20164294	-1.07841022	-3.19701107
C	-1.82561936	-2.12111389	-1.05150551
H	-1.97237023	-1.74835258	-2.06597446
H	-2.67523139	-1.79042840	-0.45255846
H	-1.81886719	-3.21720936	-1.08156643
H	-1.09398009	-2.94242631	2.28084868
O	-0.03269817	2.15649781	1.45847858

H	0.33583375	2.81083209	0.82866269
H	-0.99733697	2.33134800	1.44445810

Int0-actone

Ir	-0.09243700	0.23460700	0.12428700
Cl	-2.44094900	0.87991100	0.23768600
Cl	0.43285600	2.02334400	-1.43615900
C	-0.50982400	-1.86594800	0.59397500
C	0.66458300	-1.44901300	1.29899200
C	1.67082200	-1.03089900	0.32559300
C	1.10526700	-1.20704200	-0.98560500
C	-0.26770700	-1.65465700	-0.83271600
C	-1.77855300	-2.40144200	1.17071500
H	-1.79836200	-2.29814300	2.25682700
H	-2.63697100	-1.85777700	0.76923800
C	0.86073500	-1.41812400	2.77975800
H	1.50892300	-2.24269400	3.09823200
H	-0.09069000	-1.50356500	3.30669200
H	1.32735500	-0.47752900	3.08175100
C	3.05864900	-0.58037800	0.64934600
H	3.07171100	-0.00621000	1.57868200
H	3.45479100	0.05855100	-0.14184400
H	3.73209700	-1.43787100	0.76943300
C	1.78676600	-0.93982600	-2.28586400
H	2.21764500	-1.87158900	-2.67199600
H	2.58342600	-0.20426700	-2.17252700
H	1.08305400	-0.54660300	-3.02014600
C	-1.22274900	-1.99558200	-1.92992200
H	-1.00632300	-1.41406700	-2.82764600
H	-2.24614200	-1.76755800	-1.62660800
H	-1.15942500	-3.06127100	-2.18234400
H	-1.88451600	-3.46438700	0.92478800
C	0.04582100	2.83543100	2.07307900
O	0.35510800	1.67053700	1.79520400
C	0.58733500	3.42220300	3.34995300
H	1.21227100	2.70071900	3.87702900
H	1.16547700	4.32458000	3.12011500
H	-0.24620700	3.73598900	3.98884500
C	-0.82967800	3.68857400	1.21775600
H	-1.77945700	3.16754000	1.06088800
H	-0.98512800	4.67700500	1.65273900
H	-0.37943300	3.75829500	0.22237600

REFERENCES

[1] E. Erbing, A. Vázquez-Romero, A. Bermejo Gómez, A. E. Platero-Prats, F. Carson, X. Zou, P. Tolstoy, B. Martín-Matute, *Chem. Eur. J.* **2016**, *22*, 15659-15663.
[2] B. Spiegelberg, A. Dell'Acqua, T. Xia, A. Spannenberg, S. Tin, S. Hinze, J. G.deVries, *Chem. Eur. J.* **2019**, *25*, 7820-7825.
[3] A. Sanz-Marco, S. Martinez-Erro, M. Pauze, E. Gómez-Bengoa, B. Martín-Matute, *Nat. Commun.* **2019**, *10*, 5244-5253.
[4] W. Xu, Y. Zhou, R. Wang, G. Wu, P. Chen, *Org. Biomol. Chem.* **2012**, *10*, 367-371.
[5] N. Ahlsten, A. Bermejo Gómez, B. Martín-Matute, *Angew. Chem., Int. Ed.* **2013**, *52*, 6273-6276.
